# GTPBP8 modulates mitochondrial fission through a Drp1-dependent process

**DOI:** 10.1242/jcs.261612

**Published:** 2024-04-30

**Authors:** Xiumei He, Liang Wang, Hoi Ying Tsang, Xiaonan Liu, Xiaofeng Yang, Shiming Pu, Ziqi Guo, Cheng Yang, Qiong Wu, Zuping Zhou, Xiaobo Cen, Hongxia Zhao

**Affiliations:** ^1^ School of Life Sciences, Guangxi Normal University, Guilin 541004, China; ^2^ Mental Health Center and National Chengdu Center for Safety Evaluation of Drugs, State Key Laboratory of Biotherapy, West China Hospital of Sichuan University, Chengdu 610041, China; ^3^ Guangxi Universities Key Laboratory of Stem Cell and Biopharmaceutical Technology, Guangxi Normal University, Guilin 541004, China; ^4^ Faculty of Biological and Environmental Sciences, University of Helsinki, 00014 Helsinki, Finland; ^5^ Department of Physiology, Faculty of Medical Sciences in Katowice, Medical University of Silesia in Katowice, Katowice 40752, Poland

**Keywords:** GTPBP8, Mitochondria, Fission, Drp1, Phosphorylation

## Abstract

Mitochondrial fission is a tightly regulated process involving multiple proteins and cell signaling. Despite extensive studies on mitochondrial fission factors, our understanding of the regulatory mechanisms remains limited. This study shows the critical role of a mitochondrial GTPase, GTPBP8, in orchestrating mitochondrial fission in mammalian cells. Depletion of GTPBP8 resulted in drastic elongation and interconnectedness of mitochondria. Conversely, overexpression of GTPBP8 shifted mitochondrial morphology from tubular to fragmented. Notably, the induced mitochondrial fragmentation from GTPBP8 overexpression was inhibited in cells either depleted of the mitochondrial fission protein Drp1 (also known as DNM1L) or carrying mutated forms of Drp1. Importantly, downregulation of GTPBP8 caused an increase in oxidative stress, modulating cell signaling involved in the increased phosphorylation of Drp1 at Ser637. This phosphorylation hindered the recruitment of Drp1 to mitochondria, leading to mitochondrial fission defects. By contrast, GTPBP8 overexpression triggered enhanced recruitment and assembly of Drp1 at mitochondria. In summary, our study illuminates the cellular function of GTPBP8 as a pivotal modulator of the mitochondrial division apparatus, inherently reliant on its influence on Drp1.

## Teaser

**Summary:** By modulating the phosphorylation of Drp1, GTPBP8 influences mitochondrial fission dynamics and contributes to the overall maintenance of mitochondrial homeostasis.

## INTRODUCTION

Mitochondria are dynamic organelles that undergo continuous changes in morphology, driven by processes such as division, fusion and movement throughout the cell. Collectively, these processes constitute mitochondrial dynamics (El-Hattab et al., 2018; Fenton et al., 2021). They play a fundamental role in maintaining proper mitochondrial function and quality control (Mishra and Chan, 2016). Through fusion and fission, mitochondria exchange contents, redistribute damaged components and adapt to changing cellular energy demands (Gao and Hu, 2021; Silva Ramos et al., 2019). This intricate regulation of mitochondrial dynamics significantly contributes to overall cellular health and function. The physiological relevance of mitochondrial dynamics came to light with the discovery that alterations in these processes are associated with various human pathologies, including cancer and neurodegeneration (Chan, 2006; Kraus and Ryan, 2017; Quiles and Gustafsson, 2022; Wang et al., 2023).

Although considerable research has been dedicated to unraveling the complexities of mitochondrial fusion, the mechanisms governing mitochondrial fission remain less elucidated. Mitochondrial fission is a finely tuned process involving the orchestrated interplay of various factors. Key factors in the mitochondrial fission process include the GTPase dynamin-related protein-1 (Drp1; also known as DNM1L), fission-1 (Fis1), mitochondrial fission factor (Mff), and other fission factors located on the mitochondrial outer membrane (Cerveny et al., 2007; Mishra and Chan, 2016; Tilokani et al., 2018). The initiation of mitochondrial fission involves the recruitment of the cytosolic protein Drp1 to specific sites on the outer mitochondrial membrane (OMM), facilitated by distinct adaptors such as Mff, Fis1, MiD49 (MIEF2) and MiD51 (MIEF1) (Losón et al., 2013; Palmer et al., 2011). Following recruitment, Drp1 assembles into helical or ring-like oligomers around the mitochondrial tubule, leading to the constriction of the membrane at the fission site, resembling a ‘garrote-like’ effect (Kraus and Ryan, 2017; Ramachandran, 2018). After constriction, the final scission of the OMM occurs through the action of dynamin 2 (Dnm2), which forms a collar-like structure at the constriction site, facilitating the complete separation of the two daughter mitochondria (Ferguson and De Camilli, 2012). Moreover, the endoplasmic reticulum, actin polymerization and Ca^2+^ influx contribute to the fission process (Chakrabarti et al., 2018; Goldbeter et al., 2013). Opa1, located in the inner membrane facing the intermembrane space, has been suggested to play a role in the fission process (Anand et al., 2014). However, the significance of its role in this process is not fully understood and remains controversial.

Drp1 is a key regulator of mitochondrial fission, and its activity and localization are tightly controlled through various post-translational modifications, including phosphorylation, SUMOylation, ubiquitylation, S-nitrosylation and acetylation. These modifications serve as crucial molecular switches that modulate the localization, oligomerization and activity of Drp1 on the mitochondrial outer membrane. The interplay between these modifications plays a crucial role in determining the balance between mitochondrial fusion and fission, thereby influencing overall mitochondrial dynamics and morphology (Cerveny et al., 2007; Chang and Blackstone, 2010; Das et al., 2022; Figueroa-Romero et al., 2009; Jahani-Asl and Slack, 2007; Ko et al., 2016; Mishra and Chan, 2016; Quiles and Gustafsson, 2022; Tilokani et al., 2018). Drp1 is primarily a cytosolic GTPase, with only ∼3% being recruited to the outer membrane of mitochondria in response to specific fission signals (Smirnova et al., 2001). Phosphorylation of Drp1 at specific serine residues has a major impact on its activity and function. Drp1 phosphorylation at Ser616 (Drp1 S616-PO**_4_**) by kinases such as cyclin-dependent kinase (CDK) 1–cyclin B or CDK5 promotes the recruitment of Drp1 to mitochondria and facilitates mitochondrial fission, often occurring during cell division or under stress conditions that demand increased fission (Liesa et al., 2009; Taguchi et al., 2007). Conversely, phosphorylation at Ser637 (Drp1 S637-PO**_4_**) by protein kinase A (PKA) inhibits the translocation of Drp1 to mitochondria, reducing fission and promoting mitochondrial fusion (Kashatus et al., 2011; Wang et al., 2012). By contrast, the dephosphorylation of Drp1 S637-PO_4_ by calcineurin facilitates its translocation to mitochondria, subsequently enhancing mitochondrial fission (Campello and Scorrano, 2010). These phosphorylation and dephosphorylation events play crucial roles as switches, modulating the localization, oligomerization and activity of Drp1 on the mitochondrial outer membrane.

Although much of the current research has focused on factors and processes related to the mitochondrial outer membrane in the regulation of fission, the specific role of inner membrane proteins, particularly those localized to the matrix side of the inner membrane, remains a significant gap in our understanding. Further investigation and studies are necessary to uncover the potential contributions of these inner membrane proteins to the complex process of mitochondrial fission. In this study, we have unveiled the cellular role of a novel GTP-binding protein, GTPBP8, within mitochondria. Through a series of loss-of-function and gain-of-function experiments, we demonstrate that GTPBP8 is intricately involved in the process of mitochondrial fission. The depletion of GTPBP8 leads to an elevation in oxidative stress and impaired mitochondrial function. This mitochondrial dysfunction further influenced cell signaling involved in the phosphorylation of Drp1, leading to the increased phosphorylation of Drp1 at Ser637, which hinders the recruitment of Drp1 from the cytosol to the mitochondrial outer membrane. These combined effects contribute to the observed hyperfused mitochondrial morphology in the absence of GTPBP8. Moreover, the induced mitochondrial fragmentation resulting from GTPBP8 overexpression is impeded in cells depleted of Drp1 or carrying mutated forms of Drp1, unequivocally indicating that the role for GTPBP8 in mitochondrial fission relies on Drp1. Collectively, our findings underscore the crucial importance of GTPBP8 in modulating mitochondrial fission by exerting influence over the recruitment and activity of Drp1.

## RESULTS

### GTPBP8 knockdown influences mitochondrial morphology

**Fig. 1.**
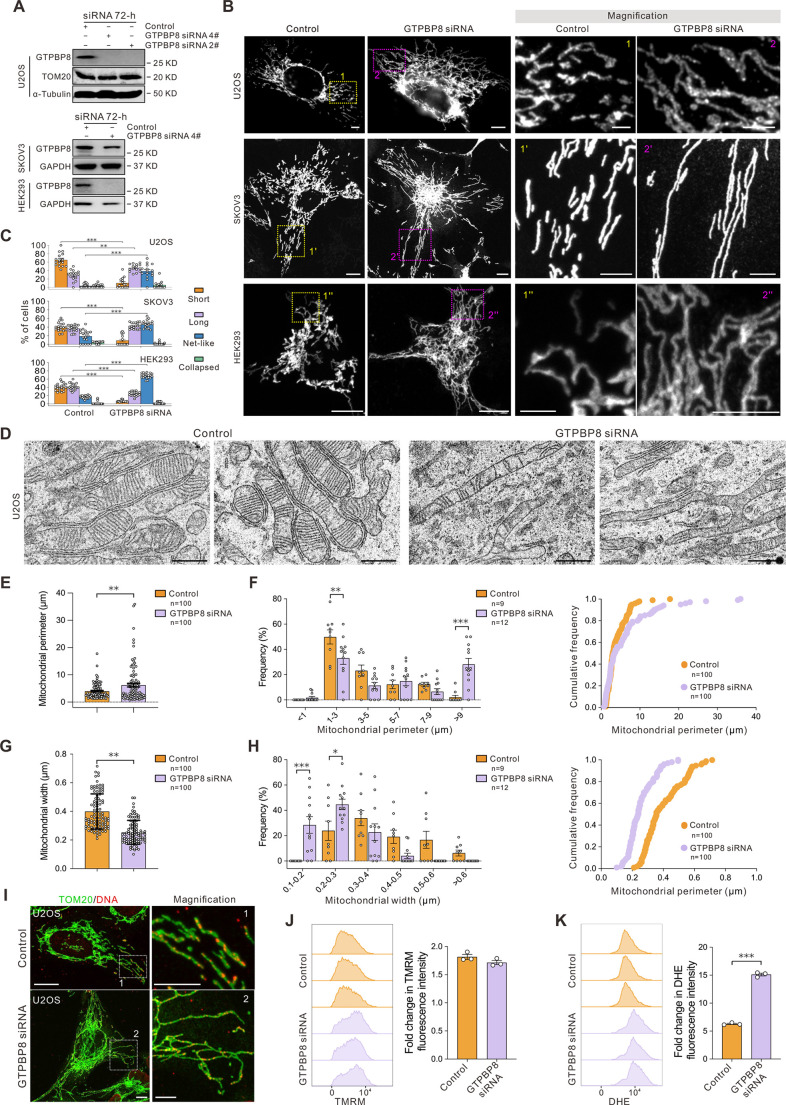
**Knockdown of mitochondrial protein GTPBP8 causes mitochondrial elongation.** (A) Western blot of cell lysates after transfection with control and GTPBP8 siRNA for 72 h. TOM20, α-Tubulin, and GAPDH are loading controls. Images shown representative of three repeats. (B) The representative images show mitochondrial morphology in U2OS, SKOV3 and HEK293 cells treated with control and GTPBP8 siRNA for 72 h. Mitochondria were visualized by immunofluorescence against TOM20 through overexpressed Mito–YFP. Scale bars: 10 μm (left panels); 5 μm (magnifications). (C) Scoring of mitochondrial morphologies for control and GTPBP8-knockdown cells. Mitochondria in each cell were scored into one of four morphological categories. For U2OS cells, control, *n*=16 microscopic fields; GTPBP8 siRNA, *n*=15 microscopic fields. Two-way repeated measured ANOVA, followed by Dunnett's multiple comparisons test, *F*_treatment_(1, 29)=0.3444, *P*=0.5618; *F*_morphology_(2.255, 65.39)=43.83, *P*<0.0001; *F*_interaction_(3, 87)=66.92, *P*<0.0001; SKOV3 cells, Control, *n*=19 microscopic fields; GTPBP8 siRNA, *n*=19 microscopic fields. Two-way repeated measured ANOVA, followed by Dunnett's multiple comparisons test, *F*_treatment_(1, 36)=0.02993, *P*=0.8636; *F*_morphology_(3, 108)=88.94, *P*<0.0001; *F*_interaction_(3, 108)=53.24, *P*<0.0001; HEK293 cells, Control, *n*=20 microscopic fields; GTPBP8 siRNA, *n*=20 microscopic fields. Two-way repeated measured ANOVA, followed by Dunnett's multiple comparisons test, *F*_treatment_(1, 76)=4.532 × 10^−13^, *P*>0.9999; *F*_morphology_(3, 76)=454.5, *P*<0.0001; *F*_interaction_(3, 76)=366.2, *P*<0.0001. (D) Electron micrographs of mitochondria in U2OS cells treated with control and GTPBP8 siRNA for 72 h. Scale bar: 1 μm. (E) Bar graph showing the mitochondrial perimeter (μm) measured from TEM images. *n*=100 mitochondria/group, unpaired two-tailed *t*-test, *t*(198)=3.048, *P*=0.0026. (F) Proportion of the mitochondria that have a perimeter within 2 μm bins (left panel). Control, *n*=9 microscopic fields; GTPBP8 siRNA, *n*=12 microscopic fields. Two-way repeated measured ANOVA, followed by Dunnett's multiple comparisons test, *F*_perimeter_(5, 95)=25.93, *P*<0.0001; *F*_treatment_(1, 19)=0.7403, *P*=0.4003; *F*_interaction_(5, 95)=7.935, *P*<0.0001. Cumulative frequency of mitochondrial perimeter (right panel). *n*=100 mitochondria/group, two-sample Wilcoxon test, *P*<0.0001. (G) Bar graph showing the mitochondrial width (μm) measured from TEM images. *n*=100 mitochondria/group, unpaired two-tailed *t*-test, *t*(198)=9.787, *P*<0.0001. (H) Proportion of the mitochondria that have a width within 0.1 μm bins (left panel). Control, *n*=9 microscopic fields, GTPBP8 siRNA, *n*=12 microscopic fields. Two-way repeated measured ANOVA, followed by Dunnett's multiple comparisons test, *F*_width_(5, 95)=10.78, *P*<0.0001; *F*_treatment_(1, 19)=3.744, *P*=0.0680; *F*_interaction_(5, 95)=7.038, *P*<0.0001. Cumulative frequency of mitochondrial width (right panel). *n*=100 mitochondria/group, two-sample Wilcoxon test, *P*<0.0001. (I) The representative images show DNA (red) and mitochondria (green) in U2OS treated with control and GTPBP8 siRNA for 72 h. Mitochondria and DNA were visualized by immunofluorescence against TOM20 and DNA. Scale bars: 10 μm (left panels) 5 μm (magnifications). (J) Flow cytometry histogram and quantification of TMRM fluorescence in U2OS cells treated with control and GTPBP8 siRNA for 72 h. *n*=3 replicates/group, unpaired two-tailed *t*-test, *t*(4)=1.782, *P*=0.1494. (K) Flow cytometry histogram and quantification of DHE fluorescence in U2OS cells treated with control and GTPBP8 siRNA for 72 h. *n*=3 replicates/group, unpaired two-tailed *t*-test, *t*(4)=32.69, *P*<0.0001. All error bars are mean±s.e.m. *P<0.05; **P<0.01; ***P<0.001 compared to control.

Human GTPBP8 is a homolog of YihA in *Escherichia coli* and YsxC in *Bacillus subtilis* (Gupta et al., 2020; Ruzheinikov et al., 2004). Ortholog analysis, performed using the Princeton Protein Orthology Database (P-POD), revealed that GTPBP8 is a conserved GTP-binding protein that belongs to the P-loop NTPase superfamily (Fig. S1A). We recently reported that GTPBP8 resides in the matrix side of the mitochondrial inner membrane and is peripherally associated with the inner membrane (Wang et al., 2023). To investigate the functions of GTPBP8 within mitochondria, we conducted a comprehensive exploration of its impact through loss-of-function experiments in mammalian cells, utilizing siRNA-mediated protein knockdown. After transfecting U2OS cells with GTPBP8 siRNA (siRNAs 2# and 4#) for 72 h, we observed a significant reduction in the steady-state level of GTPBP8 (Fig. 1A). Subsequently, we assessed mitochondrial morphology by performing immunofluorescence analysis using the mitochondrial marker TOM20 (also known as TOMM20). The knockdown of GTPBP8 resulted in the fusion of elongated and interconnected mitochondria, in stark contrast to the moderately elongated and interconnected mitochondria observed in control cells (Fig. 1B). To quantitatively evaluate the impact of GTPBP8 loss on mitochondrial morphology, we employed a morphology scoring assay, categorizing mitochondria into distinct groups – as having short tubules, long tubules, a net-like structures or as collapsed (Fig. S1B) (Losón et al., 2013). Although control cells primarily exhibited short mitochondria, GTPBP8 knockdown led to a higher proportion of cells displaying long or net-like mitochondria (Fig. 1C). This effect was not limited to U2OS cells, knockdown of GTPBP8 with GTPBP8 siRNA 4# for 72 h in SKOV3 and HEK293 cells also caused a higher proportion of cells with net-like mitochondria compared to corresponding control cells (Fig. 1A–C; Fig. S1B). These findings imply that the mitochondrial protein GTPBP8 plays a pivotal role in regulating mitochondrial dynamics.

To further assess alterations in mitochondrial morphology and explore the potential effects on mitochondrial ultrastructure upon GTPBP8 knockdown, we employed transmission electron microscopy (TEM) to visualize mitochondria. In comparison to the short, rod-shaped mitochondrial structure observed in control U2OS cells, a substantial proportion of mitochondria in GTPBP8 knockdown cells exhibited a hyper-fused, elongated and interconnected morphology (Fig. 1D; Fig. S1C). The quantification of mitochondrial size revealed a notable change, with the average mitochondrial perimeter expanding from 4.033 μm in the control group to 6.248 μm in the GTPBP8 knockdown group (Fig. 1E). The changes in mitochondrial perimeter primarily resulted from a reduction in the number of mitochondria with a perimeter ranging from 3 to 5 μm, coupled with an increase in the population of mitochondria exhibiting a perimeter exceeding 9 μm (Fig. 1F). Moreover, the mitochondrial width was reduced in the GTPBP8 knockdown cells. Specifically, the average mitochondrial width decreased from 0.399 μm in the control group to 0.254 μm in the GTPBP8 knockdown group (Fig. 1G). The occurrence of mitochondria with a width less than 0.3 μm was higher in the GTPBP8 knockdown cells (Fig. 1H). However, no discernible alterations were observed in mitochondrial architecture following GTPBP8 knockdown, with the exception of fewer crista inside mitochondria (Fig. S1D). Similar to the control group, the outer membrane maintained its smooth appearance enveloping the mitochondria, whereas the inner membrane displayed the extensive folding characterized by numerous cristae. The cristae themselves retained their characteristic tubule- or sheet-like structures, even in the absence of GTPBP8 (Fig. 1D). These findings uncover that the depletion of GTPBP8 results in elongated and interconnected mitochondrial networks, with no discernible impact on the architecture of the inner mitochondrial membrane.

GTPBP8 is a mitoribosomal assembly factor, and its knockdown causes mitoribosomal assembly and protein translation defects, leading to impaired mitochondrial function, including reduced respiratory capacity (Wang et al., 2023). To explore whether the loss of other mitoribosomal assembly factors elicits a similar effect on mitochondrial morphology, we examined changes in mitochondrial morphology following the depletion of three other mitoribosomal assembly factors, GTPBP5, GTPBP7 and GTPBP10 (Kotani et al., 2013; Lavdovskaia et al., 2018; Maiti et al., 2020) (Fig. S2A,B). The results showed that only GTPBP5 knockdown resulted in a net-like mitochondrial morphology, whereas mitochondria in cells with GTPBP7 and GTPBP10 knockdown still exhibited short tubules (Fig. S2C,D). Moreover, a small proportion of GTPBP7 knockdown cells did display a fragmented mitochondrial morphology (Fig. S2C). These data suggest that not all cells in a low-energy state or with compromised mitochondrial function present elongated or interconnected mitochondrial morphology.

In addition to energy state, mitochondrial dynamics is also influenced by mitochondrial DNA (mtDNA), cellular reactive oxygen species (ROS) and mitochondrial membrane potential (Ni et al., 2015; Panigrahi et al., 2022; Yang et al., 2015). We thus examined the distribution of mtDNA, mitochondrial membrane potential and the production of ROS after depletion of GTPBP8. In contrast to what was seen in control cells, GTPBP8 knockdown caused an evident increase in oxidative stress but did not affect mitochondrial membrane potential or the homogeneous localization of mtDNA foci in mitochondria (Fig. 1I–K). Together, these data suggest that GTPBP8 depletion causes a compromised energy state and oxidative stress. As a consequence of impaired mitochondrial functions, it can be assumed that mitochondrial dynamics are affected.

### GTPBP8 knockdown causes a decrease in mitochondrial fission events

**Fig. 2.**
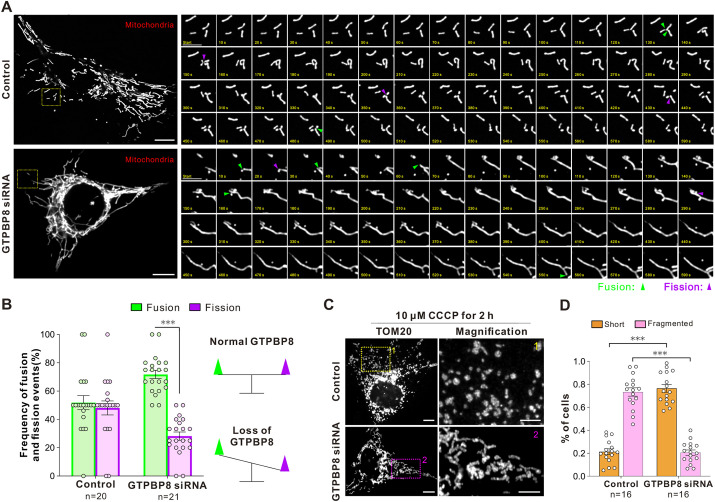
**GTPBP8 knockdown causes a decrease in mitochondrial fission events.** (A) Immunofluorescence images from live-cell imaging showing mitochondrial fission and fusion events in U2OS cells treated with control and GTPBP8 siRNA for 72 h. Examples of cells expressing Mito–DsRed are shown on the left, and magnified regions on the right represent time-lapse images at the different time points. Fission and fusion events are labeled with magenta and green arrowheads, respectively. Scale bar: 10 μm (cell images); 5 μm (magnifications). (B) Bar graph showing the frequency of mitochondrial fusion and fission events during 10 min of recording (left). Schematic diagram of mitochondrial dynamics in U2OS cells upon loss of GTPBP8 (right). Control, *n*=20 microscopic fields; GTPBP8 siRNA, *n*=21 microscopic fields. Two-way repeated measured ANOVA, followed by Dunnett's multiple comparisons test, *F*_dynamic_(1, 78)=34.94, *P*<0.0001; *F*_treatment_(1, 78)=0, *P*>0.9999; *F*_interaction_(1, 78)=24.67, *P*<0.0001. (C) Immunofluorescence images showing the mitochondrial morphology in the control and GTPBP8 knockdown cells in response to 10 μM CCCP treatment for 2 h. Mitochondria were visualized by probing for TOM20. Scale bar: 10 μm (cell images); 5 μm (magnifications). (D) Bar graph showing the assessed mitochondrial morphology for control and GTPBP8 knockdown cells treated as in B. The mitochondria in each cell were scored into one of two morphological categories, fragmented and short tubular-like (short) morphology by a researcher unaware of the experimental conditions. *n*=16 microscopic fields/group. Two-way repeated measured ANOVA, followed by Dunnett's multiple comparisons test, *F*_morphology_(1, 30)=0.4194, *P*=0.5222; *F*_treatment_(1, 30)=0.1506, *P*=0.7007; *F*_interaction_(1, 30)=358.1, *P*<0.0001. All error bars are mean±s.e.m. ***P<0.001 compared to control.

How GTPBP8 is involved in mitochondrial dynamics was then investigated. After the knockdown of GTPBP8 for 72 h, mitochondrial fission and fusion events were assessed through time-lapse live-cell imaging. Mitochondria in control and GTPBP8 knockdown cells were visualized by expressing the Mito–DsRed constructs for 48 h, and the behavior of mitochondria was traced at 10-s intervals (Movies 1 and 2). The quantification of fission and fusion events within a fixed time frame revealed notable changes. GTPBP8 knockdown led to a reduction in mitochondrial fission events and an increase in fusion events compared to what was seen in control cells (Fig. 2A). The total number of fission and fusion events within a 10-min mitochondrial tracking window was then manually counted. Compared to the balanced occurrence of fission and fusion in the control cells, the frequency of fission events was reduced in the hyperfused mitochondria caused by GTPBP8 depletion (Fig. 2B). This indicates that loss of GTPBP8 causes an imbalance between mitochondrial fusion and fission, which leads to elongated and interconnected mitochondrial networks. These findings provide additional evidence that the depletion of GTPBP8 alters mitochondrial dynamics.

The effects of GTPBP8 depletion on imbalanced mitochondrial dynamics were further elucidated by treating control and GTPBP8 knockdown cells to carbonyl cyanide m-chlorophenylhydrazone (CCCP), a compound that depolarizes the mitochondrial membrane and promotes unopposed Drp1-mediated mitochondrial fission (Palmer et al., 2011). Pre-treating the cells with 10 μM CCCP for 2 h before fixation revealed that the majority of mitochondria in control cells exhibited a rounded morphology, whereas a substantial proportion of mitochondria in GTPBP8-depleted cells retained a short tubular morphology (Fig. 2C). Quantitative analysis indicated that GTPBP8 knockdown led to ∼72% reduction in CCCP-induced mitochondrial fragmentation (Fig. 2D). Taken together, these data indicate that GTPBP8 is involved in regulating mitochondrial fission.

### GTPBP8 expression promotes mitochondrial fission

**Fig. 3.**
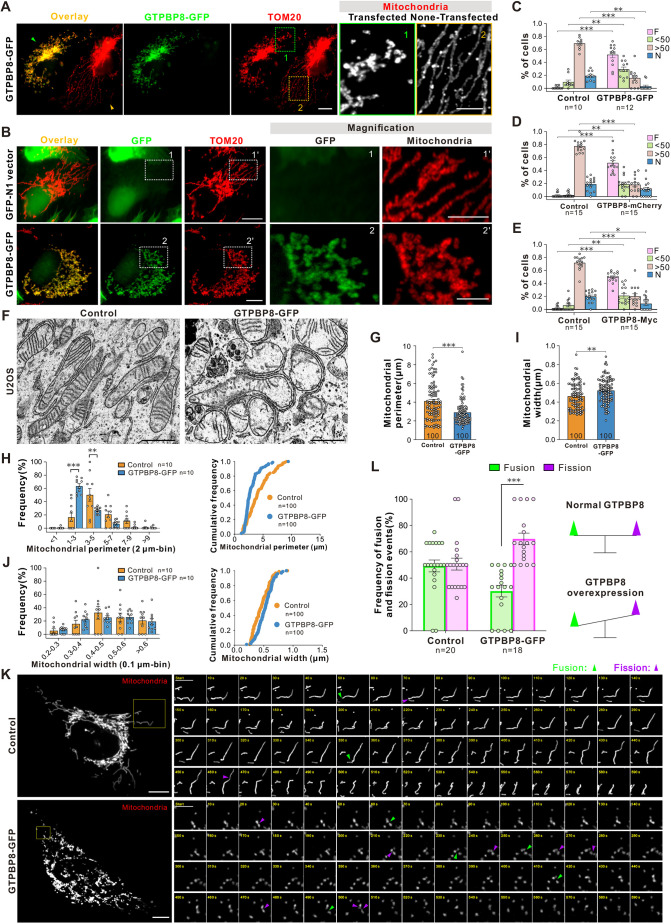
**GTPBP8 expression promotes mitochondrial fission.** (A) Immunofluorescence images showing the mitochondrial morphology in U2OS cells that were transiently transfected with the GTPBP8–GFP construct for 48 h. Mitochondria were visualized by probing for TOM20. The non-transfected and transfected cells are highlighted with orange and green arrowheads, respectively. Scale bars: 10 μm (left panels); 5 μm (magnifications). (B) Immunofluorescence images showing the mitochondrial morphology in U2OS cells that were transiently transfected with the GFP-N1 empty vector and GTPBP8–GFP construct for 48 h. Mitochondria were visualized by probing for TOM20. Scale bars: 10 μm (left panels); 5 μm (magnifications). (C) Scoring of mitochondrial morphologies for GTPBP8–GFP-transfected and control cells. Control, *n*=10 microscopic fields; GTPBP8–GFP, *n*=12 microscopic fields. Two-way repeated measured ANOVA, followed by Dunnett's multiple comparisons test, *F*_treatment_(1, 80)=5.069×10^−13,^ *P*>0.999; *F*_morphology_(3, 80)=32.26, *P*<0.0001; *F*_interaction_(3, 80)=92.27, *P*<0.0001. (D) Scoring of mitochondrial morphologies for GTPBP8–mCherry-transfected and control cells. *n*=15 microscopic fields/group. Two-way repeated measured ANOVA, followed by Dunnett's multiple comparisons test, *F*_treatment_(1, 56)=5.135×10^−11^, *P*>0.999; *F*_morphology_(3, 56)=72.53, *P*<0.0001; *F*_interaction_(3, 56)=153.8, *P*<0.0001. (E) Scoring of mitochondrial morphologies for GTPBP8–Myc-transfected and control cells. *n*=15 microscopic fields/group. Two-way repeated measured ANOVA, followed by Dunnett's multiple comparisons test, *F*_treatment_(1, 56)=2.023×10^−11^, *P*>0.999; *F*_morphology_(3, 56)=61.36, *P*<0.0001; *F*_interaction_(3, 56)=128.9, *P*<0.0001. For C–E, mitochondria in each cell were scored into one of four morphological categories. F, fragmented; <50, <50% of mitochondria are short or long tubules; >50, >50% of mitochondria are short or long tubules; N, net-like. (F) Electron micrographs of mitochondria in U2OS cells transfected with control and GTPBP8–GFP for 48 h. Scale bars: 1 μm. (G) Bar graph showing the mitochondrial perimeter (μm) measured from TEM images. *n*=100 mitochondria/group, unpaired two-tailed *t*-test, *t*(198)=4.949. (H) Proportion of the mitochondria that have a perimeter within a 2 μm bin (left panel). *n*=10 microscopic fields/group. Two-way repeated measured ANOVA, followed by Dunnett's multiple comparisons test, *F*_perimeter_(5, 90)=34.90, *P*<0.0001; *F*_treatment_(1, 18)=4.143, *P*=0.0568; *F*_interaction_(5, 90)=15.98, *P*<0.0001. Cumulative frequency of the mitochondrial perimeter (right panel). *n*=100 mitochondria/group, two-sample Wilcoxon test, *P*<0.0001. (I) Bar graph showing the mitochondrial width (μm) measured from TEM images. *n*=100 mitochondria/group, unpaired two-tailed *t*-test, *t*(198)=3.160, *P*=0.0018. (J) Proportion of the mitochondria that have a mitochondrial width within with 0.1 μm bins (left panel). *n*=10 microscopic fields/group. Two-way repeated measured ANOVA, followed by Dunnett's multiple comparisons test, *F*_width_(6, 108)=13.44, *P*<0.0001; *F*_treatment_(1, 18)=0.1849, *P*=0.6723; *F*_interaction_(6, 108)=0.4007, *P*=0.8771. Cumulative frequency of the mitochondrial width (right panel). *n*=100 mitochondria/group, two-sample Wilcoxon test, *P*<0.0001. (K) Immunofluorescence images from live-cell imaging show mitochondrial fission and fusion events in Mito–DsRed- and GTPBP8–GFP-expressing U2OS cells. Examples of cells expressing Mito-DsRed or GTPBP8-GFP are shown on the left, and magnified regions on the right represent time-lapse images at the different time points. Fission and fusion events are labeled with magenta and green arrows, respectively. Scale bar: 10 μm (cell images); 5 μm (magnifications). (L) Bar graph showing the frequency of mitochondrial fusion and fission events during 10 min of recording (left). Schematic diagram of mitochondrial dynamics in U2OS cells upon overexpression of GTPBP8 (right). Control, *n*=20 microscopic fields; GTPBP8–GFP, *n*=18 microscopic fields. Two-way repeated measured ANOVA, followed by Dunnett's multiple comparisons test, *F*_dynamic_(1, 72)=21.05, *P*<0.0001; *F*_treatment_(1, 72)=0, *P*>0.9999; *F*_interaction_(1, 72)=18.24, *P*<0.0001. All error bars are mean±s.e.m. *P<0.05; **P<0.01; ***P<0.001 compared to control.

To further explore the function of GTPBP8 in governing mitochondrial fission, we expressed a GFP-tagged GTPBP8 construct (GTPBP8–GFP) into U2OS cells for 48 h. In contrast to the prevailing tubular-like mitochondrial morphology observed in non-transfected cells, a substantial portion of mitochondria in cells expressing GTPBP8–GFP exhibited a fragmented appearance (Fig. 3A,B). Since cells transfected with the control GFP vector exhibited a normal tubular morphology, the fragmented mitochondrial morphology observed cannot be attributed to stress induced by the overexpressed GFP tag within cells (Fig. 3B). To quantitatively evaluate the impact of GTPBP8 expression on mitochondrial morphology, we employed a morphology scoring assay, categorizing mitochondria into four morphological categories (Fig. S3A) (Losón et al., 2013). Compared to the control U2OS cells, the proportion of cells with completely fragmented mitochondria (F) and those with less than 50% of long tubular-like mitochondria (<50) was increased, whereas the percentage of cells with more than 50% of long tubular-like mitochondria (>50) and net-like mitochondria (N) was decreased (Fig. 3C). Consistent with the results observed for GTPBP8–GFP expression, the majority of mitochondria in cells expressing GTPBP8–mCherry and GTPBP8–Myc also exhibited a fragmented morphology (Fig. 3D,E; Fig. S3B). Moreover, the effect of GTPBP8 overexpression on mitochondrial morphology was also investigated in SKOV3 and HEK293 cells. Although most of the mitochondria in SKOV3 cells expressing GTPBP8–GFP still exhibited short or long tubular structures, a proportion of cells classified in the short group presented a fragmented mitochondrial morphology (Fig. S3C–E). Similar to what was seen for U2OS cells, an increased proportion of GTPBP8–GFP-expressed HEK293 cells exhibited fragmented mitochondria (Fig. S3C,F). Together, these data suggest that GTPBP8 expression promotes mitochondrial fission.

The changes in mitochondrial morphology and ultrastructure in GTPBP8-expressing cells were further examined and quantified using TEM. Similar to the observations in GTPBP8 knockdown cells, the mitochondrial ultrastructure remained unaffected in cells expressing GTPBP8–GFP. Nevertheless, a notable alteration in mitochondrial size was observed. In contrast to the mitochondria in control cells, the majority of mitochondria in GTPBP8-overexpressed cells displayed round and short structures (Fig. 3F; Fig. S4). The quantification of the mitochondrial perimeter revealed a decrease, with the average mitochondrial perimeter reducing from 4.106 μm in the control group to 2.899 μm in the GTPBP8–GFP-expressing group (Fig. 3G). The observed decrease in the mitochondrial perimeter was predominantly associated with an increase in the population of mitochondria having perimeters within the range of 1 to 3 μm. Conversely, a decline was noted in the population of mitochondria with larger perimeters (Fig. 3H). Furthermore, the introduction of GTPBP8–GFP expression resulted in a pronounced augmentation of mitochondrial width, with the average width transitioning from ∼0.462 μm in the control group to ∼0.522 μm in the cells expressing GTPBP8–GFP (Fig. 3I). The frequency of mitochondria with a width of ∼0.2–0.4 μm was increased in the GTPBP8–GFP-expressing cells (Fig. 3J).

The process of mitochondrial dynamics in GTPBP8-expressing cells was further studied using time-lapse live-cell imaging. Similar to the observed mitochondrial fission and fusion activities in control cells, mitochondria within cells expressing GTPBP8–GFP also exhibited a capacity for fusion and fission events, as depicted in Movies 3 and 4. Subsequently, we quantified the occurrence of fission and fusion events within a fixed time interval. The results revealed that cells expressing GTPBP8–GFP had a notable escalation in mitochondrial dynamics, as evidenced by an augmented frequency of both mitochondrial fission and fusion events (Fig. 3K), in comparison to control cells. Manually counting the fission and fusion events within a 10-min mitochondrial tracking window revealed an increased frequency of fission events in cells expressing GTPBP8–GFP, contrasting with the balanced frequency of fission and fusion in the control cells (Fig. 3L). When considered alongside the observed changes in mitochondrial dynamics resulting from GTPBP8 knockdown, these results strongly emphasize the crucial role of GTPBP8 in governing mitochondrial dynamics.

### GTPBP8-mediated mitochondrial fission is dependent on Drp1

**Fig. 4.**
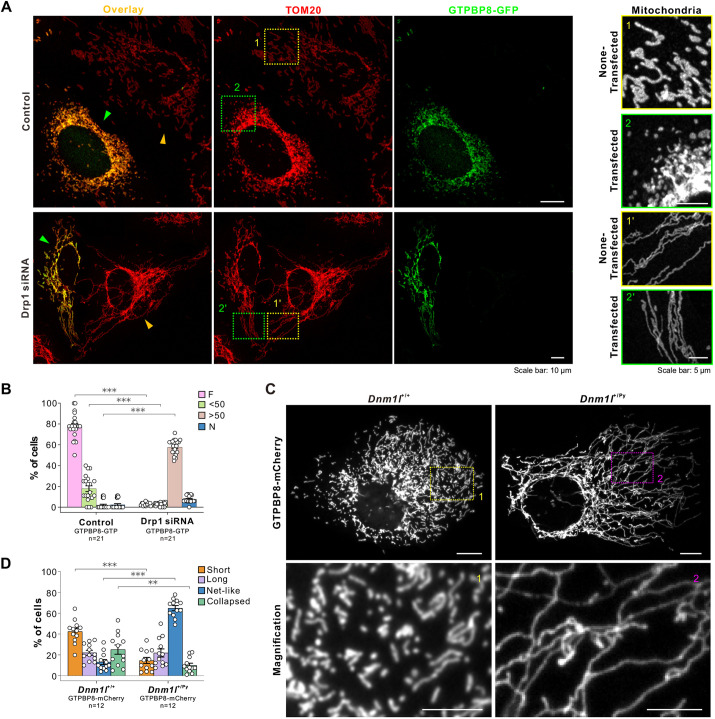
**GTPBP8-mediated mitochondrial fission is dependent on Drp1.** (A) Immunofluorescence images showing the mitochondrial morphology in U2OS cells treated with control and Drp1 siRNA, followed by transient transfection with the GTPBP8–GFP construct. The non-transfected and transfected cells are highlighted with orange and green arrowheads, respectively. Scale bar: 10 μm (cell images); 5 μm (mitochondria). (B) Scoring of mitochondrial morphologies for GTPBP8–GFP-transfected control and Drp1 siRNA cells. Mitochondria in each cell were scored into one of four morphological categories. F, fragmented; <50, <50% of mitochondria are long tubules; >50, >50% of mitochondria are long tubules; N, net-like. *n*=21 microscopic fields/group. Two-way repeated measured ANOVA, followed by Dunnett's multiple comparisons test, *F*_treatment_(1, 244)=0.01308, *P*=0.9090; *F*_morphology_(3, 244)=32.65, *P*<0.0001; *F*_interaction_(3, 244)=54.58, *P*<0.0001. (C) Immunofluorescence images showing the mitochondrial morphology in the *Dnm1l*^+/+^ and *Dnm1l*^+/Py^ cells transfected with the GTPBP8–mCherry construct for 48 h. Scale bar: 10 μm (cell images); 1 μm (magnifications). (D) Scoring of mitochondrial morphologies for GTPBP8–mCherry-overexpressing *Dnm1l*^+/+^ and *Dnm1l*^+/Py^ cells. Mitochondria in each GTPBP8–mCherry-transfected cell were scored into one of four morphological categories. *n*=12 microscopic fields/group. Two-way repeated measured ANOVA, followed by Dunnett's multiple comparisons test, *F*_treatment_(1, 88)=0.9621, *P*=0.3293; *F*_morphology_(3, 88)=18.13, *P*<0.0001; *F*_interaction_(3, 88)=63.65, *P*<0.0001. All error bars are mean±s.e.m. **P<0.01; ***P<0.001 compared to control.

To investigate whether GTPBP8 expression-mediated mitochondrial fission is associated with the regulation of Drp1 activity, we expressed GTPBP8–GFP in U2OS cells after silencing Drp1 with siRNA (Fig. S5A). When compared to the fragmented mitochondrial morphology observed in GTPBP8–GFP-expressed control cells, the expression of GTPBP8–GFP in Drp1 knockdown cells failed to induce fragmented mitochondrial morphology. After transiently transfecting GTPBP8–GFP into Drp1 knockdown U2OS cells for 48 h, the mitochondria in the transfected cells exhibited a tubular-like morphology similar to the surrounding non-transfected cells with deficient Drp1 (Fig. 4A; Fig. S5B). Mitochondria in U2OS cells expressing GTPBP8–GFP, both in control and Drp1 siRNA-treated conditions, were subsequently categorized based on their morphology. In line with the GTPBP8–GFP-expressing U2OS cells, the control siRNA-treated cells expressing GTPBP8–GFP predominantly showed fragmented mitochondria (denoted F). However, the proportion of cells with more than 50% of long tubular-like mitochondria (>50) and net-like mitochondria (denoted N) notably increased in Drp1-deficient cells expressing GTPBP8–GFP (Fig. 4B). These findings suggest that GTPBP8-mediated mitochondrial fission is dependent on Drp1 activity. Furthermore, GTPBP8–mCherry was expressed in primary mouse embryonic fibroblasts (MEFs) isolated from mice with a heterozygous mutation (C452F; +/Py) of the dynamin-1-like (*Dnm1l*) gene, encoding for Drp1, which results in mitochondrial hyperfusion. After transfecting GTPBP8–mCherry in MEF *Dnm1l*^+/Py^ cells for 48 h, the majority of cells continued to exhibit net-like mitochondrial morphology (Fig. 4C,D). These findings further reinforce the notion that defects in Drp1 block GTPBP8 expression-induced mitochondrial fragmentation. Collectively, these data indicate that GTPBP8-mediated mitochondrial fission is dependent on Drp1.

### GTPBP8 modulates the phosphorylation and recruitment of Drp1

**Fig. 5.**
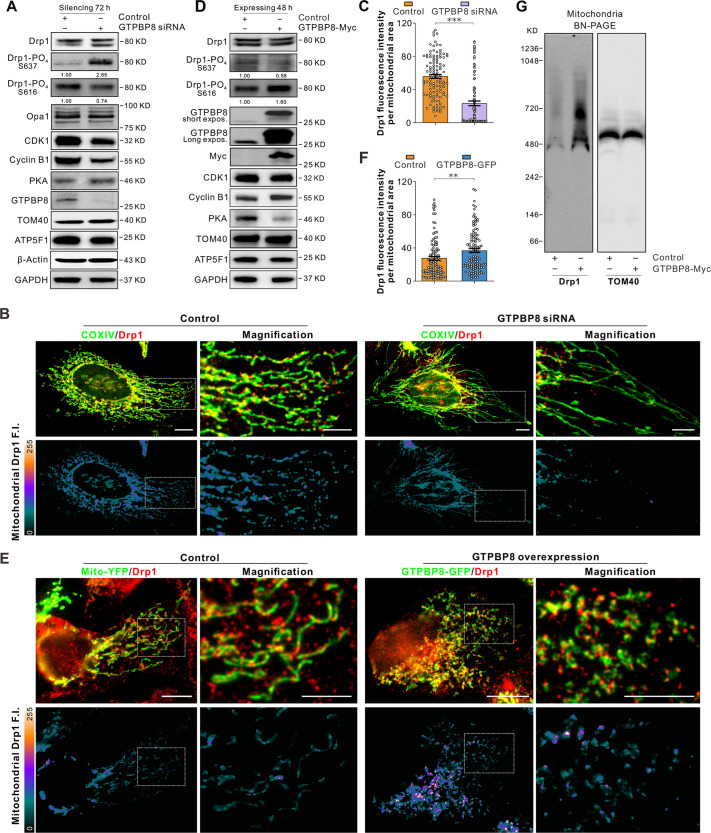
**GTPBP8 modulates the recruitment and phosphorylation of Drp1.** (A) Western blot of cell lysates after transfection with control and GTPBP8 siRNA 4# for 72 h. TOM40, β-actin, ATP5F1 and GAPDH are loading controls. (B) Immunofluorescence images showing Drp1 puncta in the control and GTPBP8 knockdown cells. To improve visualization of mitochondrial Drp1, cells were briefly treated with 0.001% digitonin before fixation to reduce the level of cytosolic Drp1. Mitochondria are highlighted by immunofluorescence against COXIV. Scale bars: 10 μm (cell images); 5 μm (magnifications). Lower panel, a mask corresponding to the mitochondrial channel was applied to the Drp1 channel to obtain only mitochondrial Drp1 fluorescence. Heatmap reflects mitochondrial-distributed Drp1 fluorescence intensity (F.I.). (C) Bar graph showing the fluorescence intensity of mitochondria-distributed Drp1 puncta. Control, *n*=111 mitochondrial areas; GTPBP8 siRNA, *n*=110 mitochondrial areas. Unpaired two-tailed *t*-test, t(219)=8.831, *P*<0.0001. (D) Western blot of cell lysates after transient transfection with GTPBP8-Myc for 48 h. TOM40, ATP5F1 and GAPDH are loading controls. (E) Immunofluorescence images show Drp1 puncta in the Mito–YFP- and GTPBP8–GFP-overexpressing cells. To improve visualization of mitochondrial Drp1, cells were briefly treated with 0.001% digitonin before fixation to reduce the level of cytosolic Drp1. Scale bars: 10 μm (cell images); 5 μm (magnifications). Lower panel, a mask corresponding to the mitochondrial channel was applied to the Drp1 channel to obtain only mitochondrial Drp1 fluorescence. Heatmap reflects mitochondrial-distributed Drp1 F.I. (F) Bar graph showing the fluorescence intensity of mitochondria-distributed Drp1 puncta. *n*=111 mitochondrial areas/group. Unpaired two-tailed *t*-test, t(220)=2.881, *P*=0.0044. (G) Effects of GTPBP8–Myc overexpression on the oligomeric state of Drp1 on the mitochondria. TOM40 is loading control. Images shown in A, B and D representative of three repeats. All error bars are mean±s.e.m. ***P<0.001 compared to control.

Mitochondrial fission is dependent on Drp1, which assembles into helical oligomers, enveloping the pre-constriction site to facilitate membrane constriction (Wang et al., 2022). To investigate whether the decreased mitochondrial fission upon GTPBP8 depletion was correlated with Drp1 abundance, the expression level of Drp1 was assessed. In contrast to control cells, GTPBP8 knockdown resulted in a slight elevation of Drp1 levels, mainly in the lower band (Fig. 5A). Conversely, the depletion of GTPBP8 had no noticeable effect on the levels of long Opa1 (L-Opa1) and short Opa1 (S-Opa1), which are implicated in both mitochondrial fission and fusion (Ishihara et al., 2006; Wang et al., 2022). This observation suggests that the influence of GTPBP8 depletion on the abundance of Drp1 is distinct and specific. Drp1 activity is often regulated by different post-translational modifications, such as phosphorylation (Figueroa-Romero et al., 2009; Xu et al., 2016). Phosphorylation of Drp1 at Ser616 (Drp1 S616-PO_4_) facilitates mitochondrial fission, whereas phosphorylation at Ser637 (Drp1 S637-PO**_4_**) inhibits mitochondrial fission. The knockdown of GTPBP8 had a remarkable impact on the phosphorylation of Drp1. Specifically, there was a distinct increase in the phosphorylation of Drp1 at S637 (Drp1 S637-PO_4_), whereas the phosphorylation of Drp1 at S616 (Drp1 S616-PO_4_) showed a relatively lesser effect upon depletion of GTPBP8 (Fig. 5A). The elevated level of the lower band of Drp1 could be primarily attributed to the increased levels of Drp1 S637-PO_4_. It is worth mentioning that phosphorylation of Drp1 at S616 has been associated with promoting mitochondrial fission (Taguchi et al., 2007), whereas phosphorylation of Drp1 at S637 has been shown to hinder its translocation to mitochondria and inhibit mitochondrial fission (Chang and Blackstone, 2007; Ko et al., 2016). The increased phosphorylation of Drp1 at S637 resulting from GTPBP8 depletion indicates an inhibition of mitochondrial fission.

Phosphorylation of Drp1 can be modulated by different protein kinases involved in distinct signaling pathways, such as CDK1–cyclin B, ROCKs (ROCK1 and ROCK2) and PKA (Kashatus et al., 2011; Liesa et al., 2009; Taguchi et al., 2007; Wang et al., 2012). To investigate whether changes in the phosphorylation of Drp1 were regulated by those protein kinases, we detected the expression levels of CDK1, cyclin B, ROCK1, ERK1/2 (also known as MAPK3 and MAPK1, respectively) and PKA. Compared to control cells, GTPBP8 knockdown resulted in a reduced CDK1 and cyclin B level but an increased PKA level. The expression levels of ROCK1 and ERK1/2 were unaffected following the depletion of GTPBP8 (Fig. 5A; Fig. S5C). These data indicate that the low energy state and increased oxidative stress resulting from GTPBP8 depletion might lead to the upregulation of PKA and the downregulation of CDK1–cyclin B. Consequently, those altered cell signaling pathways would contribute to the increased phosphorylation of Drp1 at Ser637. This, in turn, results in reduced recruitment of Drp1 to mitochondria, ultimately leading to the observed elongated mitochondrial morphology.

To determine whether the upregulated Drp1 S637-PO_4_ level in GTPBP8 knockdown cells induces the deficiency in recruiting Drp1 to mitochondria, we next used immunostaining to visualize Drp1 puncta on mitochondria in cells. Cells were pre-treated with 0.001% digitonin before fixation to reduce the level of cytosolic Drp1 and thus improve visualization of mitochondrial Drp1. The immunofluorescence images show that a proportion of Drp1 in control cells formed punctate structures on mitochondria, whereas in GTPBP8-depleted cells, this recruitment to mitochondria was reduced (Fig. 5B). This observation was further supported by quantitative analysis, showing a lower fluorescence intensity per mitochondrial area in the GTPBP8-depleted cells compared to in the control cells (Fig. 5C). These data indicate that GTPBP8 deficiency leads to a reduction in the recruitment and assembly of Drp1 to mitochondria. The increased phosphorylation of Drp1 at S637 following GTPBP8 depletion impedes the recruitment of Drp1 to the mitochondrial outer membrane, ultimately contributing to the reduced mitochondrial fission.

The impact of GTPBP8 on the recruitment and assembly of GTPBP8 was further examined in GTPBP8-overexpressing cells. GTPBP8–Myc expression caused a reduction in the phosphorylation of Drp1 at S637 and the level of PKA, accompanied by an increase in the phosphorylation of Drp1 at S616 and the level of cyclin B (Fig. 5D). These changes suggest an enhanced translocation of Drp1 to mitochondria in GTPBP8-overexpressing cells. Moreover, in control cells expressing Mito–YFP, a plasmid targeting the mitochondrial matrix, Drp1 staining was mostly diffuse in the cytosol, although a portion in punctate structures was observed on mitochondria. In contrast, cells with overexpression of GTPBP8–GFP exhibited an increased number of mitochondrial Drp1 puncta, along with reduced cytosolic distribution of Drp1, indicating that expression of GTPBP8–GFP enhanced recruitment of Drp1 to mitochondria (Fig. 5E). In contrast to what was seen for GTPBP8-depleted cells, the quantification of Drp1 intensity per mitochondrial area showed that GTPBP8 overexpression in U2OS cells promoted the recruitment of Drp1 to mitochondria (Fig. 5F).

Following recruitment to the mitochondrial outer membrane, Drp1 wraps around and constricts mitochondrial tubules by self-assembling them into a spiral superstructure to promote the scission event (Jimah and Hinshaw, 2019; Yu et al., 2021). To examine whether GTPBP8 expression affects the self-assembly of Drp1 on mitochondria, we isolated mitochondria and assessed the oligomerization state of Drp1 using Blue Native-PAGE (BN-PAGE). In comparison to control cells, GTPBP8–Myc-expressing cells exhibited a noticeable increase in oligomerized Drp1 (with a molecular mass of ∼480 kDa or larger) (Fig. 5G). Taken together, these findings demonstrate that GTPBP8 mediates mitochondrial fission by influencing the recruitment, assembly and phosphorylation of Drp1.

## DISCUSSION

Mitochondrial fission is a fundamental cellular process that plays a crucial role in maintaining the dynamic and functional properties of mitochondria. It involves the division of a single mitochondrion into two separate daughter organelles, each with its own inner and outer membranes. This process is essential for various cellular functions, including energy production, Ca^2+^ homeostasis, apoptosis and quality control of mitochondria. The primary regulator of mitochondrial fission is Drp1, a cytosolic GTPase that is recruited to the mitochondrial outer membrane at specific sites known as fission sites. Drp1 forms helical or ring-like oligomers around the mitochondrial tubules, constricting the membrane and leading to scission. Although the process of mitochondrial fission has been extensively studied, there are still many aspects that remain to be fully elucidated. For instance, the involvement of inner membrane proteins, particularly those on the matrix side of the inner membrane, in the fission process is less known. Additionally, the precise coordination between outer and inner membrane fission events and the factors involved are yet to be defined.

We recently reported that GTPBP8 localizes to the mitochondrial inner membrane and functions in the assembly of the mitoribosomal large subunit (mt-LSU) (Wang et al., 2023). In this present study, we report on the role of GTPBP8 in modulating mitochondrial fission. Apart from the impaired assembly of mt-LSU and the consequent compromised mitochondrial translation previously seen (Wang et al., 2023), we here also observed a pronounced alteration in mitochondrial morphology following GTPBP8 depletion, characterized by a strikingly elongated and interconnected appearance. Further exploration into the proteins involved in mitochondrial dynamics illuminated an intriguing observation: an elevation in the levels of Drp1 phosphorylated at S637 (Drp1 S637-PO_4_). This post-translational modification was anticipated to hinder the translocation of Drp1 from the cytosol to the outer membrane of the mitochondria, ultimately resulting in the observed hyperfused mitochondrial morphology. In contrast, the GTPBP8 overexpression yielded a contrasting effect, inducing a shift in mitochondrial shape from tubular to fragmented. However, this GTPBP8-induced mitochondrial fragmentation was notably hampered in cells either deficient in Drp1 or carrying mutated Drp1, underscoring the indispensable role of Drp1 in mediating the impact of GTPBP8 on mitochondrial fission.

GTPBPs constitute a subset of the expansive P-loop NTPase superfamily, engaging in an array of intricate cellular processes encompassing transport, cytoskeleton organization, translation, cellular differentiation and proliferation (Leipe et al., 2002; Lo et al., 2022; Maiti et al., 2020; Zinoviev et al., 2018). Notably, several GTPBPs have homologous counterparts in bacterial systems, where they serve analogous functions within mammalian cells. Among these GTPBP family members, GTPBP8 emerges as a homolog to YihA in *E. coli* and YsxC in *B. subtilis*, which play important roles in ribosomal assembly (Gupta et al., 2020; Ruzheinikov et al., 2004). Strikingly, our investigation aligns with this context, as we have previously unearthed a pivotal role for GTPBP8 in the assembly of the mitoribosome, particularly regarding the role of GTPBP8 in the maturation of mt-LSU; depletion of GTPBP8 was previously shown to impair the assembly of mt-LSU, ultimately reducing mitochondrial respiration and bioenergy production (Wang et al., 2023).

Mitochondria are highly dynamic and undergo fission and fusion to adapt to multiple cell activities. Under certain conditions, such as mtDNA loss or mitochondrial membrane depolarization, excessive fission occurs. Conversely, hyperfused or elongated mitochondria are visualized under mild starvation (Quintana-Cabrera and Scorrano, 2023). To preserve normal mitochondrial function in a low-energy state, mitochondria in GTPBP8-depleted cells are expected to fuse together, forming hyperfused mitochondrial morphologies. However, in fact, hyperfused mitochondrial morphology is not consistently observed in cells with a low-energy state caused by the depletion of other GTPBPs. Through examining mitochondrial morphology after depleting three other mitoribosomal assembly factors, GTPBP5 (Maiti et al., 2020), GTPBP7 (Kotani et al., 2013) and GTPBP10 (Lavdovskaia et al., 2018), we found that not all cells in a low-energy state presented elongated or interconnected mitochondrial morphology. These findings indicate that the role of GTPBP8 in mediating mitochondrial fission is specific.

GTPBP8 is located in the mitochondrial matrix and inner membrane (Wang et al., 2023), whereas Drp1 resides in the cytosol. Our data demonstrate that the mitochondrial translation defects in GTPBP8-depleted cells led to increased oxidative stress and, consequently, impaired mitochondrial function. This mitochondrial dysfunction might further facilitate modified phosphorylation of Drp1 at S637 by upregulating PKA and downregulating CDK1–cyclin B. Importantly, the role of GTPBP8 in regulating Drp1 activity is closely linked to the phosphorylation status of Drp1 at specific serine residues. Phosphorylation at Drp1 Ser616 promotes its assembly and constriction around the mitochondrial tubules, initiating the fission process (Taguchi et al., 2007). Conversely, phosphorylation at Drp1 Ser637 inhibits its translocation to the mitochondrial outer membrane, thereby reducing fission (Chang and Blackstone, 2007). The increased level of phosphorylation of Drp1 at S637 resulting from GTPBP8 depletion leads to an inhibition of Drp1 recruitment to mitochondria. Consequently, the reduced presence of Drp1 at the mitochondrial outer membrane hampers the initiation of fission events. This disruption in the balance between fission and fusion processes shifts the equilibrium towards fusion, leading to the elongated and interconnected mitochondria characteristic of a hyperfused state.

We show that GTPBP8-mediated mitochondrial fission relies on the recruitment and assembly of Drp1 at the mitochondrial outer membrane, thus promoting the scission event. Given that GTPBP8 is localized within the mitochondrial matrix and inner membrane rather than the outer membrane, where Drp1 receptors are typically found, its role in regulating mitochondrial fission might involve different mechanisms. First, localization of GTPBP8 to the inner mitochondrial membrane suggests that it could have a role in inner membrane dynamics. It might participate in processes related to inner membrane curvature or remodeling, which in turn could impact the overall fission process. Second, GTPBP8 could interact with other proteins involved in mitochondrial fission, either directly or indirectly, to modulate the activity of Drp1. This could affect the localization, activation, or assembly of Drp1 at the fission sites, as we demonstrated in this study. And third, considering the reported role for GTPBP8 in mitochondrial ribosome assembly, it is possible that its influence on mitochondrial translation and protein synthesis indirectly impacts the machinery required for mitochondrial fission. Notably, these possibilities are not mutually exclusive. Further studies are required to uncover the specific molecular interactions and pathways through which GTPBP8 modulates mitochondrial dynamics and fission, especially considering its unique localization within the mitochondria.

In conclusion, GTPBP8 emerges as a pivotal modulator of mitochondrial fission through a Drp1-dependent process. The multifaceted functions of GTPBP8, encompassing its potential influence on Drp1 phosphorylation and recruitment, actively contribute to the assembly of Drp1 on the outer mitochondrial membrane. This orchestrated interplay between GTPBP8 and Drp1 plays a crucial role in governing mitochondrial dynamics, ultimately shaping the overall structure and function of mitochondria.

## MATERIALS AND METHODS

### Reagents and antibodies

Valinomycin (94675-10MG, Sigma) was used at a final concentration of 10 µM. Carbonyl cyanide *m*-chlorophenylhydrazone (CCCP, 215911-250MG, Sigma) was used at a final concentration of 10 µM. Digitonin (D141, Sigma) was used at a final concentration of 0.001%. All antibodies used in this study are listed in Table S1.

### Plasmids

Human *GTPBP8* cDNA (ORFeome Library; Biocentrum Helsinki Genome Biology Unit) was separately cloned into the pEGFP-N1 vector and mCherry-N1 vector (Takara Bio Inc.) using the XhoI/BamHl cloning sites. GTPBP8–Myc was constructed from the GTPBP8–GFP plasmid with GFP replaced by Myc (Wang et al., 2023). Mito–YFP and Mito–DsRed were gifts from Dr Elena Kremneva (Institute of Life Science HiLIFE, University of Helsinki, Finland).

### Cell culture

Human osteosarcoma (U2OS) cells (authenticated by ECACC through STR-profiling method to be the same origin as the original U2OS cell line; case number-13472), human ovarian cancer SKOV3 cells (ATCC) and human embryonic kidney HEK293 cells (ATCC) were cultured in Dulbecco's modified Eagle's medium (DMEM; high glucose, C1995500BT, Gibco) containing 10% fetal bovine serum (FBS), 100 U/ml penicillin, 100 µg/ml streptomycin and 2 mM L-glutamine (25030-081, Gibco). All cell lines were routinely tested negative for mycoplasma with a MycoAlert^®^ PLUS Mycoplasma Detection Kit (LT07-703, Lonza).

Mouse embryonic fibroblasts (MEFs) were a kind gift from Brendan J. Battersby (Institute of Biotechnology, University of Helsinki, Finland) and were isolated from *Dnm1l*^+/+^ and *Dnm1l*^+/Py^ embryos. MEF cells were also cultured in DMEM with high glucose supplemented with 10% FBS, 2 mM L-glutamine and 50 mg/ml uridine. U2OS cells and MEF cells were cultured at 37°C in an incubator with humidified 95% air and 5% CO_2_.

### Cell transfection and treatment

For transient expression of DNA constructs, cells were plated on culture plates and transfected the next day with the indicated DNA constructs using FuGene HD transfection reagent (E2311, Promega) according to the manufacturer's instructions, using a ratio between reagent and plasmid DNA of 3.5:1. After incubation for 48 h, cells were subsequently fixed with 4% PFA or replated on 10 μg/ml fibronectin-coated (11051407001, Roche) glass-bottomed dishes (MatTek) for live cell imaging.

U2OS cells were transfected with 30 pmol of GTPBP8, Drp1, or control scramble siRNA using Lipofectamine RNAiMAX transfection reagent (#13778-075, Thermo Fisher Scientific) according to the manufacturer's instructions. The sequence information of the targeted siRNA is as follows: GTPBP5 siRNA, 5′-CGGUGGACACGUCAUUCUGTT-3′ (134621, Ambion); GTPBP7 siRNA, 5′- GCAACACUUAGAAGGAGAAGGCCUA-3′ (1362318, Invitrogen); GTPBP8 siRNA 2#, 5′-AGCGACTGAGCCGCTATAATA-3′ (SI04232011, Qiagen); GTPBP8 siRNA 4#, 5′-CCGGTTTAGCTGAAGATTCAA-3′ (SI00443779, Qiagen); GTPBP10 siRNA 5′-TTGCGTGTTGTTCAGAAAGTA-3′ (SI04308647, Qiagen); Drp1 siRNA as previously used (Lee et al., 2004), 5′-TTCAATCCGTGATGAGTATGCTTTTCTTC-3′ (RIBO, China); and control scramble siRNA (SI03650318, Qiagen).

### Immunoblotting

Cells were collected by scraping and solubilized with ice-cold PBS containing 1% N-dodecyl-β-d-maltopyranoside (DDM, D4641, Sigma), 1 mM PMSF, 10 mM sodium azide and 10 mM sodium ascorbate. Protein concentrations were assessed by the Bradford assay (Bio-Rad Laboratories). Equal amounts of proteins were separated by Tris-glycine SDS-PAGE and transferred to the polyvinylidene fluoride (PVDF) membrane (ISEQ00010, Millipore). Primary antibodies were incubated with the membrane overnight at 4°C and detected the following day with HRP-linked secondary antibodies using an ECL reagent (WBKLS0500, Millipore). Full uncropped images of blots from this paper are available in Fig. S6.

### Immunofluorescence

U2OS cells grown on coverslips were fixed with 4% paraformaldehyde for 20 min, followed by two washings with PBS. Cells were sequentially permeabilized with 0.1% Triton X-100 in PBS for 5 min, blocked with 0.2% BSA for 30 min, and incubated with primary antibody overnight at 4°C. After three washes with PBS, cells were incubated with fluorescence-conjugated secondary antibodies for 1 h at RT. Finally, coverslips were mounted in Mowiol supplemented with DABCO. Images were acquired by a laser scanning confocal microscope (Leica SP8 TCS) with built-in LAS X software (Leica Biosystem) or a DM6000B microscope (Leica Biosystems) equipped with a Hamamatsu Orca-Flash 4.0 V2 sCMOS camera and LAS-X software (Leica Microsystems) using a 63×1.4-0.60 HCX Plan Apo chromat objective and the Semrock BrightLine filters GFP-4050B (excitation, 466/40 nm; emission, 525/50 nm) and TRITC-B (excitation, 543/22 nm; emission, 593/40 nm). All images were processed and analyzed using Image J (https://imagej.net/ij/index.html).

For analysis of mitochondria-distributed Drp1, images in both experimental groups were obtained with the same settings. Images were analyzed using Image J. The intensity of mitochondrial-distributed Drp1 per mitochondrial area was quantified from 0.1–3 µm line scans intersecting mitochondria.

### Scoring of mitochondrial network morphology

Mitochondrial network morphology was manually scored by a researcher unaware of the treatment, as previously reported (Losón et al., 2013). For GTPBP8 deficiency-caused interconnected mitochondrial morphology, the mitochondrial morphology of each cell was scored into one of four categories: short, more than 50% of mitochondria in a single cell exhibited short tubules; long, more than 50% of mitochondria in a single cell exhibited long tubules; net-like, most of the mitochondria in a single cell were interconnected with few or no long tubules; and collapsed, most of the mitochondria are highly condensed and accumulated around the nucleus with few or no individual mitochondria around the cell membrane. For GTPBP8 expression-induced mitochondrial fragmentation, the mitochondrial morphology of each cell was also manually scored into one of four categories: fragmented (F), almost all mitochondria exhibited dot-like morphology; <50, less than 50% of mitochondria in a single cell exhibited long tubules; >50, more than 50% of mitochondria in a single cell exhibit long tubules; and net-like, most of the mitochondria in a single cell were interconnected with few or no long tubules. The ratio of each kind of mitochondrial morphology was generated by dividing the total number of cells in one mitochondrial category by the total number of cells in the microscopic field.

### Live-cell imaging

U2OS cells were transiently transfected with siRNA and Mito–DsRed, a plasmid encoding red fluorescent protein fused to a mitochondrial targeting peptide from subunit VIII of human cytochrome *c* oxidase (MSVLTPLLLRGLTGSARRLPVPRAKIHSLPP), prior to 72 h and 48 h, respectively. Cells were then replated on 10 μg/ml fibronectin-coated glass-bottomed dishes (MatTek). The dish was placed in a heated sample chamber with 5% CO_2_. The time-lapse images were acquired with a Marianas imaging system (3I) equipped with an inverted spinning-disk confocal microscope (Axio-Observer Z1; Zeiss) and a Yokogawa CSU-X1 M1 confocal scanner. A 63$ 1.2 W C-Apochromat Corr working distance=0.28 M27 objective was used, and all the images were acquired by an sCMOS (Andor) Neo camera and Slidebook 5.0 software (3I) or a laser scanning confocal microscope (Leica SP8 TCS) with built-in LAS X software (Leica Biosystem). Quantification of fission and fusion events was manually performed in the ImageJ.

### Transmission electron microscopy

Transmission electron microscopy was performed as previously described (Wang et al., 2019). Briefly, after treatment with the siRNA for 72 h or overexpression of GTPBP8–GFP for 48 h, cells were sequentially fixed, osmicated, dehydrated in a graded ethanol series and acetone, and infiltrated gradually with Epon (TAAB). Next, 60-nm-thick sections were sliced parallel to the coverslip and poststained with uranyl acetate and lead citrate. Specimens were visualized, and images were acquired using a Tecnai 12 (FEI) microscope equipped with an Orius SC 1000B bottom-mounted charge-coupled device camera (Gatan) at an accelerating voltage of 80 kV.

### Measurement of mitochondrial membrane potential

Mitochondrial membrane potential (ΔΨm) was measured using the fluorescent dye tetramethylrhodamine, methyl ester (TMRM; T668; Thermo Fisher Scientific) as previously described (Wang et al., 2019). After treatment with control or GTPBP8 siRNA in U2OS cells for 72 h, cells were harvested, washed and re-suspended in DMEM without the Phenol Red indicator. Cells were subsequently stained with 50 nM TMRM for 15 min at 37°C. The TMRM fluorescence was measured with the LSRFortessa flow cytometer (BD Biosciences), excited with a 488 nm laser, and recorded with a 575/26 nm PE-detector. The TMRM fluorescence intensity was normalized to mitochondrial mass, detected with MitoTracker green FM (M7514, Thermo Fisher Scientific), a mitochondrial marker regardless of mitochondrial membrane potential. Flow cytometric analysis was performed using FlowJo software (FlowJo LLC, Ashland, OR, USA).

### Detection of cellular ROS

Cellular superoxide anions were measured using dihydroethidium (D1168; Thermo Fisher Scientific) as previously described (Wang et al., 2019). After treatment with control or GTPBP8 siRNA in U2OS cells for 72 h, cells were harvested and stained with 10 μM DHE dissolved in pre-warmed PBS for 15min in the dark. The DHE fluorescence was measured by the LSRFortessa flow cytometer (BD Biosciences), excited with a 488 nm laser, and recorded with a 575/26 nm PE-detector. The total mitochondrial mass was evaluated by staining the cells with MitoTracker Green FM (M7514; Molecular Probes). The changes in fluorescence intensity of DHE were normalized to mitochondrial mass. Flow cytometric analysis was performed using FlowJo software (FlowJo LLC).

### Mitochondrial isolation

Mitochondria were extracted from the mammalian cells, as previously described (Frezza et al., 2007). Briefly, cells were washed in pre-cold PBS and resuspended in homogenization buffer (10 mM Tris-MOPS, 1 mM EGTA and 200 mM sucrose, pH 7.4) after overexpression of GTPBP8–Myc for 48 h. The cell suspension was subsequently homogenized with a rotating Teflon potter (Potter S; Braun). The non-lysed cells were sedimented by centrifuging twice at 600 ***g*** for 10 min and discarded. The supernatant was then centrifuged at 7000 ***g*** for 10 min, and the resulting pellet was the crude mitochondria.

### Blue-native PAGE

The blue-native PAGE (BN-PAGE) was performed using the NativePAGE Novex Bis-Tris gel system (BN1001BOX; Invitrogen) according to the manufacturer's protocol. Briefly, the crude mitochondria were lyzed in 1% DDM containing 1 mM PMSF, 10 mM sodium azide and 10 mM sodium ascorbate. After lyzing on ice for 30 min, the cell lysates were centrifuged at 22,000 ***g*** for 30 min. The supernatant was collected and added with Coomassie Blue G250 dye, with a final concentration of 0.2%. Equal amounts of proteins were separated by a 3–12% gradient NativePAGE gel. Proteins were transferred to the PVDF membrane (EMD Millipore). The membrane was then incubated in 8% acetic acid, activated with methanol and blocked with non-fat milk. The diluent primary antibody against Drp1 was incubated overnight at 4°C and detected the following day with an HRP-linked secondary antibody using an ECL reagent.

### Statistical analysis

All investigators were unaware of experimental status during the acquisition and analysis of data. Unless otherwise noted, statistical analysis was performed using an unpaired Student's *t*-test (two-tailed), and a two-way ANOVA with GraphPad Prism 9 software (version 9). *P*<0.05 was chosen as the minimum level of significance. Data are presented as mean±s.e.m. The number of mitochondria and the microscopic fields that were used for the statistical analysis are presented in the legend of the corresponding figure.

## Supplementary Material



10.1242/joces.261612_sup1 Supplementary information

Table S1. Antibodies used in this study.

## Data Availability

All relevant data can be found within the article and its supplementary information.

## First Person

This article has an associated First Person interview with Xiumei He, joint first author of the paper.
