# Supplementary material for: GTPBP8 modulates mitochondrial fission through a Drp1-dependent process
Source: J Cell Sci. 2024 Apr 30;137(8):jcs261612. doi: 10.1242/jcs.261612 (PMC11112121; doi:10.1242/jcs.261612)
Supplement: Supplementary information [file joces-137-261612-s1.pdf]

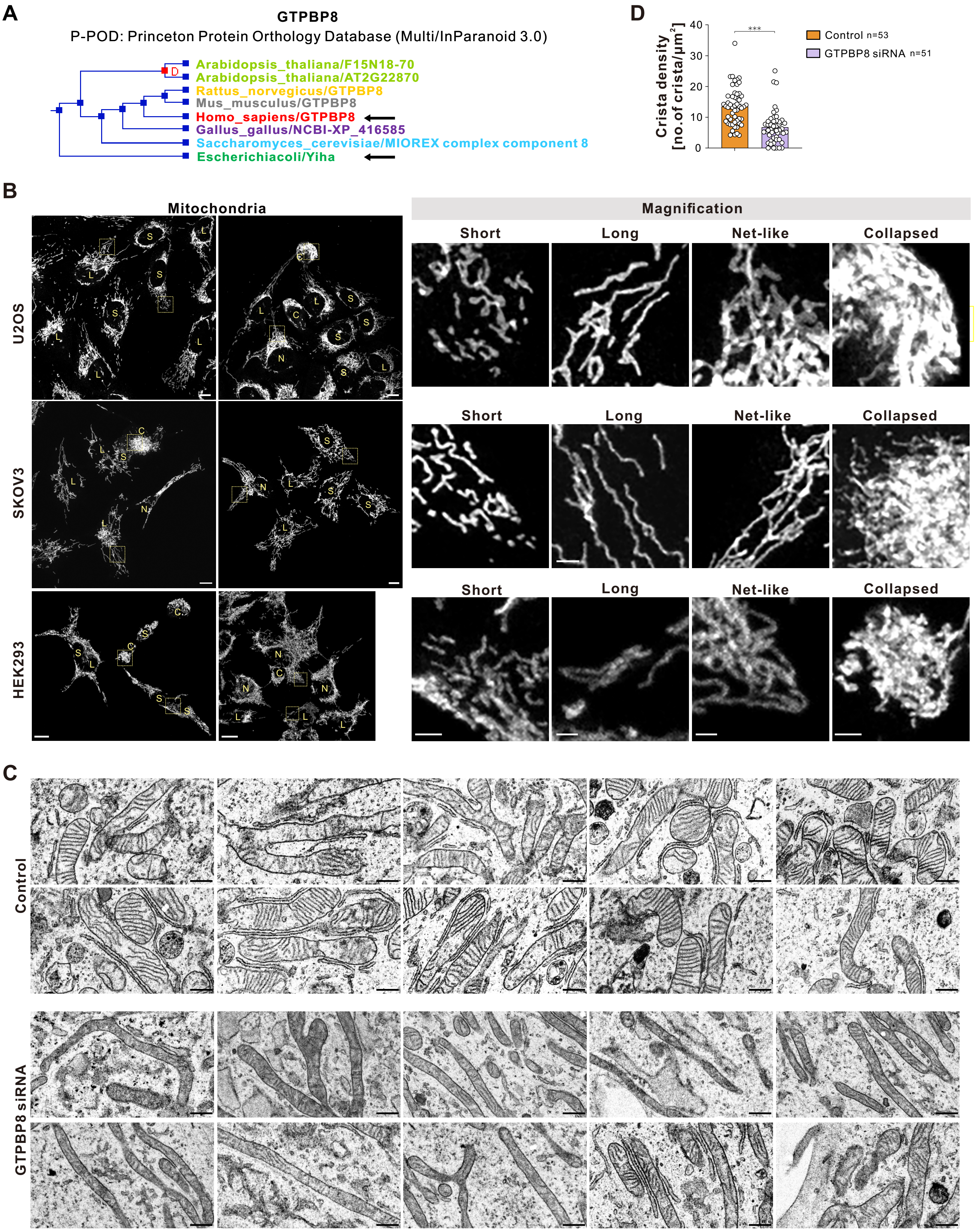

Journal of Cell Science • Supplementary information

Fig. S1. Knockdown of the P-loop_NTPase superfamily protein GTPBP8 causes mitochondrial elongation.

(A) Ortholog analysis of GTPBP8 obtained with the Princeton Protein Orthology Database (P- POD). An Ortholog identification (Multi/InParanoid 3.0) graph is presented. The evolutionarily conserved human GTPBP8 and *E.coli* Yiha were highlighted with the black arrows. **(B)** The representative images show scoring of mitochondrial network morphology in U2OS, SKOV3, and HEK293 cells, respectively. The mitochondrial morphology of each cell in the left panel is scored into one of four categories: short (S), long (L), net-like (N), and collapsed (C). The representative images showing each kind of mitochondrial morphology are magnified in the right panel. Mitochondria were visualized by probing for TOM20. Scale bar in the left panel, 10 µm. Scale bar in magnified images, 2 µm. **(C)** Electron micrographs of mitochondria in U2OS cells treated with control and GTPBP8 siRNA for 72 h. Scale bar, 500 nm. **(D)** Bar graph shows the total number of cristae per mitochondrial area (μm^2) using TEM images. Control, n = 53 mitochondria; GTPBP8 siRNA, n = 51 mitochondria. Unpaired two-tailed t-test, $t(102) = 6.057$, $P < 0.0001$.

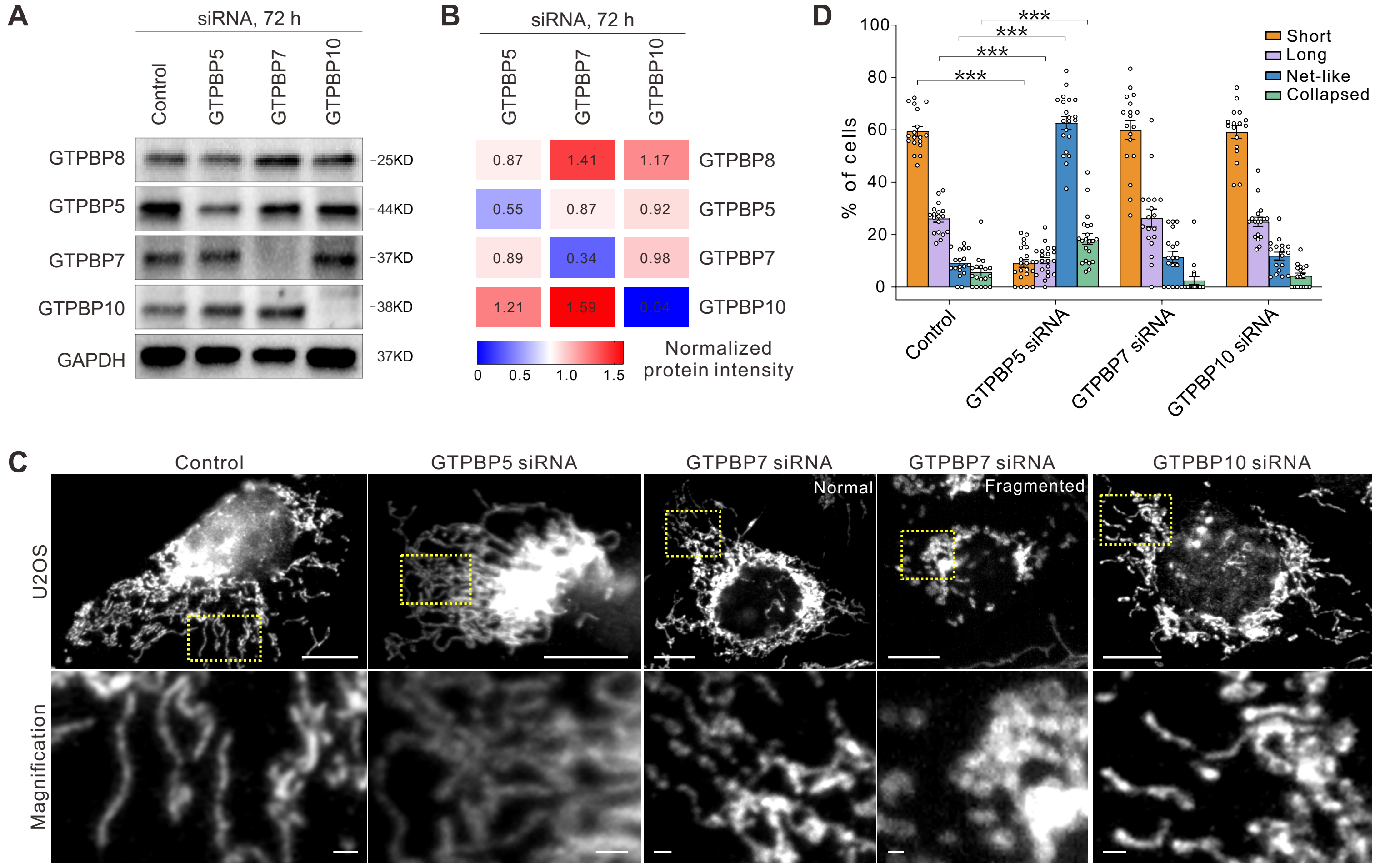

Fig. S2. Changes in mitochondrial morphology after silencing the other three assembly proteins of the mitoribosomal large subunit (mtLSU).

(A) Changes in steady-state GTPase levels treated with indicated siRNA for 72 h. GAPDH is loading control. **(B)** Heatmap showing the normalized GTPase levels. The number on the heatmap represents the normalized protein value. The color gradient ranging from blue to red was used to colorize the normalized protein level. **(C)** The representative images show mitochondrial morphology in U2OS cells treated with indicated siRNA for 72 h.

Journal of Cell Science • Supplementary information

Mitochondria were visualized by immunofluorescence against TOM20. Scale bar in the upper panel, 10 µm. Scale bar in the lower panel, 1 µm. **(D)** Scoring of mitochondrial morphologies for control and GTPase knockdown cells. Mitochondria in each cell were scored into one of four morphological categories. Control, n = 18 microscopic fields; GTPBP5 siRNA, n = 21 microscopic fields; GTPBP7 siRNA, n = 18 microscopic fields; GTPBP10 siRNA, n = 17 microscopic fields. Two-way repeated measured ANOVA, followed by Dunnett's multiple comparisons test, $F_{\text{treatment}}(3, 280) = 1.084 \times 10^{-20}$, $P > 0.9999$; $F_{\text{morphology}}(3, 280) = 253.8$, $P < 0.0001$; $F_{\text{interaction}}(9, 280) = 124.8$, $P < 0.0001$;

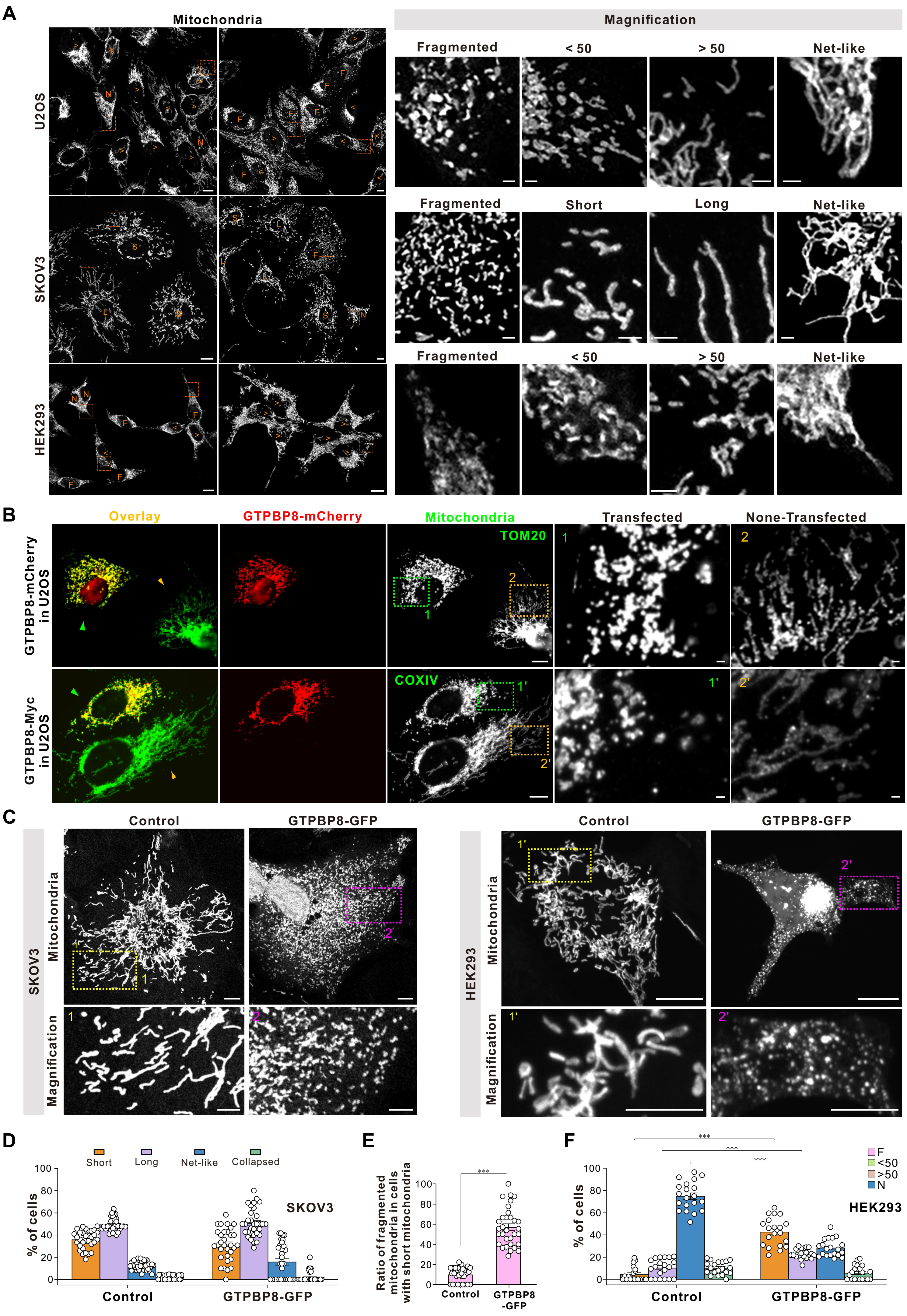

Journal of Cell Science • Supplementary information

Fig. S3. GTPBP8 expression promotes mitochondrial fission.

(A) The representative images show scoring of mitochondrial network morphology upon GTPBP8 overexpression in U2OS, SKOV3, and HEK293 cells, respectively. For U2OS, and HEK293, the mitochondrial morphology of each cell in the left panel is scored into one of four categories: Fragmented (S), <50% of mitochondria are short or long tubules (<), >50% of mitochondria are short or long tubule (>), and net-like (N). For SKOV3, due to the lower ratio of fragmented mitochondria, the mitochondrial morphology was thus scored into one of four categories: short (S), long (L), net-like (N), and collapsed (C). In particular, some cells in the short group presenting fragmented mitochondria were additionally counted. The representative images showing each kind of mitochondrial morphology are magnified in the right panel. Mitochondria were visualized by probing for TOM20. Scale bar in the left panel, 10 µm. Scale bar in magnified images, 2 µm. **(B)** Immunofluorescence images show the mitochondrial morphology in U2OS cells that were transiently transfected with the GTPBP8-mCherry or GTPBP8-Myc constructs for 48 h. Mitochondria were visualized by probing for TOM20 or COXIV. The non-transfected and transfected cells were highlighted with orange and green arrows, respectively. Scale bar in the left panel, 10 µm. Scale bar in magnified images, 1 µm. **(C)** Immunofluorescence images show the mitochondrial morphology in SKOV3 and HEK293 cells that were transiently transfected with the GTPBP8-GFP construct for 48 h. Mitochondria in control cells were visualized by overexpressing Mito-YFP. Scale bar in the upper panel, 10 µm. Scale bar in magnified images, 5 µm. **(D)** Scoring of mitochondrial morphologies for GTPBP8-GFP transfected and control SKOV3 cells. Control, n = 32 microscopic fields; GTPBP8-GFP, n = 32 microscopic fields. Two-way repeated measured ANOVA, followed by Dunnett's multiple comparisons test, $F_{treatment}(1, 248) = 1.038 \times 10^{-13,}$ $P > 0.9999$; $F_{morphology}(3, 248) = 292.8$, $P < 0.0001$; $F_{interaction}(3, 248) = 1.900$, $P = 0.1301$. **(E)** Bar graph shows the ratio of SKOV3 cells with fragmented mitochondria in short group upon GTPBP8-GFP overexpression for 48 h. Control, n = 32 mitochondria; GTPBP8 siRNA, n = 32 mitochondria. Unpaired two-tailed t-test, $t(62) = 13.19$, $P < 0.0001$. **(F)** Scoring of mitochondrial morphologies for GTPBP8-GFP transfected and control HEK293 cells. Control, n = 20 microscopic fields; GTPBP8-GFP, n = 20 microscopic fields. Two-way repeated measured ANOVA, followed by Dunnett's multiple comparisons test, $F_{treatment}(1, 152) = 3.06 \times 10^{-12,}$ $P > 0.9999$; $F_{morphology}(3, 152) = 193.9$, $P < 0.0001$; $F_{interaction}(3, 152) = 172.7$, $P < 0.0001$.

Journal of Cell Science • Supplementary information

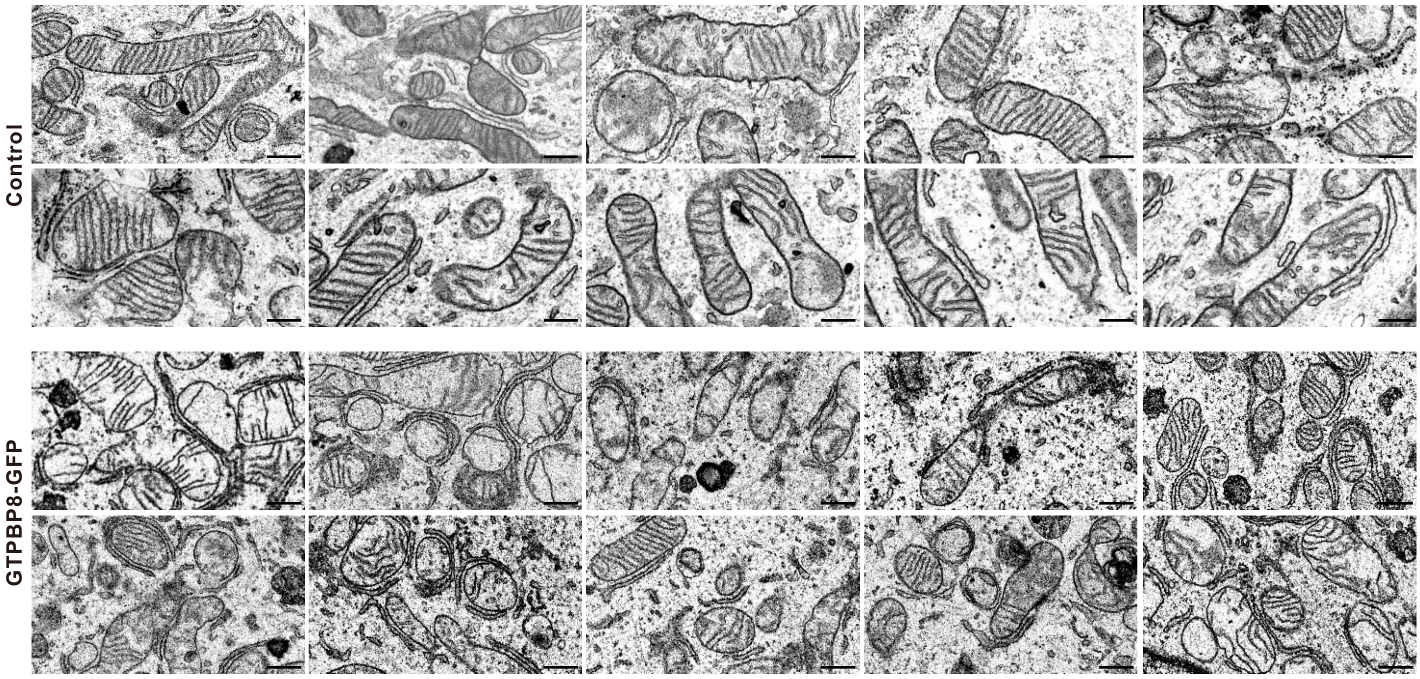

Fig. S4. GTPBP8 expression promotes mitochondrial fission with less affection on mitochondrial architecture.

Electron micrographs of mitochondria in U2OS cells transfected with control and GTPBP8-GFP for 48 h. Scale bar, 500 nm.

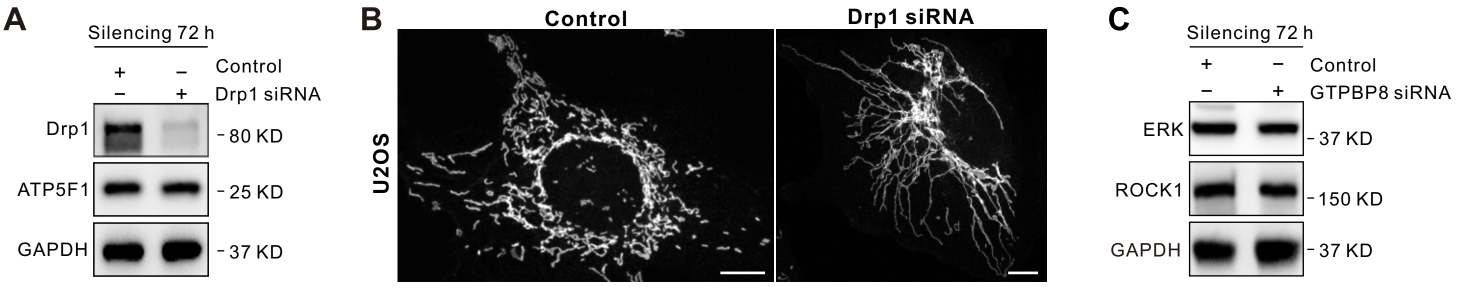

Fig. S5. Drp1 knockdown efficiency after treated with Drp1 siRNA in U2OS cells for 72 h. (A) Western blot of cell lysates after transfected with control and Drp1 siRNA for 72 h. TOM40 β-Actin, and ATP5F1 are loading controls. **(B)** The representative images show mitochondrial morphology in U2OS cells treated with control and Drp1 siRNA for 72 h. Mitochondria were visualized by immunofluorescence against TOM20. Scale bar, 10 μm. **(C)** Western blot of cell lysates after transfected with control and GTPBP8 siRNA 4# for 72 h. GAPDH is loading control.

Journal of Cell Science • Supplementary information

Fig. 1A

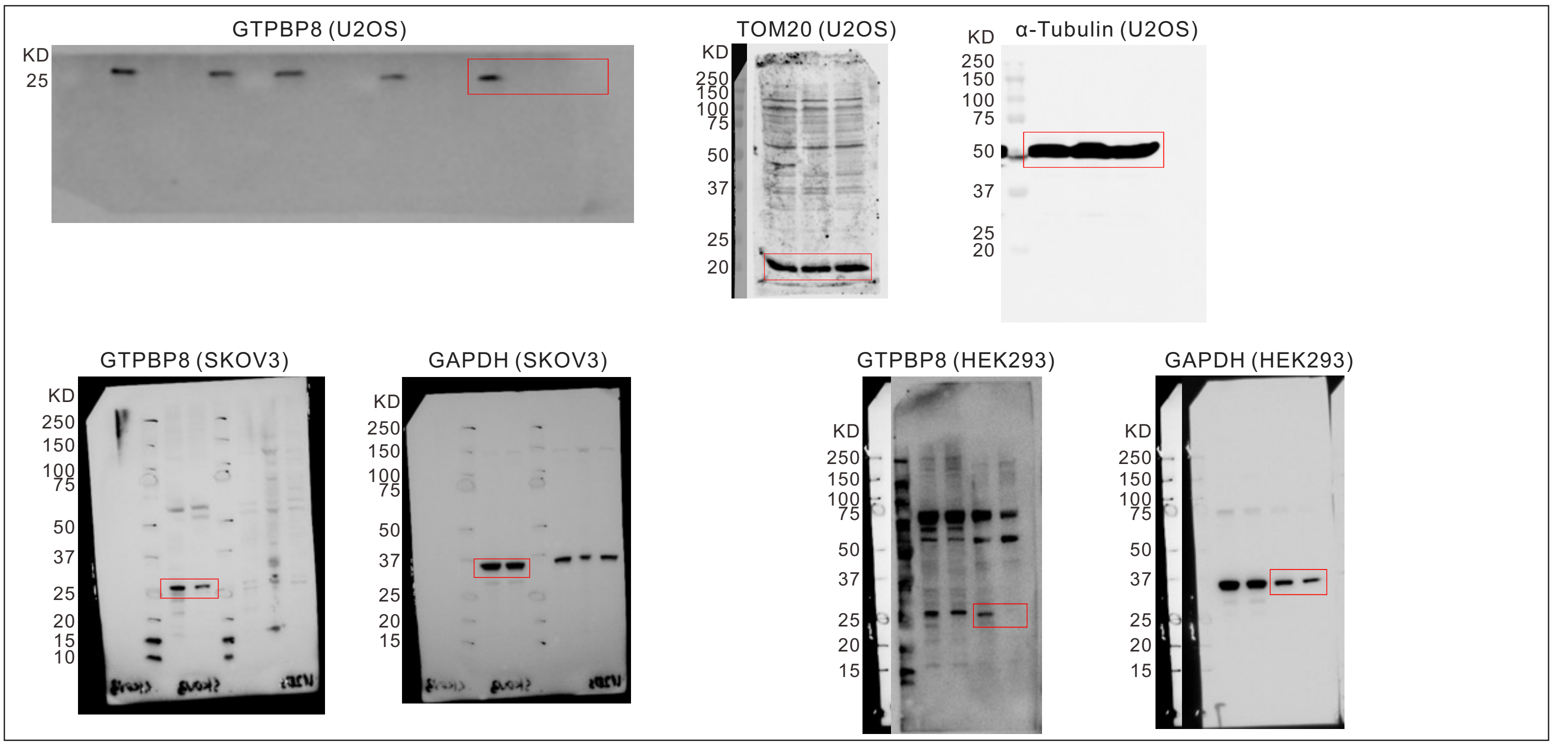

Fig. 5A

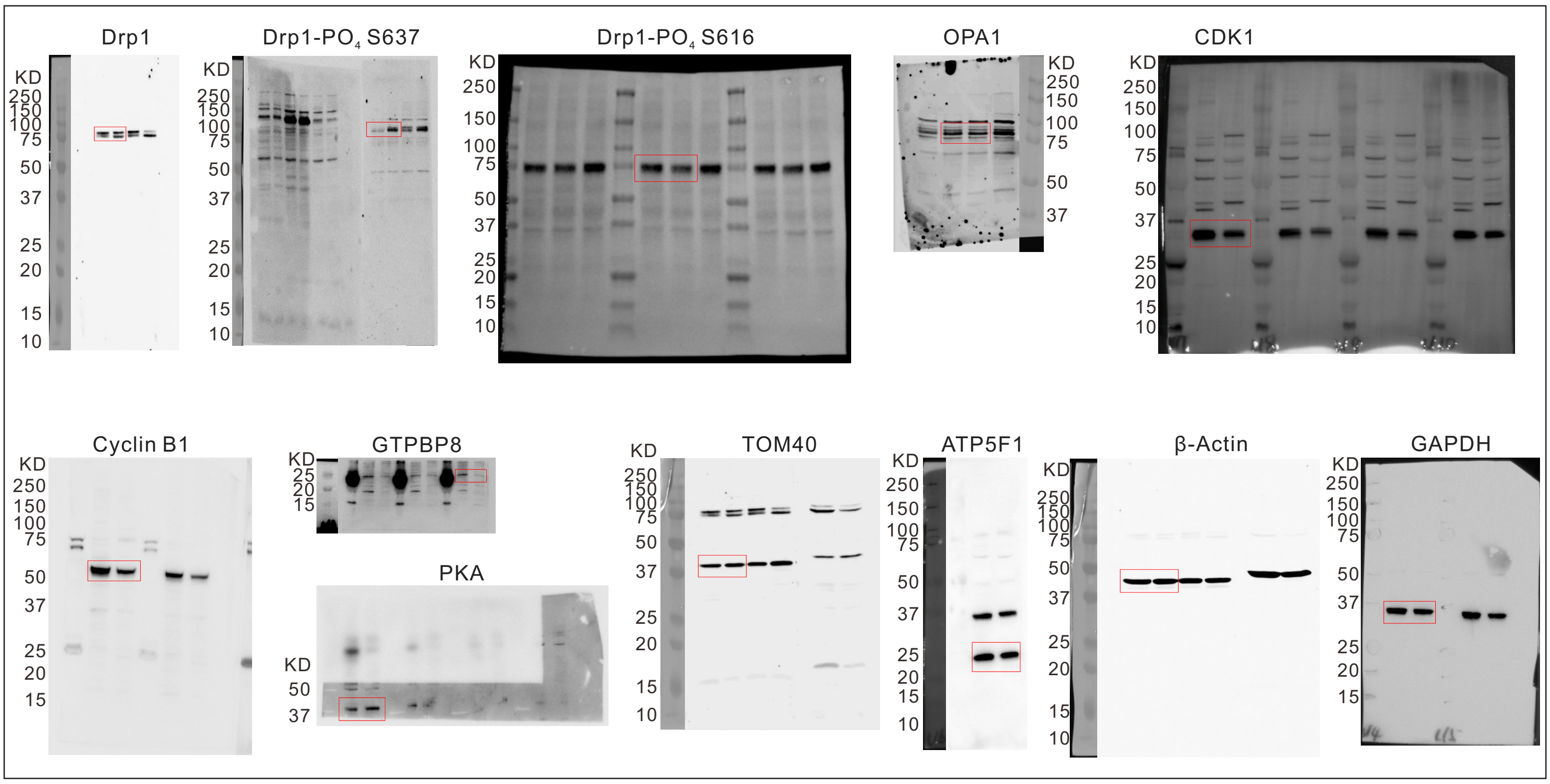

Journal of Cell Science • Supplementary information

Fig. 5D

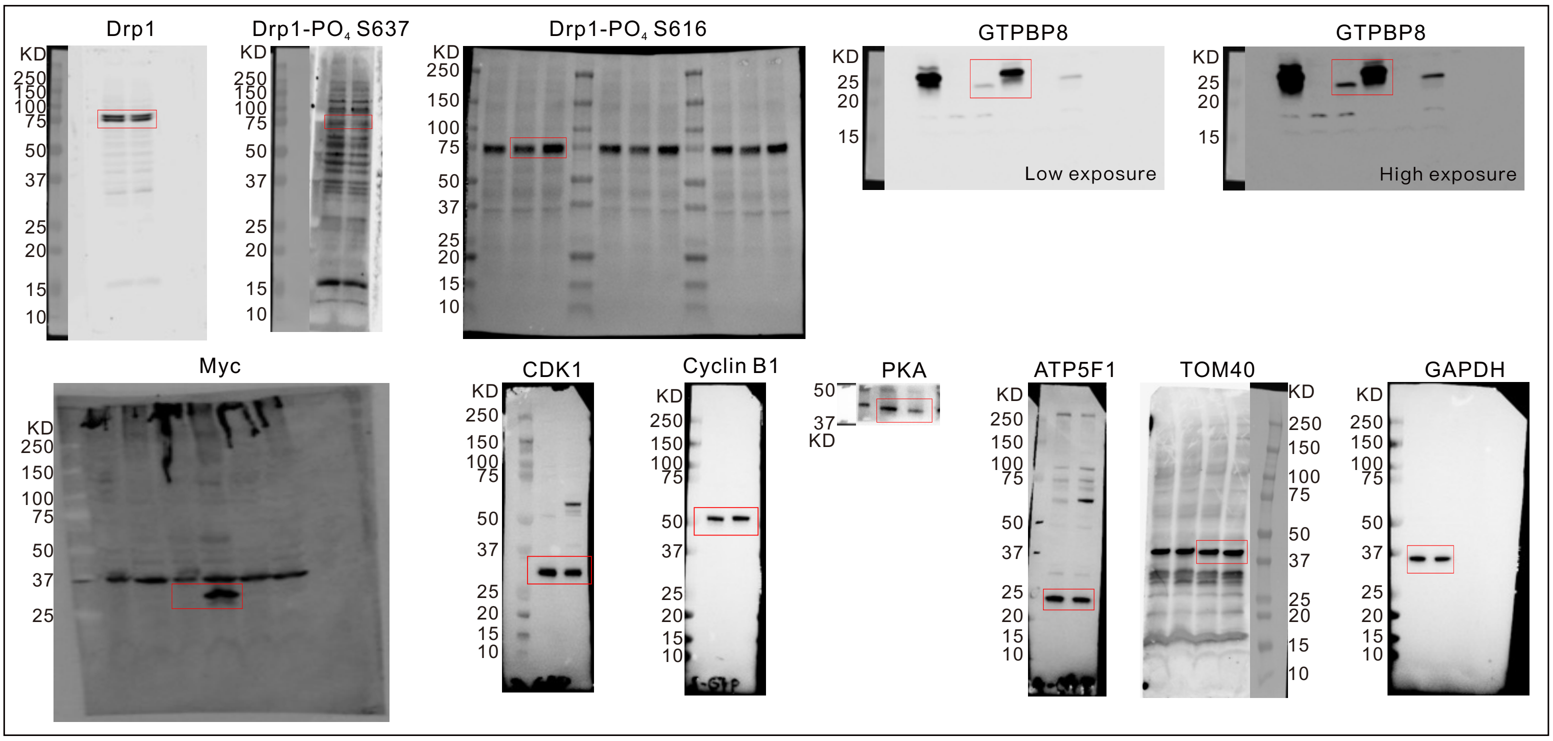

Fig.5G

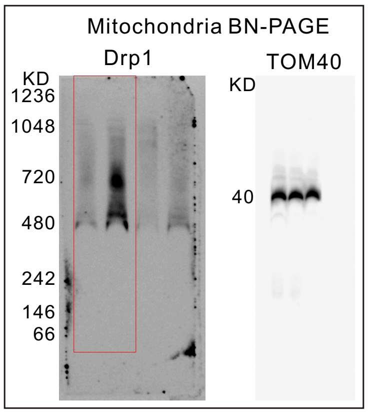

Fig.S2A

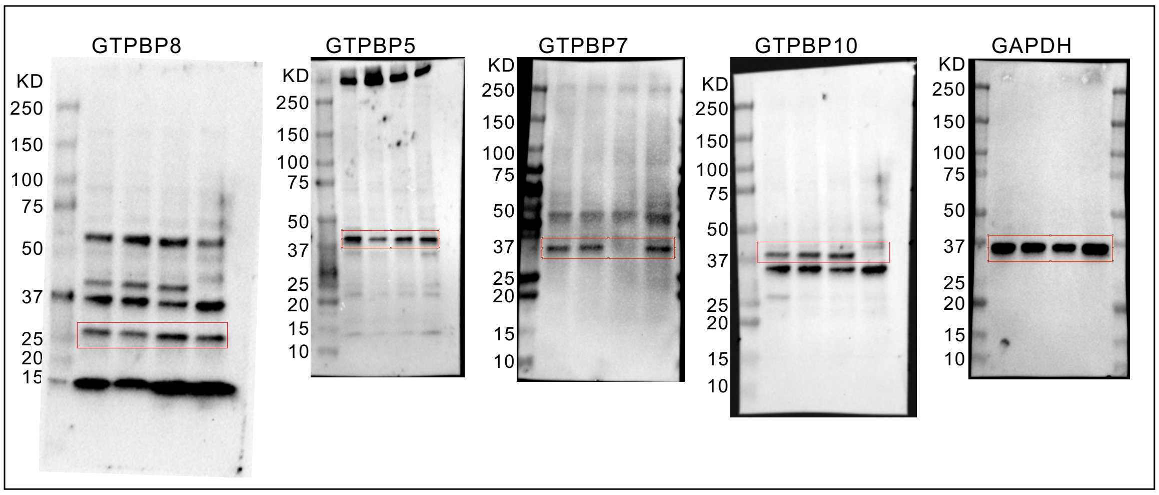

Fig.S5A

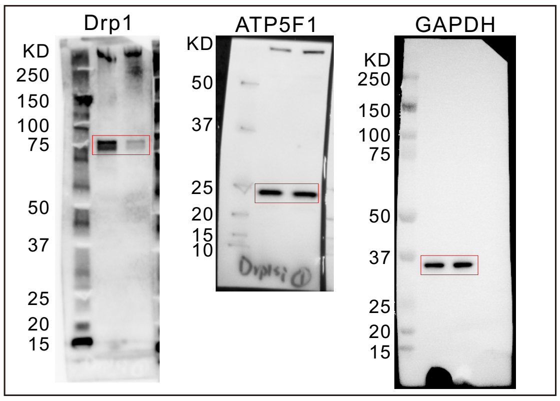

Fig. S5C

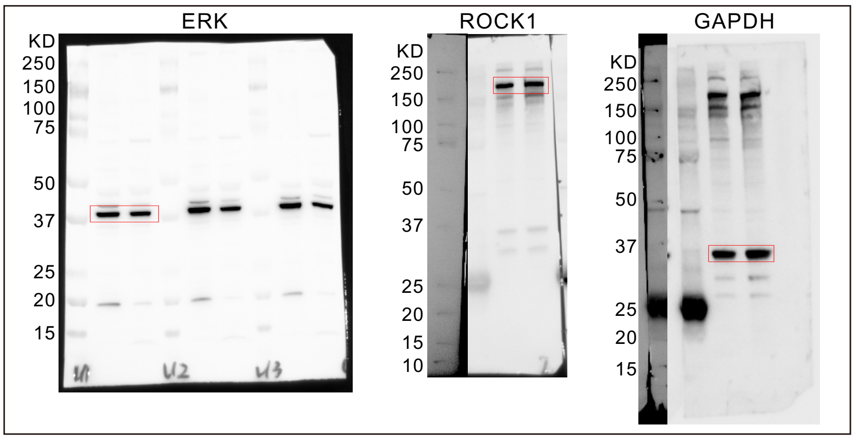

Fig. S6. Blot Transparency

Table S1. Antibodies used in this study.

Available for download at
https://journals.biologists.com/jcs/article-lookup/doi/10.1242/jcs.261612#supplementary-data

Journal of Cell Science • Supplementary information

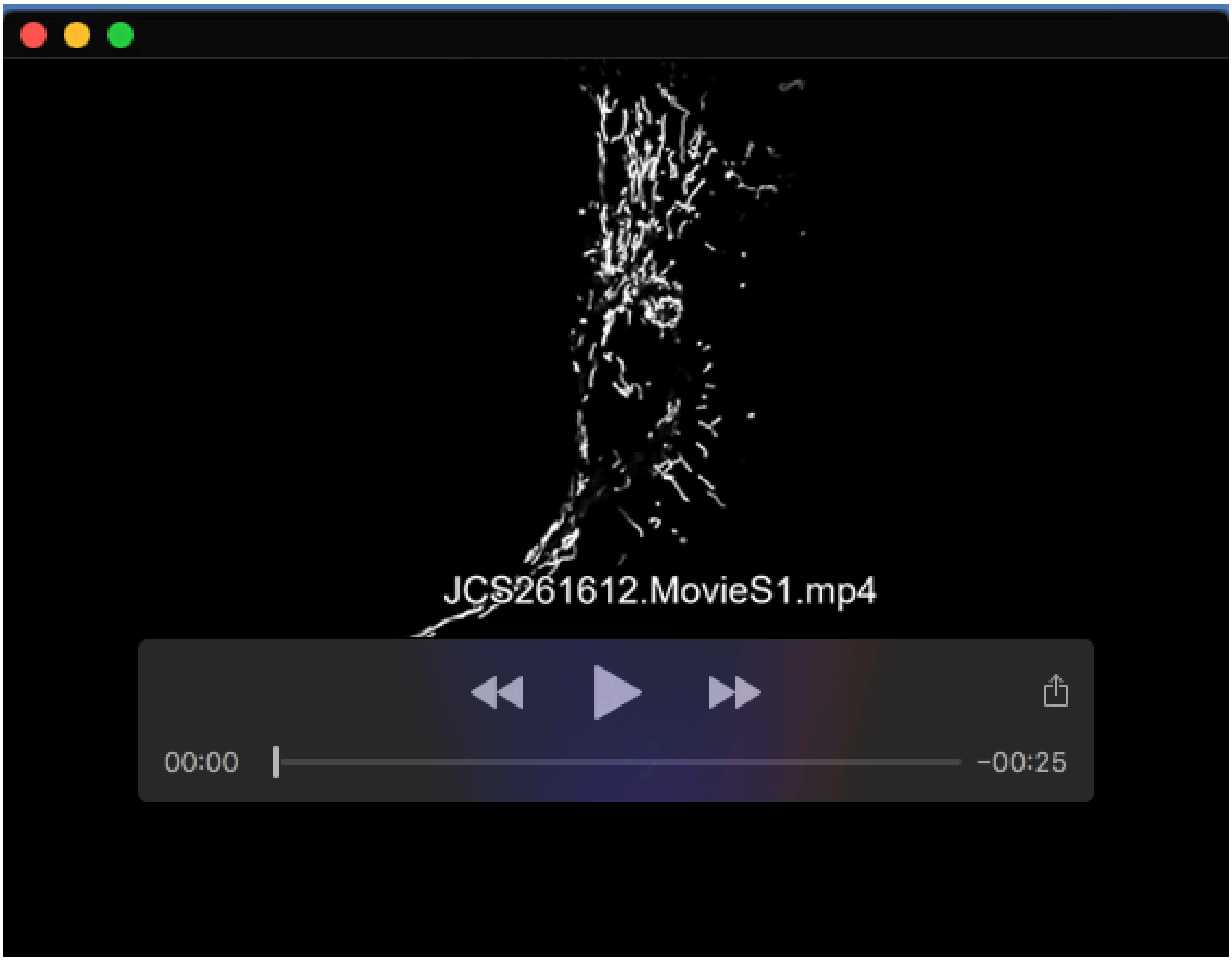

Movie 1. Time-lapse movie of mitochondria visualized in control cells expressing Mito-DsRed, related to Fig. 2A. The time-lapse movie was obtained for 10 minutes with 10 s intervals using 3I Marianas microscope. The display rate is 5 frames per second.

Journal of Cell Science • Supplementary information

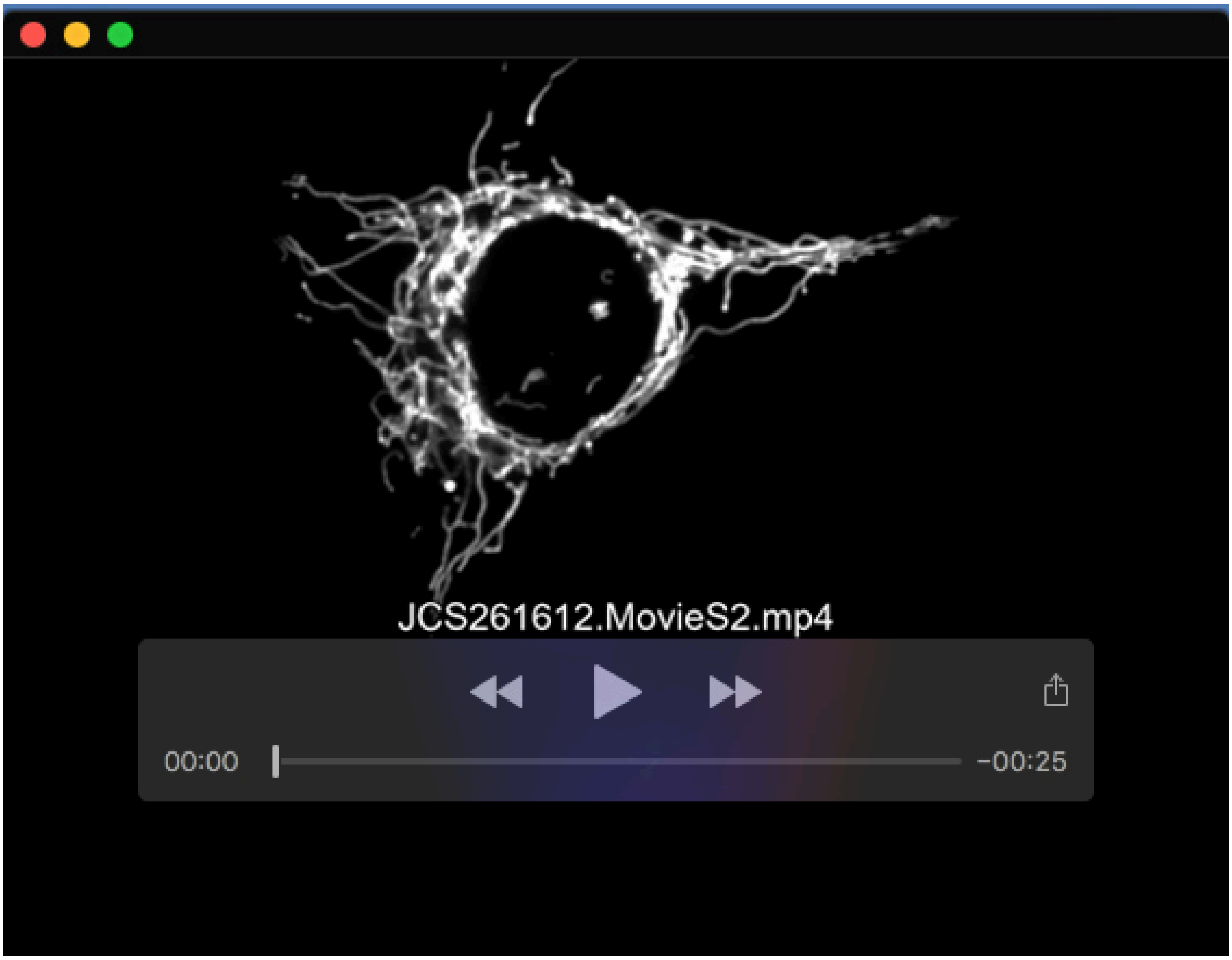

Movie 2. Time-lapse movie of mitochondria visualized in GTPBP8 knockdown cells expressing Mito-DsRed, related to Fig. 2A. The time-lapse movie was obtained for 10 minutes with 10 s intervals using 3I Marianas microscope. The display rate is 5 frames per second.

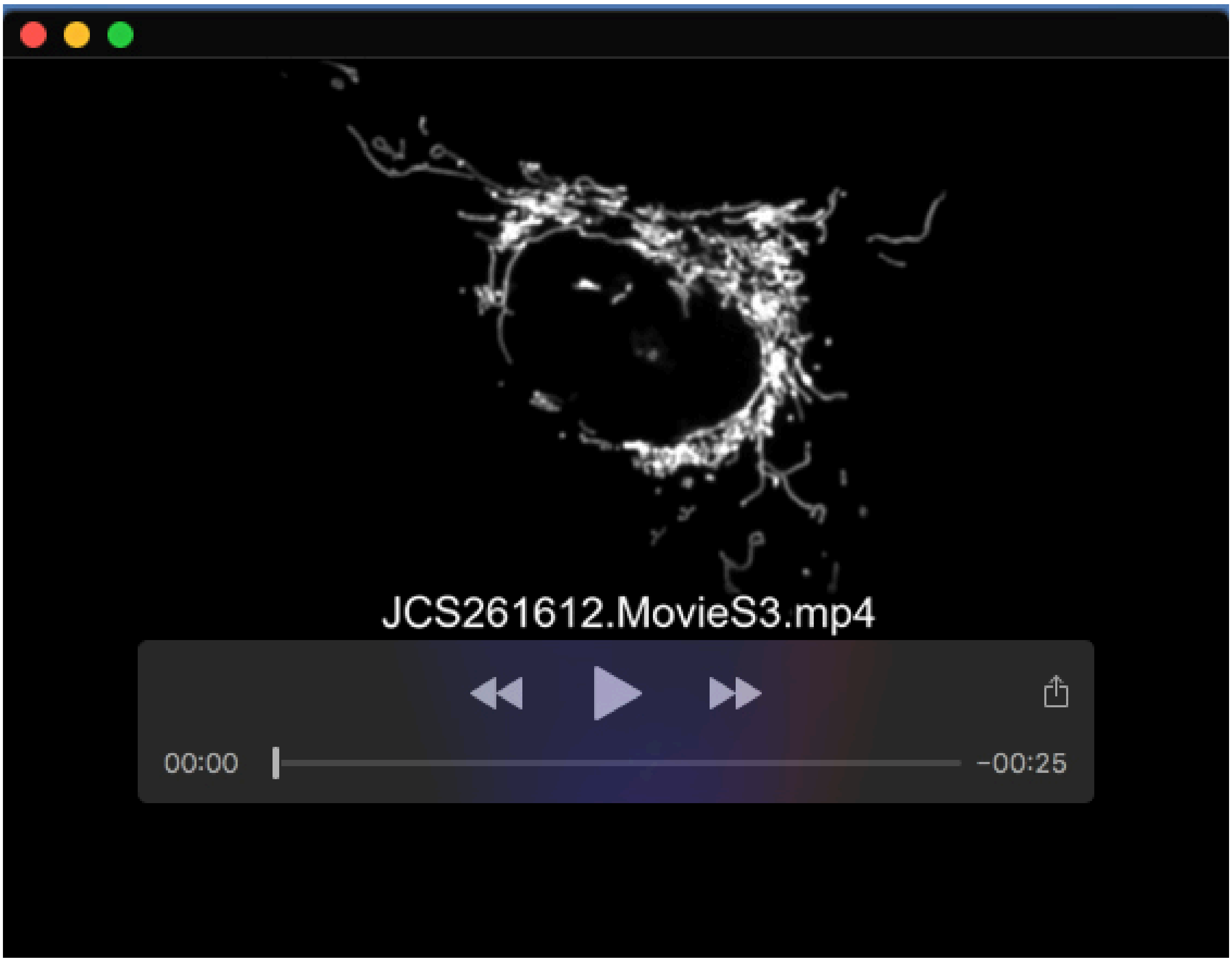

Movie 3. Time-lapse movie of mitochondria visualized in control cells expressing Mito-DsRed, related to Fig. 3K. The time-lapse movie was obtained for 10 minutes with 10 s intervals using 3I Marianas microscope. The display rate is 5 frames per second.

Journal of Cell Science • Supplementary information

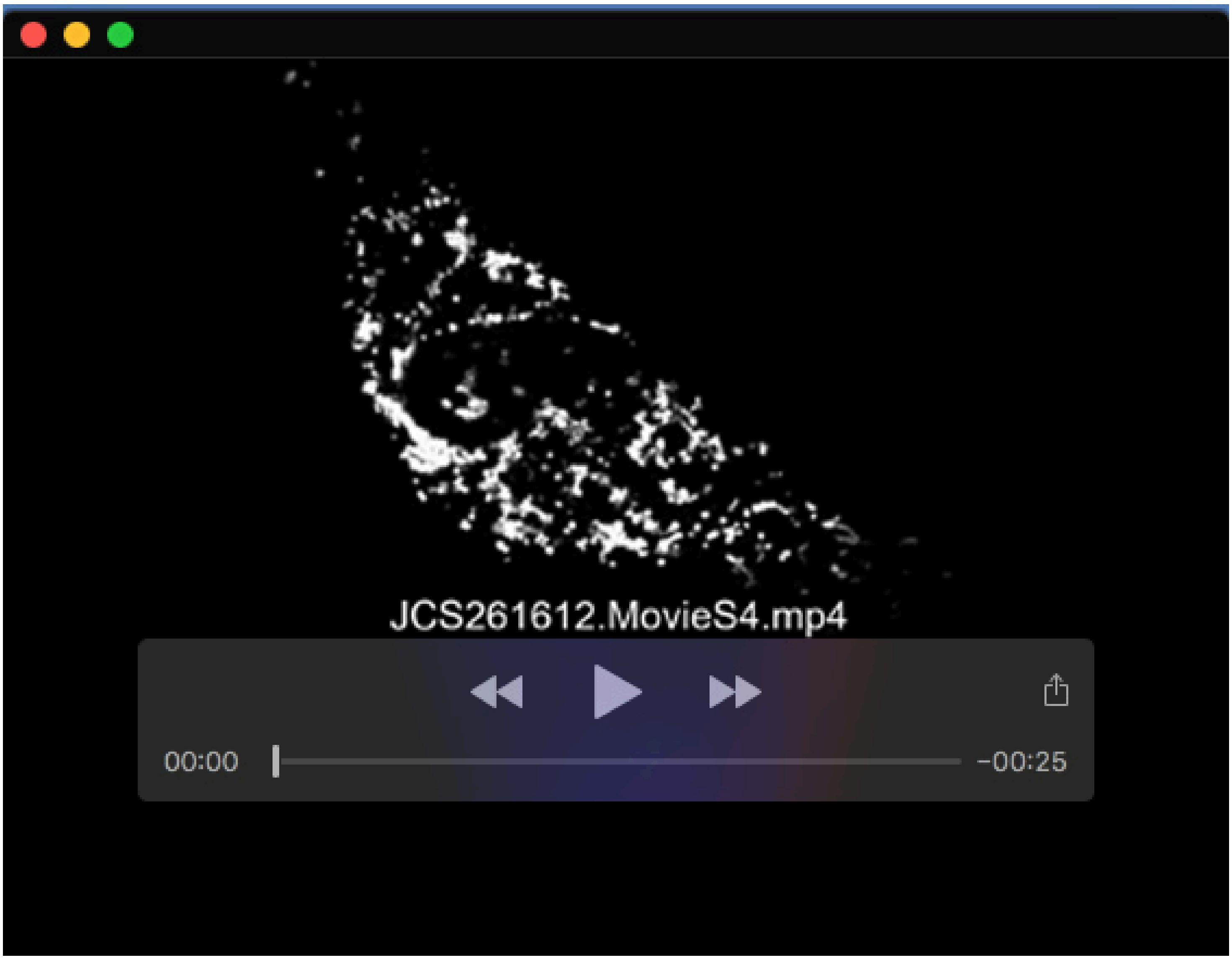

Movie 4. Time-lapse movie of mitochondria visualized in GTPBP8-GFP expressed U2OS cells, related to Fig. 3K. The time-lapse movie was obtained for 10 minutes with 10 s intervals using 3I Marianas microscope. The display rate is 5 frames per second.

Journal of Cell Science • Supplementary information
