# Supplementary material for: GTPBP8 modulates mitochondrial fission through a Drp1-dependent process
Source: J Cell Sci. 2024 Apr 30;137(8):jcs261612. doi: 10.1242/jcs.261612 (PMC11112121; doi:10.1242/jcs.261612)
Supplement: Supplementary file 1 [file joces-137-261612_review_history.pdf]

Journal of Cell Science

GTPBP8 modulates mitochondrial fission through a Drp1-dependent process

Xiumei He, Liang Wang, Hoi Ying Tsang, Xiaonan Liu, Xiaofeng Yang, Shiming Pu, Ziqi Guo, Cheng Yang, Qiong Wu, Zuping Zhou, Xiaobo Cen and Hongxia Zhao
DOI: 10.1242/jcs.261612

Editor: Jennifer Lippincott-Schwartz

Review timeline

Original submission:	7 September 2023
Editorial decision:	1 November 2023
First revision received:	1 February 2024
Editorial decision:	7 March 2024
Second revision received:	13 March 2024
Accepted:	26 March 2024

Original submission

First decision letter

MS ID#: JOCES/2023/261612

MS TITLE: GTPBP8 Regulates Mitochondrial Fission through Drp1

AUTHORS: Xiumei He, Liang Wang, Ziqi Guo, Yang Cheng, Qiong Wu, Zuping Zhou, and Hongxia Zhao
ARTICLE TYPE: Research Article

We have now reached a decision on the above manuscript.

To see the reviewers' reports and a copy of this decision letter, please go to: https://submit-jcs.biologists.org and click on the 'Manuscripts with Decisions' queue in the Author Area. (Corresponding author only has access to reviews.)

As you will see, the reviewers gave favourable reports but raised some critical points that will require amendments to your manuscript. I hope that you will be able to carry these out because I would like to be able to accept your paper, depending on further comments from reviewers.

Please ensure that you clearly highlight all changes made in the revised manuscript. Please avoid using 'Tracked changes' in Word files as these are lost in PDF conversion.

I should be grateful if you would also provide a point-by-point response detailing how you have dealt with the points raised by the reviewers in the 'Response to Reviewers' box. Please attend to all of the reviewers' comments. If you do not agree with any of their criticisms or suggestions please explain clearly why this is so.

Reviewer 1

Advance summary and potential significance to field

In this manuscript, He et al identify a correlative relationship between expression levels of the mitochondrial matrix protein GTPBP8 and mitochondrial morphology. GTPBP8 was established by

© 2024. Published by The Company of Biologists under the terms of the Creative Commons Attribution License (https://creativecommons.org/licenses/by/4.0/).

the authors in a recent preprint to be a GTPase localized to the mitochondrial matrix and involved in mitoribosome biogenesis. Here, the authors demonstrate that knockdown of GTPBP8 leads to mitochondrial thinning and elongation and that its overexpression causes Drp1-dependent mitochondrial fragmentation. Seeking a mechanistic explanation for the knockdown phenotype, the authors determine that Drp1 S637 phosphorylation levels are increased in cells depleted of GTPBP8, and that this correlates with reduced Drp1 recruitment to mitochondria. Based on these results, the authors argue that GTPBP8 directly regulates Drp1-mediated mitochondrial fission. The experiments are generally well performed and clearly presented, with exception of characterization of mitochondrial dynamics in live cells (see below).

Comments for the author

However, the overarching conclusion of the manuscript that GTPBP8 regulates mitochondrial fission is not supported. In fact, given that the authors have simultaneously demonstrated a role for GTPBP8 as a mitoribosome factor in their preprint, the changes in mitochondrial morphology are likely indirect. The manuscript would thus be strengthened by exploring the relationship between mitoribosome defects and mitochondrial dynamics. However, at a minimum to support publication, the conclusions must be significantly toned down as there are no data presented supporting models in which GTPBP8 is a direct regulator of fission.

Specific points:
1. The authors demonstrate that mitochondria morphology is influenced by knockdown of GTPBP8 in two ways. First, the mitochondria appear more elongated and second, the mitochondria appear thinner by EM analysis. Is it possible that the thinning of mitochondria causes the appearance of elongation without being an indicator of reduced Drp1-dependent fission?
2. Related to this point, the authors claim to have quantitatively assessed mitochondrial fission/fusion dynamics in Figure 2A (line 162) and Figure 3K (line 217). However, this does not appear to be the case. The only data presented are timelapse examples of one single control cell and one single knockdown/OE cell with no quantification. The knockdown cell does not have the morphological phenotype indicated in Figure 1, and fission is documented to occur without issue in the cell. To claim that there is an inbalance in fission and fusion, the authors must perform this analysis on several cells in multiple experimental replicates and perform a quantitative assessment.
3. Is it possible that GTPBP8 knockdown is leading to a generic stress response due to reduced mitochondrial translation efficiency? For example knockdown of LRPPRC, which is involved in mitochondrial transcription, was demonstrated to lead to mitochondrial hyperfusion as a compensatory response to improve respiratory activity (Rolland et al, PNAS, 2013). Does modulation of the mitoribosome by altering expression of other mitoribosomal assembly factors also correlate with the same effects on mitochondrial morphology?

<u>Reviewer 2</u>

Advance summary and potential significance to field

GTPBP8 is a newly identified GTP-binding protein, whose discovery by this very group is currently in BioRxiv (https://www.biorxiv.org/content/10.1101/2023.05.26.542385v2.full.pdf). In this BioRxiv, GTPBP8 showed low intrinsic GTPase activity and localization in the mitochondria, specifically the matrix with some IMM interaction. Depletion of GTPBP8 (siRNA) diminished oxygen consumption rates in a standard Seahorse Assay (basal, ETC leak, maximal, and non-mitochondrial OCR). Moreover, protein levels of subunits in Complex I, III IV, and the ATP synthase were reduced, suggestive of reduced mitochondrial energy production. The exclusion of Complex II led the authors to hypothesize/test a potential role in mitoribosome function, however it's unclear if mtDNA maintenance/propagation is also important in the process.

In this study, He et al. study the relationship between GTPBP8 and mitochondrial fusion/fission. The authors present clear and consistent evidence that knockdown or overexpression of GTPBP8 is well correlated with fused mitochondria and fragmented mitochondria, respectively. The authors show this is dependent on Drp1 with the phosphorylation status of Drp1 as an important variable of fission/fusion. The study is well written polished, figures are clear, and presents a consistent story. However, there is little mechanistic insight into how mitochondrial GTPBP8 regulates cytosolic

© 2024. Published by The Company of Biologists under the terms of the Creative Commons Attribution License (https://creativecommons.org/licenses/by/4.0/).

Drp1, and most importantly if this pathway is a direct mechanism or a consequence of a mitochondrial energetic disturbances, as has been demonstrated for small molecule Honokiol (https://www.ncbi.nlm.nih.gov/pmc/articles/PMC5512068/).

Comments for the author

Given that there's no mechanism connecting GTPBP8 to Drp1, and this process is entirely dependent on traditional OMM fusion/fission machinery, the authors should be more conservative in their conclusive statements. For example, a more accurate title would be "GTPBP8 regulates mitochondrial fission through a Drp1-dependent process". In the concluding statements, it's not clear if 'GTPBP8 and Drp1 collaborate in orchestrating mitochondrial fission' or if GTPBP8, and the consequences of its knockdown/overexpression are causing mitochondrial stress, which is changing Drp1 dependent mitochondrial dynamics.
There are few concerns with the core findings (GTPBP8 expression is correlated with fusion/fission of mitochondria) as they are well supported. The following major and minor comments should still be addressed:

Major:
1) Figure 1C: how were cells/mitochondria scored? Was it automated, manual, and what were the criteria for designating short, long, net, etc? The authors cite a prior study, but some description of the methods would be useful.

2) "However, no discernible alterations were observed in mitochondrial architecture following GTPBP8 knockdown. Similar to the control group, the outer membrane retained its smooth appearance enveloping the mitochondria, the inner membrane displayed extensive folding characterized by numerous cristae, and the cristae themselves maintained their characteristic tubule- or sheet-like structures, even in the absence of GTPBP8 (Fig. 1D)."
Based on the images provided, this does not appear to be true. The cristae are more pronounced, numerous and perpendicular to the membrane in control. If this is due to contrast/zoom, please provide different images. However, if these images were misinterpreted, reduced cristae would also support the conclusions of their prior study (decreased OCR, decreased ETC protein, etc).

3) "The occurrences of fission and fusion events within a fixed time frame were quantified. In comparison to control cells, GTPBP8 knockdown led to a reduction in mitochondrial fission events and an increase in fusion events (Fig. 2A)."
The authors present images where it appears there are 3 fission events and 1 fusion event in control vs. 2 fission events and 1 fusion event in KD. Did the authors intend to include a larger sample size plot where statistical confidence can be more easily interpreted? Similar comments for Figure 3K, where statistical power is low and similar concerns arise.

4) Fig S1D: Please explain why there are two bands in the Drp1 KD and why one appears reduced whereas the other increased for the siRNA lane.

5) Figure 5D, Drp1-PO4: the effects are so small compared to the 5A KDs that it is difficult to appreciate the author's point. Do the authors have any other blots for this figure? For example, more sample to increase contrast?

Minor:
1) Line 59: specific which membrane or indicate both membranes.
2) Methods: include a table summarizing antibodies with a column dedicated to 'knockout verified'.
3) Figure 1D: To compare mitochondria, it would be helpful to have similar magnification/zoom on the images.
4) Figure 1E/F/G: significant digits. Based on the scalebar of 500 nm and axes in Fig. 1H, it's unlikely analysis from this plot is accurate to 3 decimal places from um (single nm). Note that Fig. 1G extends to 0.008302 um; accuracy to single pm. The authors should correct their mean/error mathematics in the text such that decimal places reflect the limitations of the technique. Similar statistics are presented in the text for Figure 3, where decimal places extend to single nm or pm, pending the panel.

© 2024. Published by The Company of Biologists under the terms of the Creative Commons Attribution License (https://creativecommons.org/licenses/by/4.0/).

5) Fig 5A: Similar to S1D, the authors should explain why there are two Drp1 bands and why they are most interested in the change to the lower band, as the lower band was largely unaffected by Drp1 KD.

Reviewer 3

Advance summary and potential significance to field

The current manuscript “GTPBP8 Regulates Mitochondrial Fission through Drp1” by He et al explores the role of the mitochondrial localized GTPase GTPBP8 on mitochondrial dynamics. This paper does a good job of showing that up and downregulation of GTPBP8 has complementary effects on mitochondrial dynamics where GTPBP8 knockdown causes mitochondrial elongation and GTPBP8 overexpression causes mitochondrial fragmentation. They are further able to connect alterations in GTPBP8 expression with changes in Drp1 phosphorylation. They also showed that knockdown of Drp1 rescued the mitochondrial fragmentation phenotype of GTPBP8 overexpression. These data point to a role of GTPBP8 in mitochondrial function and the need to further investigate the mechanism by which it impacts Drp1 to influence mitochondrial dynamics.

Comments for the author

My enthusiasm is somewhat tempered because it is unclear how the expression of the inner mitochondrial membrane protein GTPBP8 protein is able to impact the phosphorylation of the cytosolic protein Drp1 and therefore mitochondrial fission. I believe the manuscript could be improved by changing how the manuscript is currently framed as at multiple points the paper is written implying that they hope to find mechanism of fission at the inner membrane. In the abstract they state “our understanding of the fission mechanisms within the inner mitochondrial membrane and the associated factors/proteins is limited.”
Similarly on line 308 the authors state “the precise coordination between outer and inner membrane fission events and the factors involved are yet to be defined.” But to me there is a distinction between “fission mechanisms” and “fission signaling”. Fission mechanisms implies factors that constrict the mitochondria to actually drive scission of the inner and outer mitochondrial membrane. While fission signaling does not need to be directly involved in the fission mechanism or scission of the mitochondria but instead starts a cascade to impact the actual fission machinery. Mitochondrial function is a general fission signaling mechanism as loss of membrane potential can drive mitochondrial fission. Could GTPBP8 just be generally impacting mitochondrial function and that secondarily impacts fission and fusion? While the authors cite another bioRxiv paper from their lab that GTPBP8 is an inner mitochondrial membrane protein they do not present any data that GTPBP8 is a direct fission or fusion factor.
Furthermore, on Line 310 – “In this study, we unraveled the functional significance of a protein” which I believe is an overstatement. They do not know how GTPBP8 impacts Drp1 phosphorylation or even what part of its function is needed to impact fission, but as they point out further down in the discussion it is unlikely through direct interaction. They even postulate that it could be a completely indirect effect as they have also found that GTPBP8 has a role in mitochondrial ribosomal assembly and therefore mitochondrial translation.

Other comments:
Line 163 Is there any quantification to say that fission and fusion events are changed as stated on Line 163 (Fig 2A)? From the figure there is (3 fission, 1 fusion) events in the control, while there are (2 fission, 2 fusion) events in the GTPBP8 siRNA, but these small number of events are obviously not significantly different. Is there more data than this?
Line 217 – similarly it seems like it is hard to conclude a change in fission and fusion events from two events in the control (1 fission, 1 fusion) which was the same ratio previously said to be higher fusion in the GTPBP8 siRNA. The GTPBP8 OE seems to have (3 fission, 3 fusion) which doesn’t change the ratio though it is hard to see all the arrows indicating events in these images. It also seems hard for the authors to conclude even more overall events with such low numbers.

Minor typo comments:
Line 113 – Missing “subtilis”
Line 118 – proper citation of bioRxiv paper.

© 2024. Published by The Company of Biologists under the terms of the Creative Commons Attribution License (https://creativecommons.org/licenses/by/4.0/).

Line 568, 574, 628 – Duplicated references

First revision

Author response to reviewers' comments

Point-by-Point Response to Reviewers' Comments

We deeply appreciate the insightful comments and suggestions provided by the reviewers, which have significantly improved the quality of our work. In response to these comments and suggestions, we have incorporated new data, re- analyzed existing data, and restructured specific sections of the manuscript accordingly. We believe that these additional works significantly improve the quality and comprehensiveness of our manuscript. Below, we present a detailed, point-by-point response to the reviewers' comments, with reviewer remarks shown in black and our responses in blue. Please be aware that the figure citations in our response pertain to the new (post-revision) figures. We have highlighted the corresponding changes within the manuscript in red. We look forward to your feedback and the opportunity to have our work published in the *Journal of Cell Science*.

Reviewer 1: Advance Summary and Potential Significance to Field:

In this manuscript, He et al identify a correlative relationship between expression levels of the mitochondrial matrix protein GTPBP8 and mitochondrial morphology. GTPBP8 was established by the authors in a recent preprint to be a GTPase localized to the mitochondrial matrix and involved in mitoribosome biogenesis. Here, the authors demonstrate that knockdown of GTPBP8 leads to mitochondrial thinning and elongation and that its overexpression causes Drp1- dependent mitochondrial fragmentation. Seeking a mechanistic explanation for the knockdown phenotype, the authors determine that Drp1 S637 phosphorylation levels are increased in cells depleted of GTPBP8, and that this correlates with reduced Drp1 recruitment to mitochondria. Based on these results, the authors argue that GTPBP8 directly regulates Drp1-mediated mitochondrial fission. The experiments are generally well performed and clearly presented, with exception of characterization of mitochondrial dynamics in live cells (see below).

Reviewer 1 Comments for the Author:
However, the overarching conclusion of the manuscript that GTPBP8 regulates mitochondrial fission is not supported. In fact, given that the authors have simultaneously demonstrated a role for GTPBP8 as a mitoribosome factor in their preprint, the changes in mitochondrial morphology are likely indirect. The manuscript would thus be strengthened by exploring the relationship between mitoribosome defects and mitochondrial dynamics. However, at a minimum to support publication, the conclusions must be significantly toned down as there are no data presented supporting models in which GTPBP8 is a direct regulator of fission.
Response: We thank the reviewer for the thoughtful comments and insights into our study. As the reviewer pointed out, the role of GTPBP8 in the regulation of mitochondrial fission is likely indirect but is involved in the fission signaling pathway. We have modified the conclusion and title of the manuscript. The title of the manuscript has been changed to "GTPBP8 Regulates Mitochondrial Fission through a Drp1-Dependent Process". Following the suggestions, we have conducted additional experiments to address the role of GTPBP8 in mitochondrial fission signaling.

1) First of all, to assess whether the changes caused by GTPBP8 silencing are cell-type-specific, we additionally silenced GTPBP8 in two other mammalian cell lines, SKOV3 and HEK293. Similar to U2OS cells, the loss of GTPBP8 in these two cells led to comparable elongation and hyperfusion of mitochondria (**Fig. 1A-C**). Conversely, the overexpression of GTPBP8-GFP in these cells resulted in an increased proportion of cells with fragmented mitochondrial morphology (**Fig. S3C and S3D-F**). Collectively, the consistent changes in mitochondrial morphology among multiple cell types in response to altered GTPBP8 levels suggest that the maintenance of GTPBP8 is critical for mitochondrial dynamics.

© 2024. Published by The Company of Biologists under the terms of the Creative Commons Attribution License (https://creativecommons.org/licenses/by/4.0/).

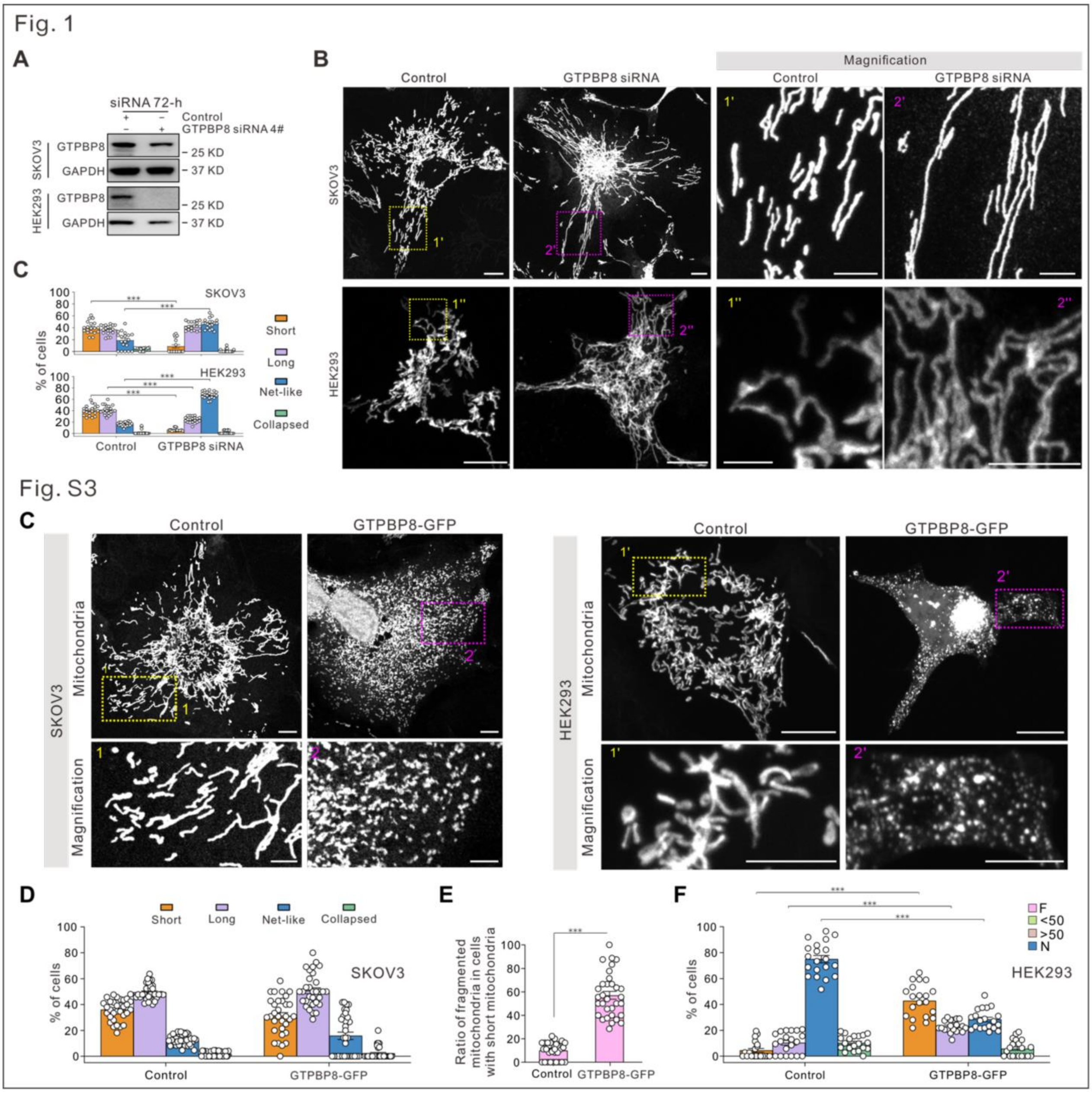

2) Our work shows that GTPBP8 is a mitoribosomal assembly factor and resides in both the mitochondrial inner membrane and matrix. However, GTPBP8 interacts with MRPL16 and MRPL31, which do not localize closely to the inner mitochondrial membrane as MRPL45 does (Wang et al., 2023). The function of membrane-associated GTPBP8 remains to be elucidated. To investigate whether the role of GTPBP8 in mitoribosomal assembly is involved in mitochondrial fission signaling, we examined the changes in mitochondrial morphology after depleting three other mitoribosomal assembly factors, GTPBP5 (Maiti et al., 2020), GTPBP7 (Kotani et al., 2013), and GTPBP10 (Lavdovskaia et al., 2018). The results showed that only GTPBP5 knockdown resulted in net-like mitochondrial morphology, whereas GTPBP7 and GTPBP10 knockdown were unable to induce the hyperfusion of mitochondria (**Fig. S2A-D**). According to the reduced oxygen consumption reported in the GTPBP5-, GTPBP7-, and GTPBP10- deficient cells (Kim and Barrientos, 2018; Cipullo et al., 2021a; Maiti et al., 2018), these data suggest that not all cells in a low-energy state present elongated or interconnected mitochondrial morphology. Although loss of GTPBP5 led to hyperfused mitochondrial morphology, cells with exogenously expressed GTPBP5::FLAG still present tubular mitochondrial morphology as in the non-transfected cells (Cipullo et al., 2021a). Moreover, we observed that a small proportion of GTPBP7 knockdown cells even displayed fragmented mitochondrial morphology (**Fig. S2C and S2D**). By detecting the protein level of GTPBP8 in the GTPBP5-, GTPBP7-, and GTPBP10-knockdown cells, we found that GTPBP8 was up-regulated

© 2024. Published by The Company of Biologists under the terms of the Creative Commons Attribution License (https://creativecommons.org/licenses/by/4.0/).

following the loss of GTPBP7 while with less alteration in the GTPBP5 and GTPBP10 knockdown cells (**Fig. S2B**). These data suggest the important role of GTPBP8 in regulating mitochondrial dynamics.

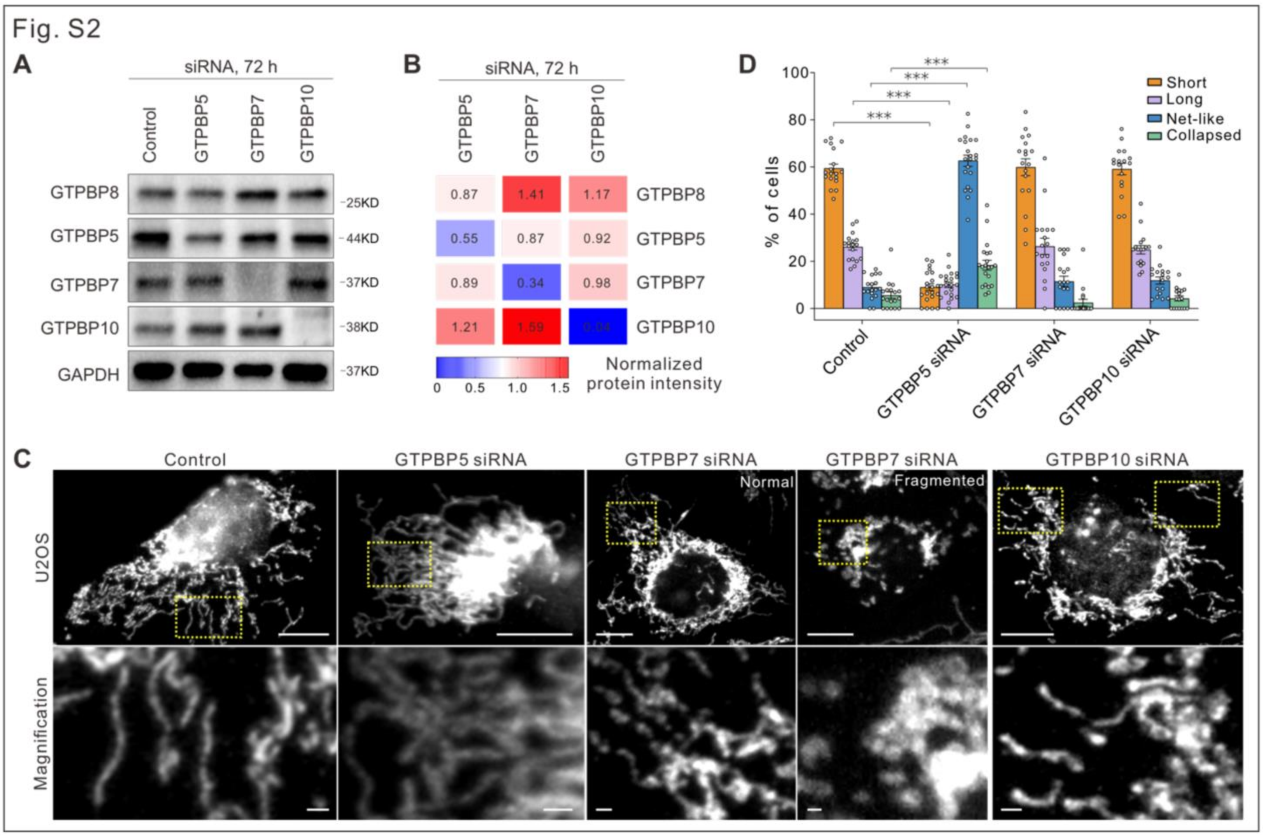

3) Furthermore, we examined the effects of GTPBP8 depletion on the localization and copy number of mtDNA, mitochondrial membrane potential, and the production of cellular reactive oxygen species. In contrast to control cells, GTPBP8 knockdown caused a significant increase in oxidative stress but had no effect on mitochondrial membrane potential, the copy number and localization of mtDNA (**Fig. 1I-1K**).

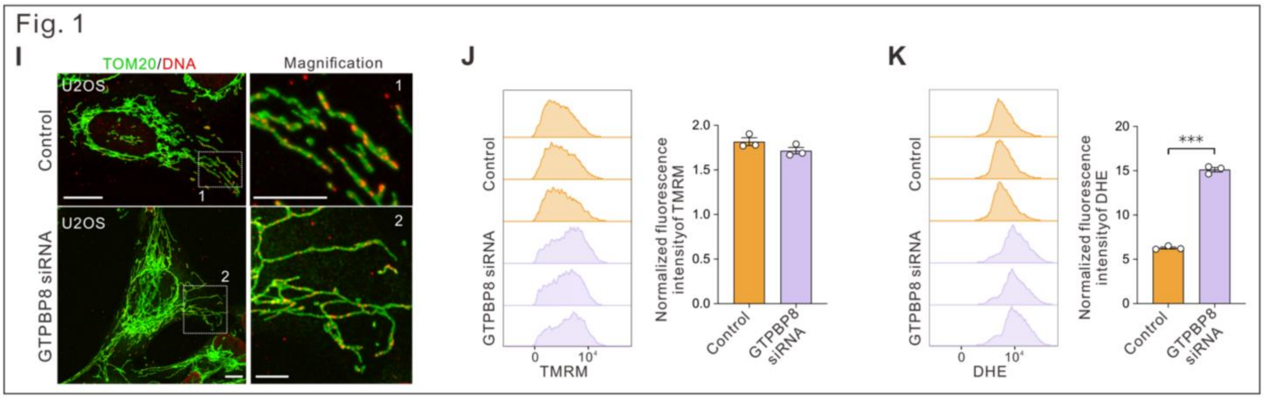

4) Drp1 activity is dependent on its activation by different post-translational modifications, such as phosphorylation and SUMOylation (Figueroa-Romero et al., 2009; Xu et al., 2016a). Phosphorylation of Drp1 at Ser616 can be modulated by different protein kinases involved in distinct signaling pathways, including protein kinase C (PKC), CDK1/Cyclin B, or ROCK, to promote fission (Lima et al., 2018; Han et al., 2008). Conversely, phosphorylation of Drp1 at Ser637 by protein kinase A (PKA) inhibits fission (Han et al., 2008). To determine whether the up-regulated Drp1 S637-PO4 level in GTPBP8-depleted cells was regulated by those kinases, we examined the expression level of CDK1, Cyclin B, ROCK1, ERK, and PKA. As shown in **Fig. 5A and Fig. S5C**, the level of PKA was up-regulated, while the levels of CDK1 and Cyclin B were down-regulated after silencing

© 2024. Published by The Company of Biologists under the terms of the Creative Commons Attribution License (https://creativecommons.org/licenses/by/4.0/).

GTPBP8 in U2OS cells. The expression levels of ROCK1 and ERK were unaffected following the depletion of GTPBP8.

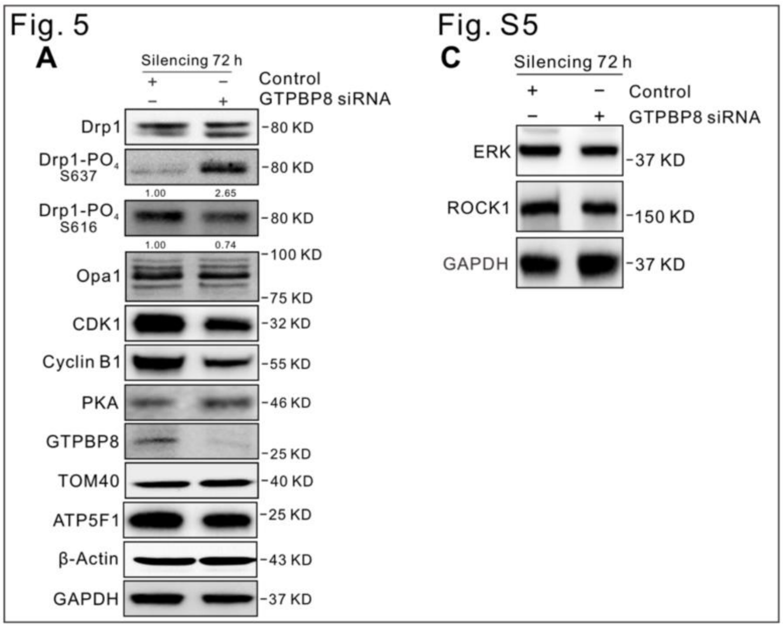

Collectively, our new data point to the possibility that GTPBP8 depletion caused defects in the assembly of mitoribosomal large subunit (mtLSU), resulting in compromised mitochondrial translation and, consequently, impaired mitochondrial function. This mitochondrial dysfunction might further regulate cell signaling involved in the phosphorylation of Drp1, leading to the increased phosphorylation of Drp1 at Ser637, the reduced recruiting of Drp1 to mitochondria, and eventually decreased mitochondrial fission.

Specific points:

1. The authors demonstrate that mitochondria morphology is influenced by knockdown of GTPBP8 in two ways. First, the mitochondria appear more elongated, and second, the mitochondria appear thinner by EM analysis. Is it possible that the thinning of mitochondria causes the appearance of elongation without being an indicator of reduced Drp1- dependent fission?

Reply: We appreciate the reviewer for bringing up this insightful point. If the elongated mitochondrial morphology was caused by the thinning of mitochondria, the mitochondrial perimeter should be comparable between the control and GTPBP8 knockdown cells. Indeed, measuring the perimeter of each mitochondria revealed an increased perimeter upon the silencing of GTPBP8 (**Fig. 1E and 1F**), suggesting the elongated mitochondrial morphology is not simply attributed to the reduced mitochondrial width. Additionally, to visualize the difference in mitochondrial morphology between the control and GTPBP8 knockdown cells, we provided more electron microscopic images of mitochondria with the same scale in the supplementary figures (**Fig. S1C**). In contrast to control cells, many mitochondria in the GTPBP8 knockdown cells were interconnected.

© 2024. Published by The Company of Biologists under the terms of the Creative Commons Attribution License (https://creativecommons.org/licenses/by/4.0/).

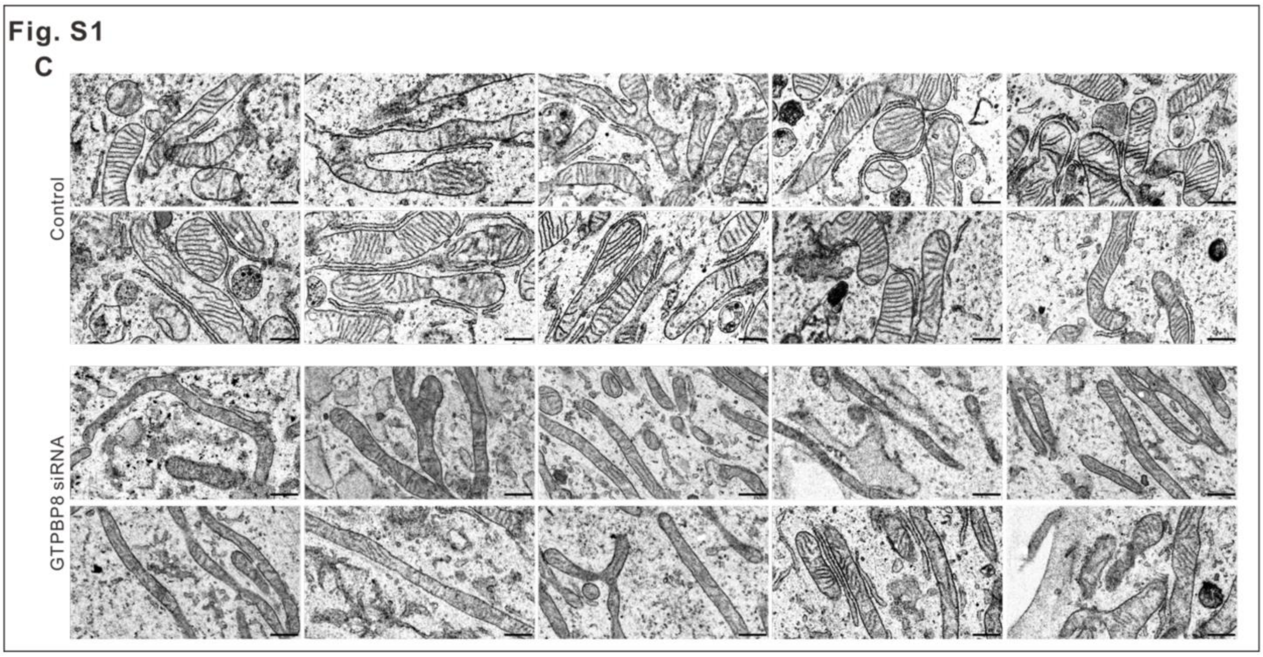

The previous representative images were presented with a 20-second interval, which might not capture all fission or fusion events. In response to the suggestions, we have now displayed all images at each time interval (**Fig. 2A and 3K**). To investigate whether changes in mitochondrial morphology were linked to an imbalance in mitochondrial dynamics, we manually counted the fission and fusion events within a 10-minute mitochondrial tracking window. In comparison to the balanced fission and fusion events in the control cells, the frequency of fission events was reduced following the loss of GTPBP8 (**Fig. 2B**). Conversely, the frequency of fission events was increased in GTPBP8-GFP overexpressed cells (**Fig. 3L**). Furthermore, the recruitment of Drp1 to the mitochondrial outer membrane (MOM) was decreased after silencing GTPBP8, while Drp1 localization on the MOM in GTPBP8-overexpressed cells was increased (**Fig. 5B and 5E**), supporting the notion that this altered mitochondrial morphology is related to the Drp1- dependent imbalance of mitochondrial dynamics.

© 2024. Published by The Company of Biologists under the terms of the Creative Commons Attribution License (https://creativecommons.org/licenses/by/4.0/).

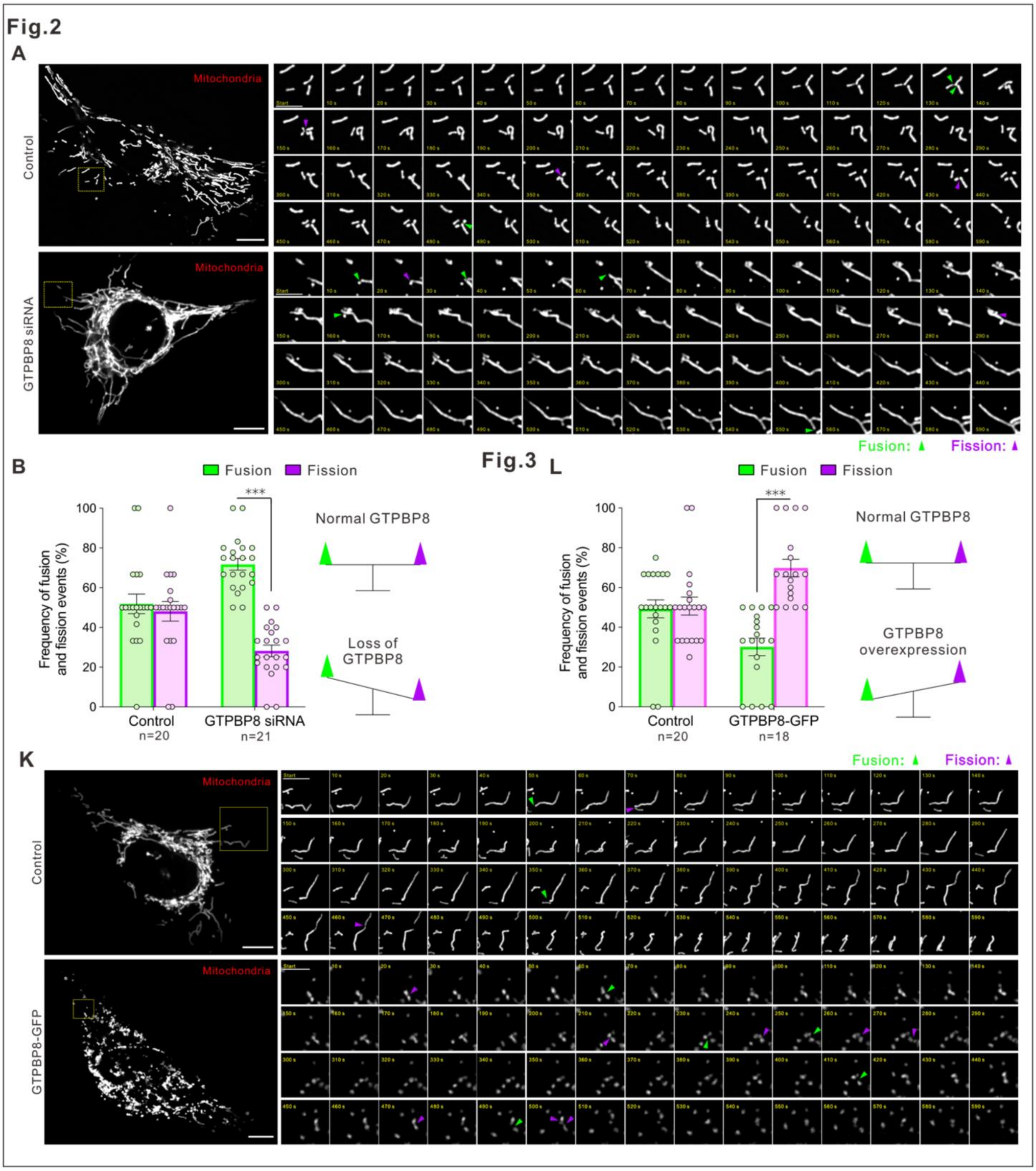

2. Related to this point, the authors claim to have quantitatively assessed mitochondrial fission/fusion dynamics in Figure 2A (line 162) and Figure 3K (line 217). However, this does not appear to be the case. The only data presented are timelapse examples of one single control cell and one single knockdown/OE cell with no quantification. The knockdown cell does not have the morphological phenotype indicated in Figure 1, and fission is documented to occur without issue in the cell. To claim that there is an inbalance in fission and fusion, the authors must perform this analysis on several cells in multiple experimental replicates and perform a quantitative assessment.

Reply: We appreciate the reviewer's valuable comments. We manually counted the fission and fusion events within a 10-minute mitochondrial tracking window. By counting the number of fission events and fusion events in 10 minutes, the ratio of fission and fusion events was calculated as follows:

Ratio of fission = The total number of fission events / the total number of fission and

© 2024. Published by The Company of Biologists under the terms of the Creative Commons Attribution License (https://creativecommons.org/licenses/by/4.0/).

fusion events *100

Ratio of fusion = The total number of fusion events / the total number of fission and fusion events *100

Compared to the balanced fission and fusion in the control cells, the frequency of fission events was reduced after silencing GTPBP8 in U2OS cells (**Fig. 2B**). Conversely, GTPBP8 overexpression resulted in an increased frequency of mitochondrial fission (**Fig. 3L**). These data suggest that the elongated or fragmented mitochondrial morphology in the GTPBP8 aberrant cells was mainly attributed to an imbalanced mitochondrial fusion and fission but not a loss of fission or fusion capabilities.

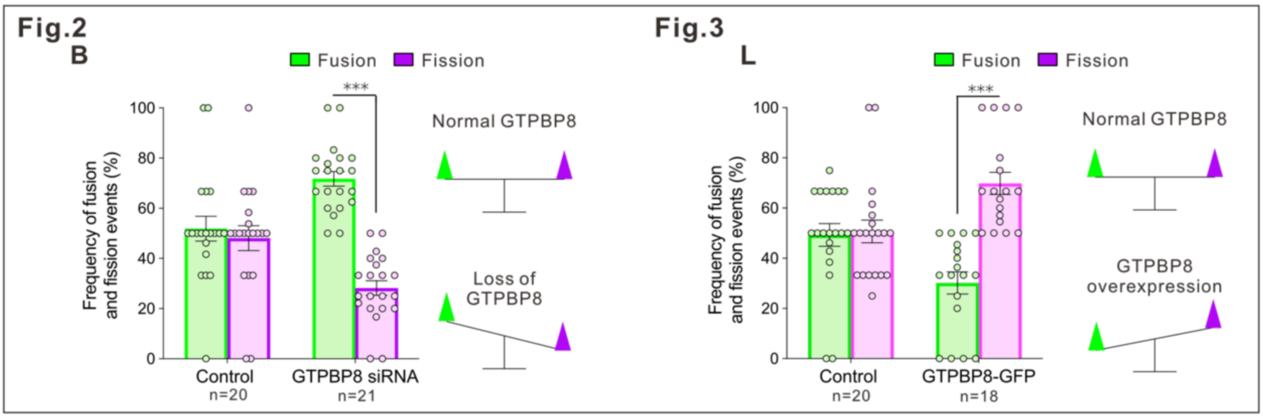

Apart from the U2OS cells, according to the reviewer's suggestion, we performed GTPBP8 knockdown and overexpression in two additional human cell lines, SKOV3 and HEK293, and the mitochondrial morphology was also quantified in the same manner. Similar to the U2OS cells, GTPBP8 knockdown in both cells resulted in an elongated mitochondrial morphology (**Fig. 1A-C**), while its overexpression led to a fragmented mitochondrial morphology compared to the tubular mitochondria in the corresponding control cells (**Fig. S3C and S3D-F**). These data indicate that GTPBP8 is involved in mitochondrial dynamics in a variety of cells.

© 2024. Published by The Company of Biologists under the terms of the Creative Commons Attribution License (https://creativecommons.org/licenses/by/4.0/).

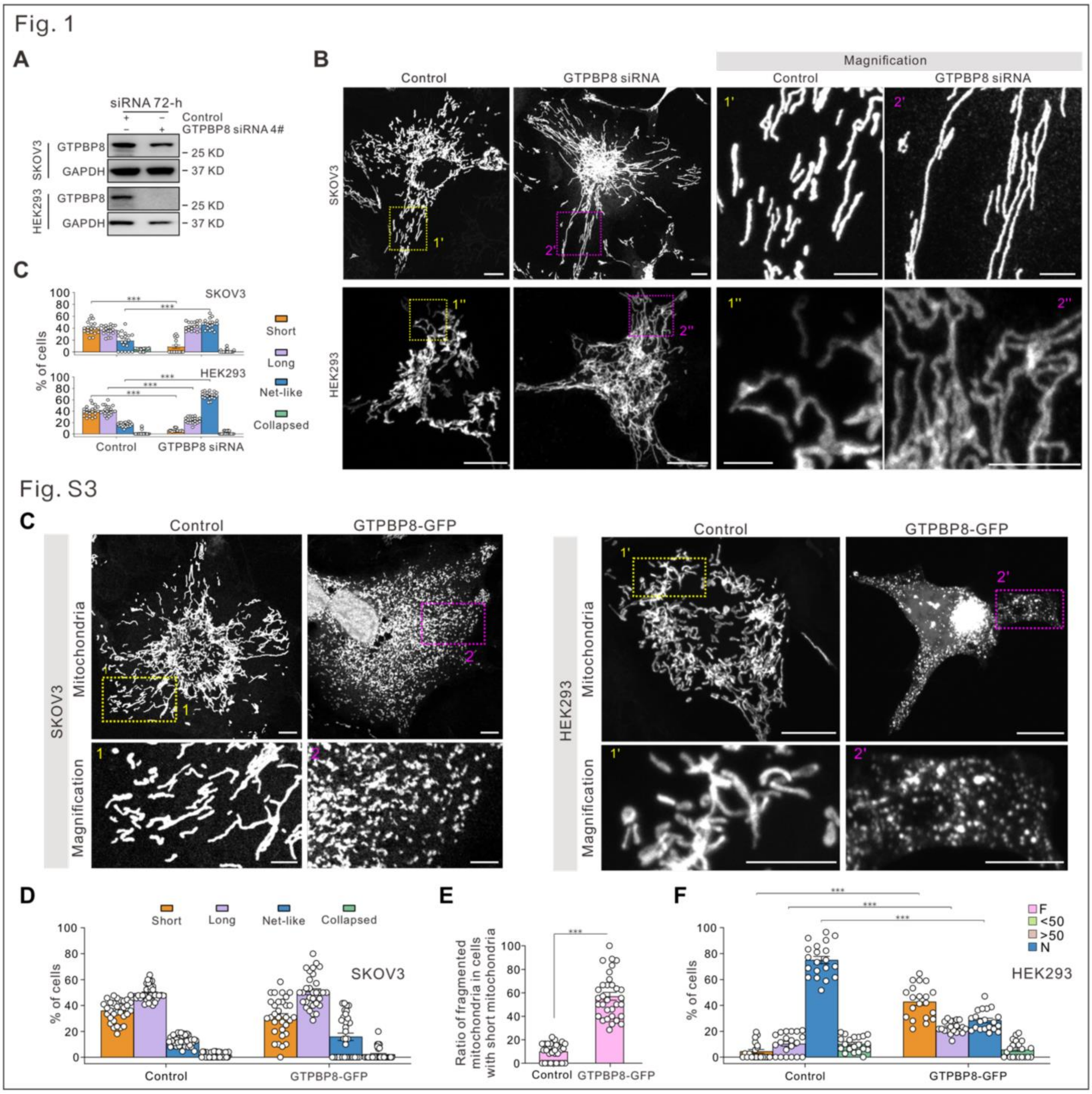

3. Is it possible that GTPBP8 knockdown is leading to a generic stress response due to reduced mitochondrial translation efficiency? For example, knockdown of LRPPRC, which is involved in mitochondrial transcription, was demonstrated to lead to mitochondrial hyperfusion as a compensatory response to improve respiratory activity (Rolland et al, PNAS, 2013). Does modulation of the mitoribosome by altering expression of other mitoribosomal assembly factors also correlate with the same effects on mitochondrial morphology?

Reply: We thank the reviewer for bringing up this insightful point. Knockdown of GTPBP8 increased oxidative stress, impaired oxidative phosphorylation, and led to mitoribosomal assembly and protein translation defects. These changes initiate the fission-signaling pathway by upregulating the level of PKA and phosphorylation of Drp1 at S637, along with changes in Drp1 localization at the MOM (**Fig. 5B**).

To address the question of whether the elongated mitochondrial morphology observed in GTPBP8 knockdown cells is a general phenomenon for cells undergoing mitochondrial translation deficiency, we silenced three other mitoribosomal assembly factors, GTPBP5 (Maiti et al., 2020), GTPBP7 (Kotani et al., 2013), and GTPBP10 (Lavdovskaia et al., 2018), known to be necessary for the assembly and maturation of mtLSU. The results showed that only GTPBP5 knockdown resulted in net-like mitochondrial morphology, whereas GTPBP7 and GTPBP10 knockdown were unable to induce the hyperfusion of mitochondria, as GTPBP8 does (**Fig. S2A-D**). These data suggest that not

© 2024. Published by The Company of Biologists under the terms of the Creative Commons Attribution License (https://creativecommons.org/licenses/by/4.0/).

all cells in a low-energy state present elongated or interconnected mitochondrial morphology. Although loss of GTPBP5 led to hyperfused mitochondrial morphology, cells with exogenously expressed GTPBP5::FLAG still present tubular mitochondrial morphology as in the non-transfected cells (Cipullo et al., 2021a). Moreover, we observed that a small proportion of GTPBP7 knockdown cells even displayed fragmented mitochondrial morphology (**Fig. S2C and S2D**). Interestingly, GTPBP8 was up-regulated upon the loss of GTPBP7, while there was less alteration in the GTPBP5 and GTPBP10 knockdown cells (**Fig. S2C**), indicating that the fragmentation of mitochondria found in the GTPBP7 knockdown cells might be related to the increased level of GTPBP8.

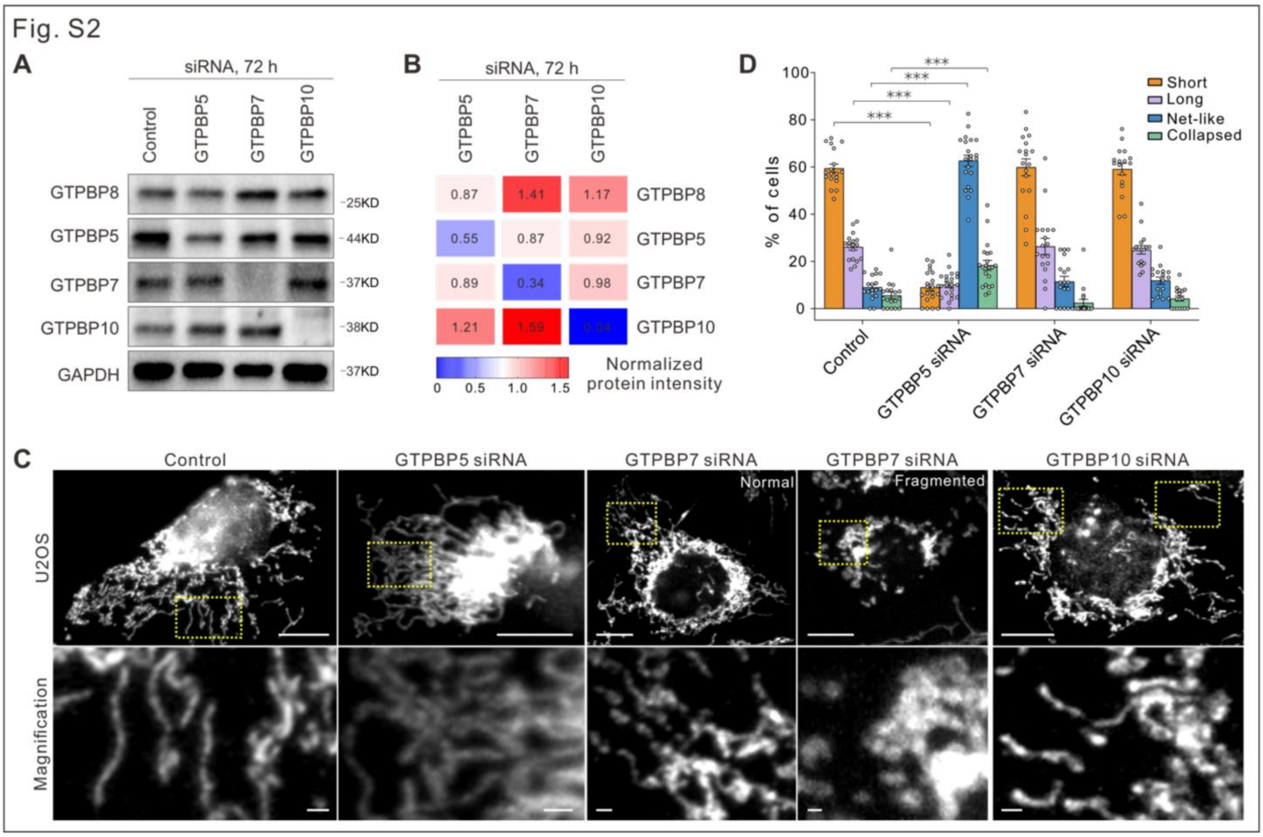

Similar research investigates the alteration in mitochondrial morphology upon the functionality inhibition of OXPHOS complexes I, III, and V and finds distinct mitochondrial morphologies. They report that the reduction of mitochondrial ATP production and oxygen consumption rate using complexes I, III, and V inhibitors, rotenone, antimycin, and oligomycin, respectively, caused different mitochondrial morphologies. The inhibition of complex I does not result in a shift in the identified mitochondrial populations. The inhibition of complex V induces an increase in fragmented mitochondria. While the inhibition of complex V results in a slight decrease in fragmented mitochondria (van der Stel et al., 2023). Collectively, these data suggest that not all cells in the low-energy state will present hyperfusion mitochondrial morphology.

*****

Reviewer 2 Advance Summary and Potential Significance to Field:

GTPBP8 is a newly identified GTP-binding protein, whose discovery by this very group is currently in BioRxiv (https://www.biorxiv.org/content/10.1101/2023.05.26.542385v2.full.pdf). In this BioRxiv, GTPBP8 showed low intrinsic GTPase activity and localization in the mitochondria, specifically the matrix with some IMM interaction. Depletion of GTPBP8 (siRNA) diminished oxygen consumption rates in a standard Seahorse Assay (basal, ETC leak, maximal, and non-mitochondrial OCR). Moreover, protein levels of subunits in Complex I, III, IV, and the ATP synthase were reduced, suggestive of reduced mitochondrial energy production. The exclusion of Complex II led the authors to hypothesize/test a potential role in mitoribosome function, however it's unclear if mtDNA maintenance/propagation is also important in the process.

© 2024. Published by The Company of Biologists under the terms of the Creative Commons Attribution License (https://creativecommons.org/licenses/by/4.0/).

Reply: We thank the reviewer for the thoughtful comments. In response, we further examined the effects of GTPBP8 depletion on the localization and copy number of mtDNA, mitochondrial membrane potential, and the production of cellular reactive oxygen species. In contrast to control cells, GTPBP8 knockdown caused a significant increase in oxidative stress but had no effect on mitochondrial membrane potential, the copy number and localization of mtDNA (**Fig. 1I-K**). The bioenergetic deficit in GTPBP8-depleted U2OS cells was mainly attributed to defects in mitochondrial translation rather than impaired mtDNA content or decreased transcription levels (Wang et al., 2023). These data indicate that mtDNA was maintained in a steady state in GTPBP8-depleted cells.

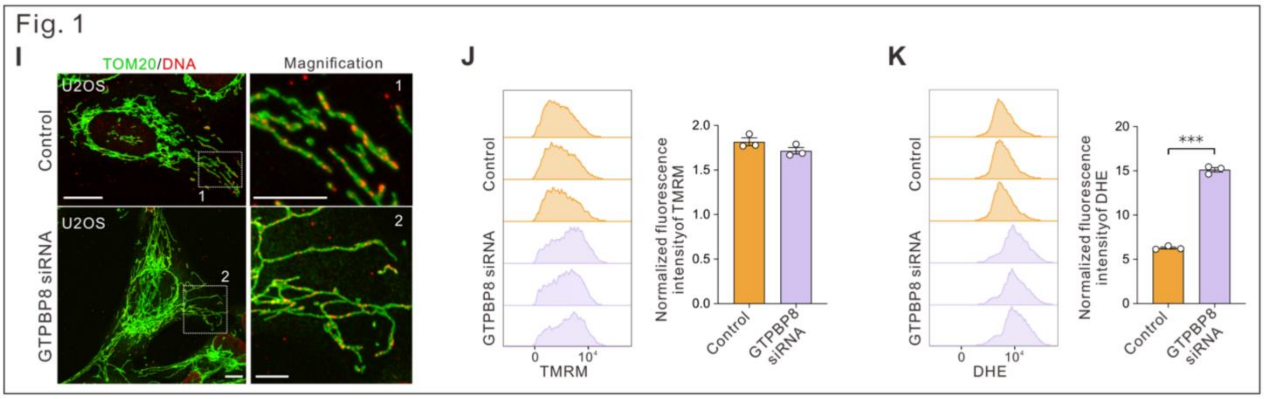

In this study, He et al. study the relationship between GTPBP8 and mitochondrial fusion/fission. The authors present clear and consistent evidence that knockdown or overexpression of GTPBP8 is well correlated with fused mitochondria and fragmented mitochondria, respectively. The authors show this is dependent on Drp1, with the phosphorylation status of Drp1 as an important variable of fission/fusion. The study is well written, polished, figures are clear, and presents a consistent story. However, there is little mechanistic insight into how mitochondrial GTPBP8 regulates cytosolic Drp1, and most importantly if this pathway is a direct mechanism or a consequence of a mitochondrial energetic disturbances, as has been demonstrated for small molecule Honokiol (https://www.ncbi.nlm.nih.gov/pmc/articles/PMC5512068IF: 4.8 Q2 /).

Reply: We thank the reviewer for bringing up this insightful point. Knockdown of GTPBP8 caused mitoribosomal assembly and protein translation defects, eventually leading to increased oxidative stress and impaired oxidative phosphorylation. These changes initiate the fission-signaling pathway by upregulating phosphorylation of Drp1 at S637, along with changes in Drp1 localization at the MOM (**Fig. 5A and 5D**). Drp1 activity is dependent on its activation through various post-translational modifications, such as phosphorylation and SUMOylation (Figueroa- Romero et al., 2009; Xu et al., 2016a). Phosphorylation of Drp1 at Ser616 can be modulated by different protein kinases involved in distinct signaling pathways, including protein kinase C (PKC), CDK1/Cyclin B, or ROCK, to promote fission (Lima et al., 2018; Han et al., 2008). Conversely, phosphorylation of Drp1 at Ser637 by protein kinase A (PKA) inhibits fission (Han et al., 2008). To determine whether the up-regulated Drp1 S637-PO4 level in GTPBP8- depleted cells was regulated by those kinases, we examined the expression levels of CDK1, Cyclin B, ROCK1, ERK, and PKA. As shown in **Fig. 5A and Fig. S5C**, the level of PKA was up-regulated, while the levels of CDK1 and Cyclin B were down-regulated after silencing GTPBP8 in U2OS cells. The expression levels of ROCK1 and ERK were unaffected following the depletion of GTPBP8. Conversely, GTPBP8 overexpression resulted in decreased PKA and increased Cyclin B (**Fig. 5D**). Together, our new data show that the changed mitochondrial morphology and dynamics in response to the altered GTPBP8 level is mainly attributed to the modified phosphorylation state of Drp1 by regulating PKA and CDK1/Cyclin B.

© 2024. Published by The Company of Biologists under the terms of the Creative Commons Attribution License (https://creativecommons.org/licenses/by/4.0/).

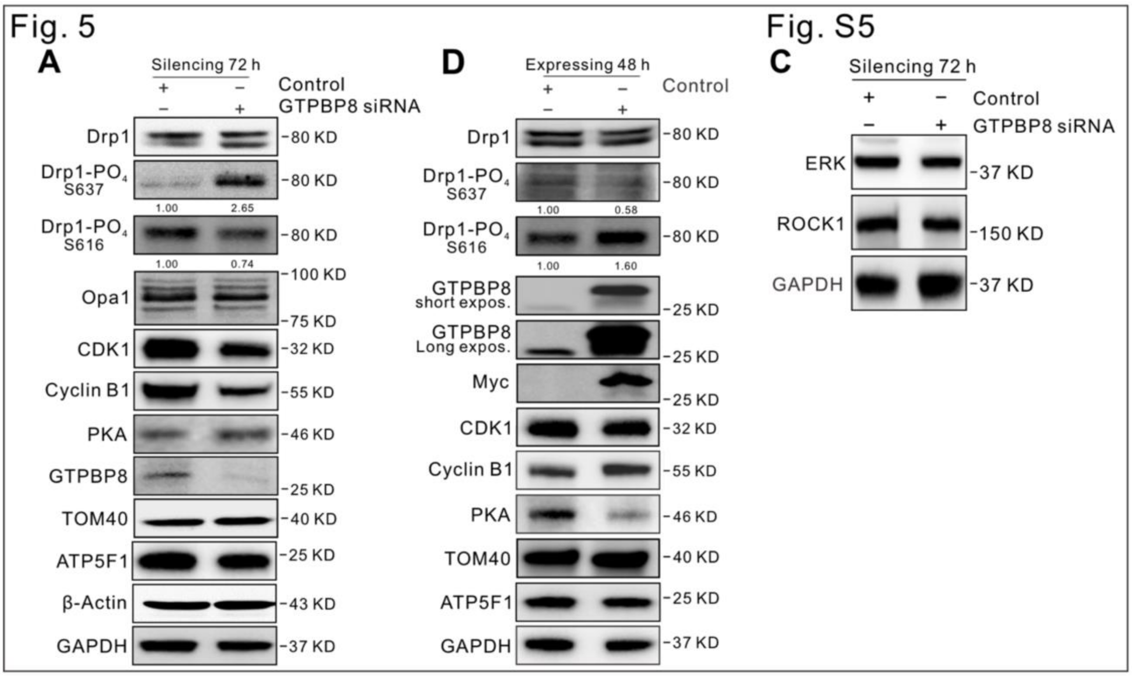

Collectively, these data point to the possibility that GTPBP8 depletion caused defects in the assembly of mtLSU, resulting in compromised mitochondrial translation and, consequently, impaired mitochondrial function. This mitochondrial dysfunction could further initiate cell signaling involving in the phosphorylation of Drp1 at Ser637, the reduced recruiting of Drp1 to mitochondria, and eventually decreased mitochondrial fission.

Reviewer 2 Comments for the Author:

Given that there's no mechanism connecting GTPBP8 to Drp1, and this process is entirely dependent on traditional OMM fusion/fission machinery, the authors should be more conservative in their conclusive statements. For example, a more accurate title would be "GTPBP8 regulates mitochondrial fission through a Drp1-dependent process". In the concluding statements, it's not clear if 'GTPBP8 and Drp1 collaborate in orchestrating mitochondrial fission' or if GTPBP8, and the consequences of its knockdown/overexpression, are causing mitochondrial stress, which is changing Drp1 dependent mitochondrial dynamics.
Reply: We thank the reviewer for the constructive comments and suggestions. Following the recommendations, the title of the manuscript has been changed to "GTPBP8 Regulates Mitochondrial Fission through a Drp1-Dependent Process". Moreover, we have conducted additional experiments to address the reviewer's major concerns regarding the altered mitochondrial morphology in cells with modified GTPBP8 levels. We believe that addressing these concerns has significantly improved the quality of our study, and we hope that our response sufficiently addresses the reviewer's concern.

In brief, both the mitochondrial stress and cell signaling involved in the phosphorylation of Drp1 in the GTPBP8 knockdown cells have been investigated in the revised manuscript. These data point to the possibility that GTPBP8 depletion caused defects in the assembly of mtLSU, resulting in compromised mitochondrial translation and, consequently, impaired mitochondrial function. This mitochondrial dysfunction might further regulate cell signaling involved in the phosphorylation of Drp1, leading to the increased phosphorylation of Drp1 at Ser637, reduced recruiting of Drp1 to mitochondria, and eventually decreased mitochondrial fission.

There are few concerns with the core findings (GTPBP8 expression is correlated with fusion/fission of mitochondria) as they are well supported. The following major and minor comments should still be addressed:
Major:
1) Figure 1C: how were cells/mitochondria scored? Was it automated, manual, and what were the criteria for designating short, long, net, etc? The authors cite a prior study, but some description of the methods would be useful.

© 2024. Published by The Company of Biologists under the terms of the Creative Commons Attribution License (https://creativecommons.org/licenses/by/4.0/).

Reply: We appreciate the reviewer's valuable comments, and we have incorporated a detailed description of the analysis into the method section in the revised manuscript. The mitochondrial morphology in GTPBP8-deficient cells was manually scored, categorizing them into four types: short tubules, long tubules, net-like structures, or collapsed (**Fig. S1B**):

1) Short tubules: more than 50% of mitochondria in a single cell exhibit short tubules.
2) Long tubules: more than 50% of mitochondria in a single cell exhibit long tubules.
3) Net-like structures: most of the mitochondria in a single cell are interconnected, with few or no long tubules.
4) Collapsed: most of the mitochondria were highly condensed and accumulated around the nucleus, with few or no individual mitochondria around the cell membrane.

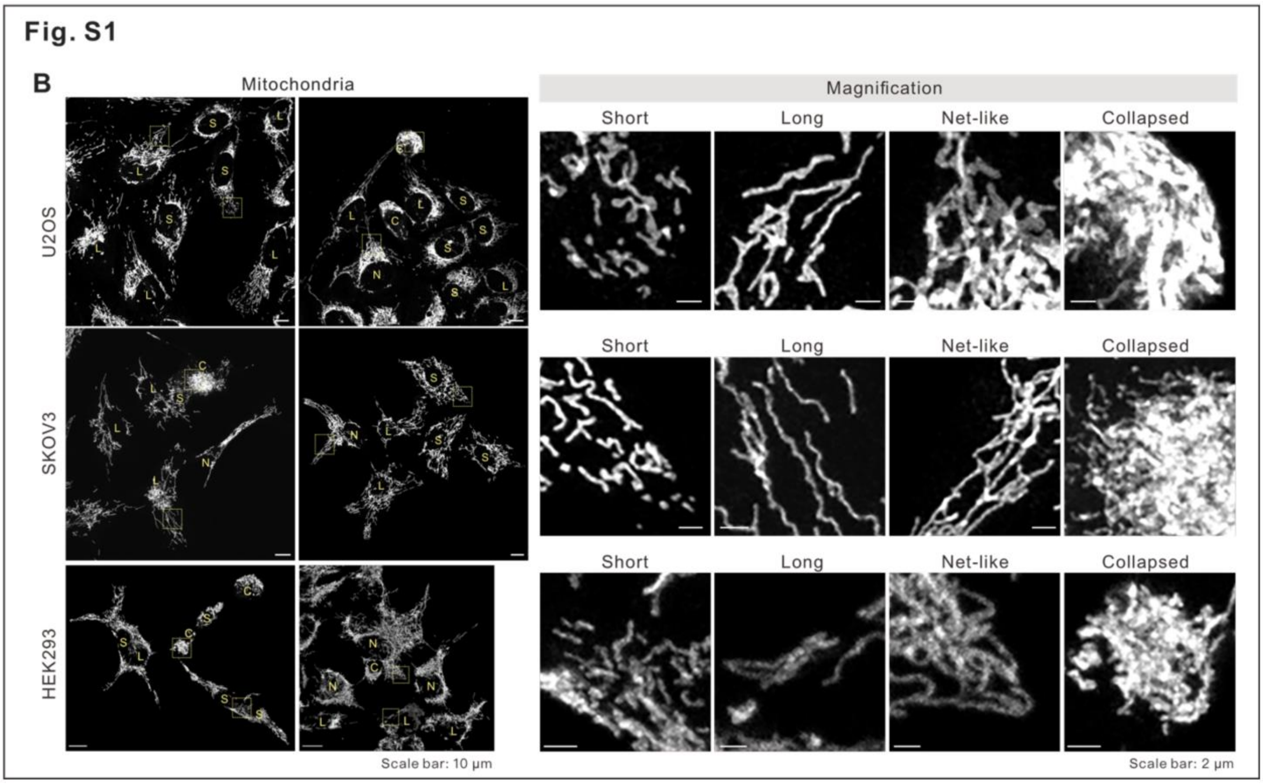

The mitochondrial morphology in the GTPBP8-expressed cells was also manually scored into four categories: fragmented (F), <50, >50, or net-like structures (**Fig. S3A**):

1) F: almost all mitochondria in a single cell exhibit dot-like morphology.
2) <50: less than 50% of mitochondria in a single cell exhibit long tubules.
3) >50: more than 50% of mitochondria in a single cell exhibit long tubules.
4) Net-like structures: most of the mitochondria in a single cell are interconnected, with few or no long tubules.

The ratio of each kind of mitochondrial morphology was generated by dividing the total number of cells in one category of mitochondria by the total number of cells in the microscopic field. To easily understand the criteria for the classification of mitochondrial morphology. We have supplemented representative images for each kind of mitochondrial morphology into the supplemental figures (**Fig. S1B and S3A**). We also added this mitochondrial morphometric analysis in the methods section of the revised manuscript (**Page 19, Line 504-519**).

© 2024. Published by The Company of Biologists under the terms of the Creative Commons Attribution License (https://creativecommons.org/licenses/by/4.0/).

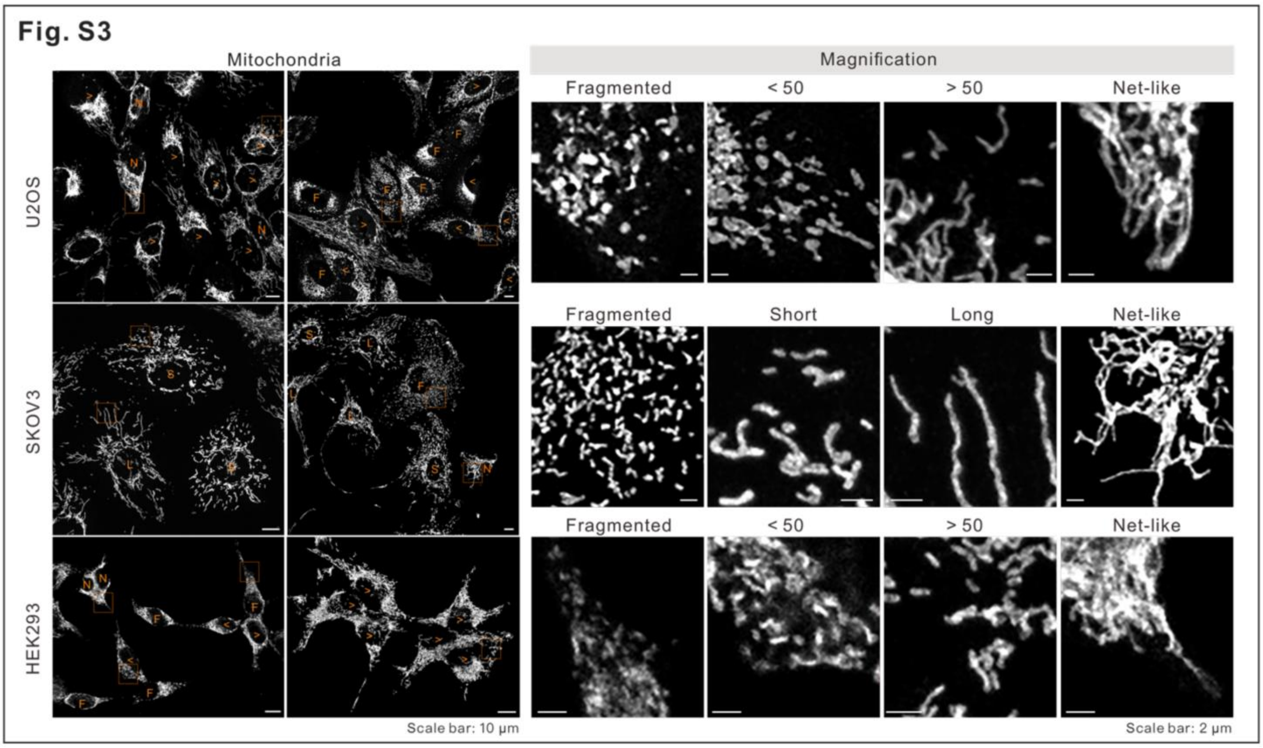

2) “However, no discernible alterations were observed in mitochondrial architecture following GTPBP8 knockdown. Similar to the control group, the outer membrane retained its smooth appearance enveloping the mitochondria, the inner membrane displayed extensive folding characterized by numerous cristae, and the cristae themselves maintained their characteristic tubule- or sheet-like structures, even in the absence of GTPBP8 (Fig. 1D).” Based on the images provided, this does not appear to be true. The cristae are more pronounced, numerous, and perpendicular to the membrane in control. If this is due to contrast/zoom, please provide different images. However, if these images were misinterpreted, reduced cristae would also support the conclusions of their prior study (decreased OCR, decreased ETC protein, etc).

Reply: We appreciate the reviewer's attention to detail and thoughtful suggestions. In response, we quantified the number of cristae and measured the corresponding mitochondrial area using electron micrographs. As observed by the reviewer, GTPBP8 deficiency led to a reduced crista density compared to control cells (**Fig. S1D**). Despite this decrease, the lamellar or tubular crista morphology remained unaffected following GTPBP8 knockdown. We have incorporated these new data into the revised manuscript and included additional electron micrographs of mitochondria in the supplemental figures (**Fig. S1C**).

© 2024. Published by The Company of Biologists under the terms of the Creative Commons Attribution License (https://creativecommons.org/licenses/by/4.0/).

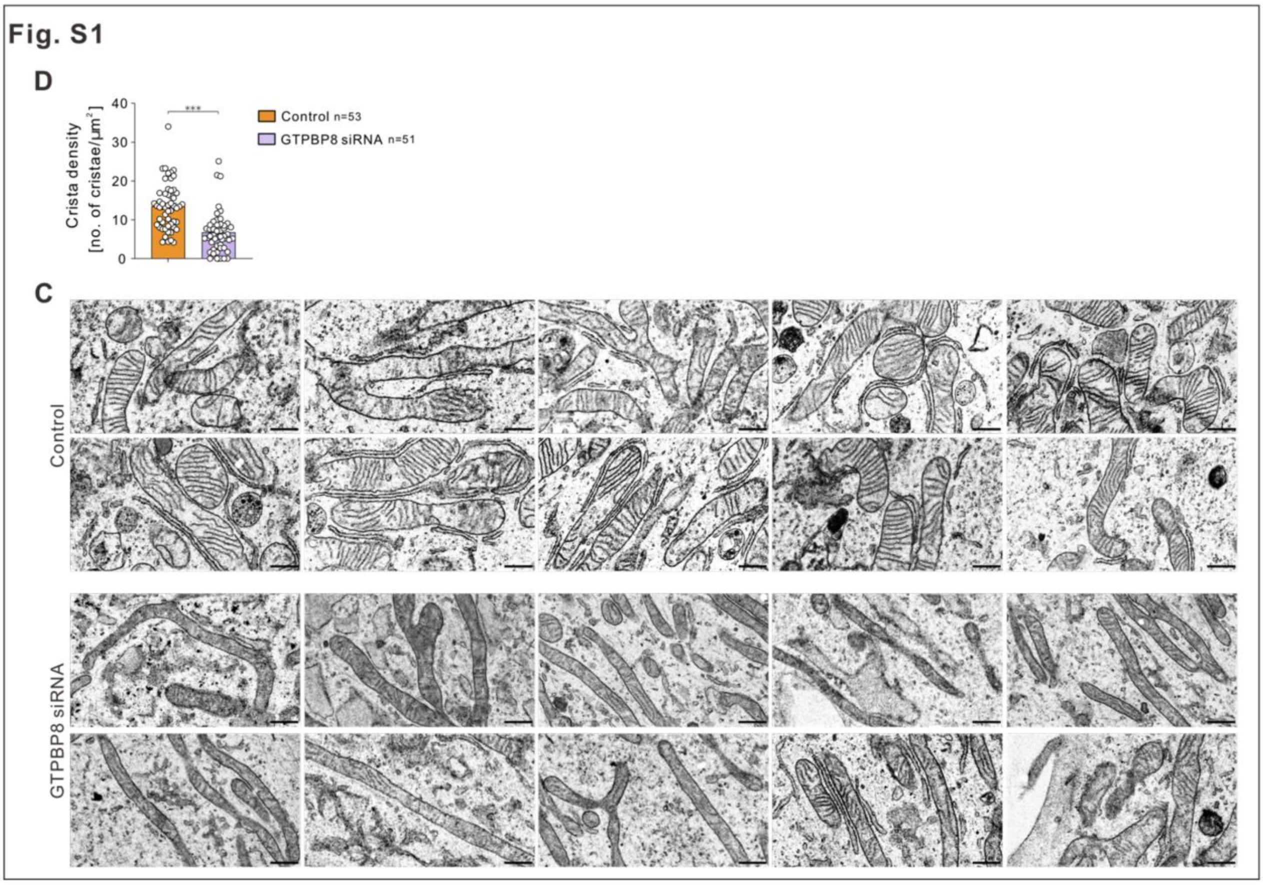

3) "The occurrences of fission and fusion events within a fixed time frame were quantified. In comparison to control cells, GTPBP8 knockdown led to a reduction in mitochondrial fission events and an increase in fusion events (Fig. 2A)." The authors present images where it appears there are 3 fission events and 1 fusion event in control vs. 2 fission events and 1 fusion event in KD. Did the authors intend to include a larger sample size plot where statistical confidence can be more easily interpreted? Similar comments for Figure 3K, where statistical power is low and similar concerns arise.

Reply: We appreciate the reviewer's valuable comments. We manually counted the fission and fusion events within a fixed 10 minutes of mitochondrial tracking. By counting the number of fission events and fusion events in 10 minutes, the ratio of fission and fusion events was calculated as follows:

Ratio of fission = The total number of fission events / the total number of fission and fusion events *100

Ratio of fusion = The total number of fusion events / the total number of fission and fusion events *100

Compared to the balanced fission and fusion in the control cells, the frequency of fission events was reduced after silencing GTPBP8 in U2OS cells (**Fig. 2B**). Conversely, GTPBP8 overexpression resulted in an increased frequency of mitochondrial fission (**Fig. 3L**). These data suggest that the elongated or fragmented mitochondrial morphology in the GTPBP8 dysregulated cells was mainly attributed to an imbalanced mitochondrial fusion and fission but not a loss of fission or fusion capabilities.

© 2024. Published by The Company of Biologists under the terms of the Creative Commons Attribution License (https://creativecommons.org/licenses/by/4.0/).

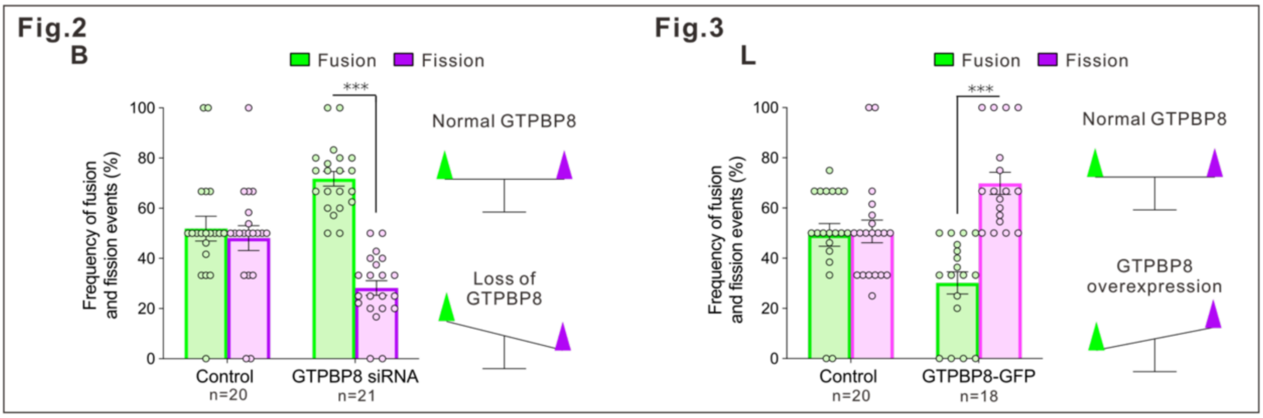

4) Fig S1D: Please explain why there are two bands in the Drp1 KD and why one appears reduced whereas the other increased for the siRNA lane.

Reply: We appreciate the reviewer's critical comments. To acquire the cells with the maximum Drp1-depleted level, we ordered new Drp1 siRNA reported in the article (Lee et al., 2004) and repeated the Drp1 knockdown experiments in U2OS cells. The immunoblots of Drp1 showed that both bands are affected after treating cells with Drp1 siRNA for 72 h (**Fig. S5A**). After treating the U2OS cells with Drp1 siRNA for 24 h, GTPBP8-GFP was subsequently overexpressed in Drp1-depleted cells for 48 h. The immunofluorescent images show that GFPBP8-GFP failed to induce mitochondrial fragmentation following the knockdown of Drp1 in U2OS cells (**Fig. 4A and 4B**). The updated data, including the Drp1 protein level after treatment with the Drp1 siRNA for 72 h and the mitochondrial morphology following a 48-h overexpression of GTPBP8-GFP in Drp1-depleted cells, have been added to the revised figures (**Fig. 4A, 4B and Fig. S5A and S5B**).

© 2024. Published by The Company of Biologists under the terms of the Creative Commons Attribution License (https://creativecommons.org/licenses/by/4.0/).

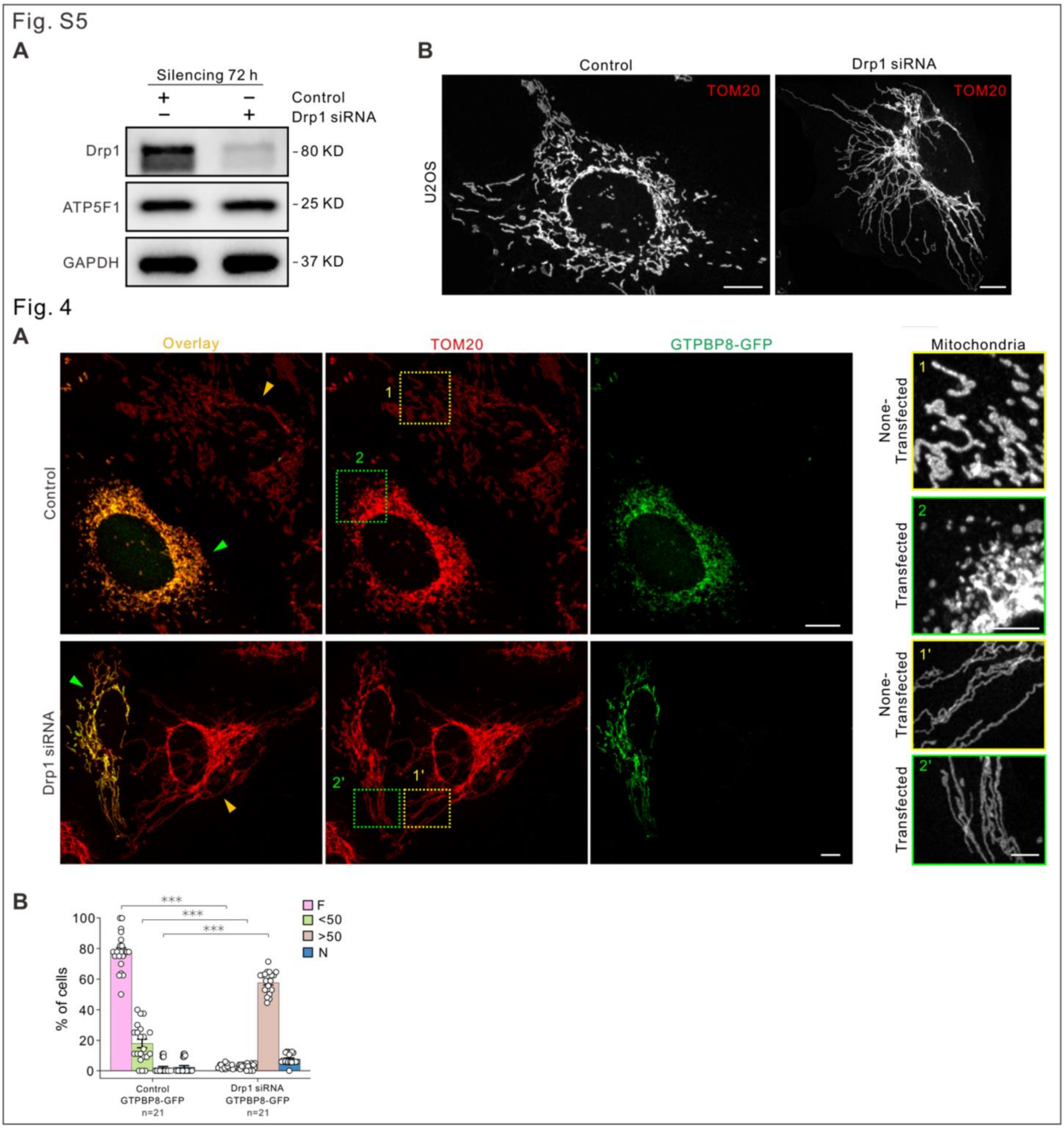

5) Figure 5D, Drp1-PO4: the effects are so small compared to the 5A KDs that it is difficult to appreciate the author's point. Do the authors have any other blots for this figure? For example, more sample to increase contrast?

Reply: Following the reviewer's suggestions, we repeated the experiments by increasing the amount of protein. Both Drp1-PO4 S616 and Drp1-PO4 S637 were detected after silencing GTPBP8 or overexpressing GTPBP8-Myc in U2OS cells, respectively. The updated blot images show a significant improvement in the band of Drp1-PO4 S616, while the band of Drp1-PO4 S637 remains similar to the previously detected level. The results rely largely on the quality of antibodies, particularly the antibodies for phosphorylated proteins. We have replaced the Drp1-PO4 S616 in both **Fig. 5A and 5D**.

© 2024. Published by The Company of Biologists under the terms of the Creative Commons Attribution License (https://creativecommons.org/licenses/by/4.0/).

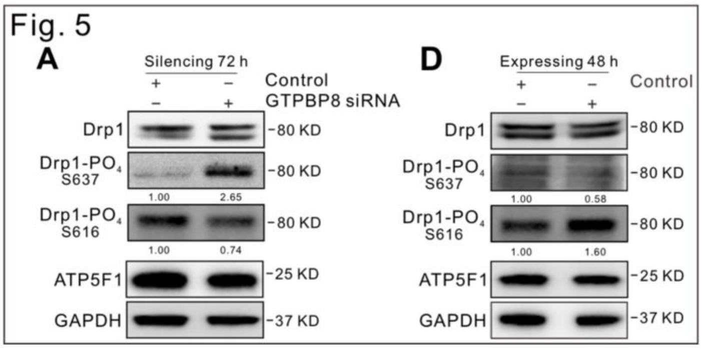

Minor:

1) Line 59: specific which membrane or indicate both membranes.

Reply: We appreciate the reviewer's careful check and apologize for the incomplete description. The previous sentence has been changed in the revised manuscript: 'other fission factors located on the mitochondrial outer membrane' (**Page 3, Line 60**).

2) Methods: include a table summarizing antibodies with a column dedicated to 'knockout verified'. **Reply:** Following the reviewer's suggestion, we have compiled all antibodies used in this study into a table. Additionally, details on the 'knockout verification' have been provided in the **Table S1** of the revised manuscript.

Antibodies	Source	Identifer	Knockout verification	Application
Rabbit anti-GTPBP8	Sigma	HPA034831	This lab	WB: 1:500
Rabbit anti-Drp1 S616-PO_4	Cell Signaling Technology	#4494	N/A	WB: 1:1000
Rabbit anti-Drp1 S637-PO_4	Cell Signaling Technology	#6319	N/A	WB: 1:1000
Mouse anti-Drp1	Cell Signaling Technology	#14647	N/A	WB: 1:1000<br>IF: 1:100
Mouse anti-Opa1	BD Biosciences	612606	N/A	WB: 1:10000
Mouse anti-Myc	Santa Cruz Biotechnology	sc-40	N/A	WB: 1:1000
Mouse anti-DNA	Millipore Sigma	CBL186	N/A	IF: 1:250
Mouse anti-TOM20	Santa Cruz Biotechnology	sc-17764	N/A	WB: 1:1000<br>IF: 1:200
Rabbit anti-PKA	Proteintech	55382-1-AP	N/A	WB: 1:1000
Rabbit anti-CDK1	Proteintech	19532-1-AP	N/A	WB: 1:10000
Rabbit anti-Cyclin B1	Proteintech	55004-1-AP	N/A	WB: 1:4000
Rabbit anti-ROCK1	Proteintech	21850-1-AP	N/A	WB: 1:1000
Rabbit anti-ERK	Cell Signaling Technology	#4695S	N/A	WB: 1:1000
Mouse anti-ATP5F1	Santa Cruz Biotechnology	sc-514419	N/A	WB: 1:500
Mouse anti-TOM40	Santa Cruz Biotechnology	sc-365466	N/A	WB: 1:1000
Rabbit anti-COXIV	ThermoFisher Scientific	MA5-15078	N/A	IF: 1:200
Rabbit anti-α-Tubulin	Proteintech	66031-1-Ig	N/A	WB: 1:20000
Rabbit anti-β-Actin	Proteintech	81115-1-RR	N/A	WB: 1:50000
Rabbit anti-GAPDH	Yeasen Biotechnology	30202ES60	N/A	WB: 1:10000
WB-Western blot, IF- Immunofluorescence				

3) Figure 1D: To compare mitochondria, it would be helpful to have similar magnification/zoom on the images.

Reply: We appreciate the thoughtful suggestion from the reviewer. Following their advice, we have revised the electron micrographs in **Fig. 1D and 3F** to the same scale size. Additionally, we have included more electron micrographs of mitochondria with the same scale size in the supplemental figures (**Fig. S1C and S4**) to further illustrate the changes in mitochondrial structure.

© 2024. Published by The Company of Biologists under the terms of the Creative Commons Attribution License (https://creativecommons.org/licenses/by/4.0/).

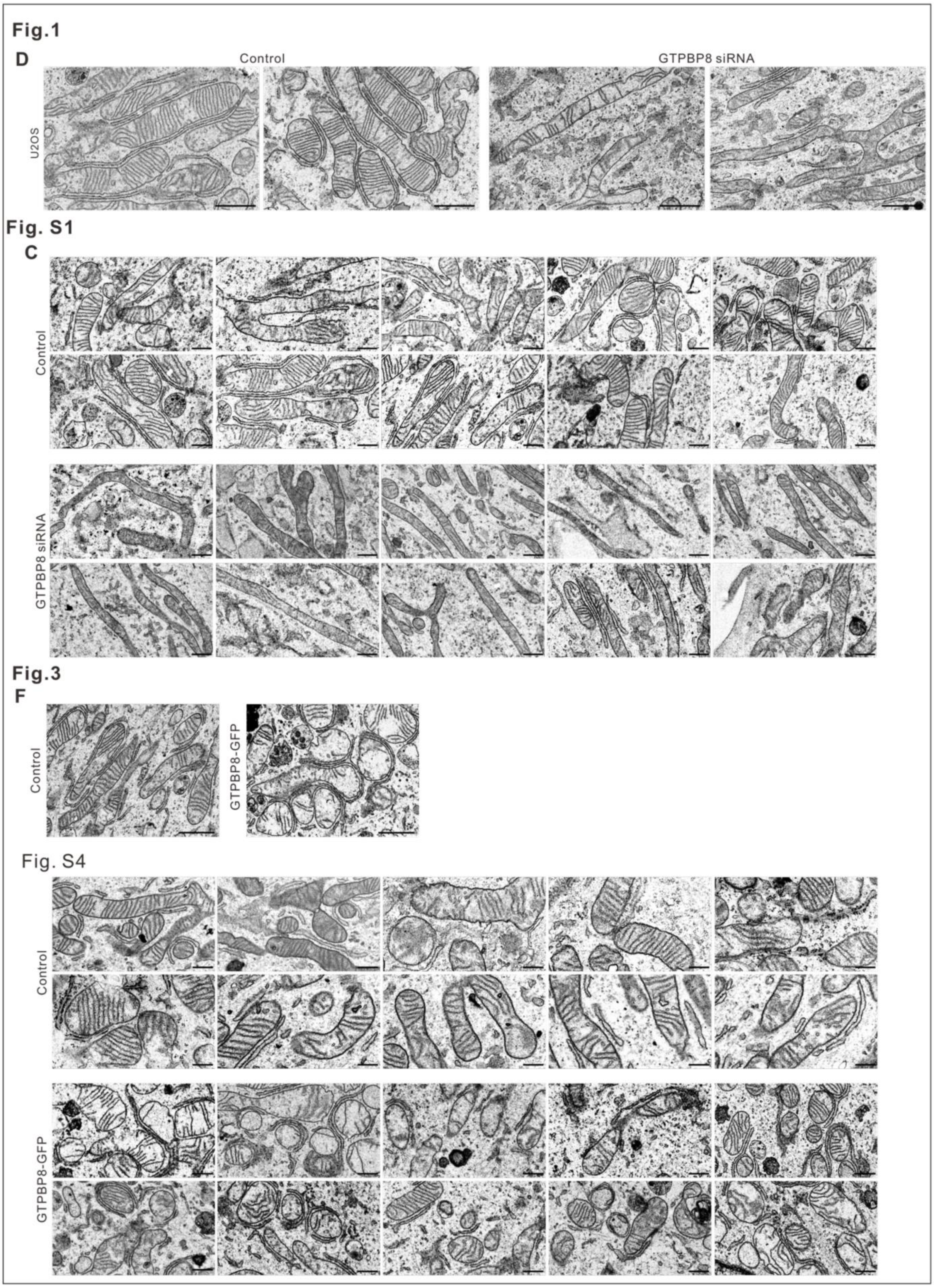

4) Figure 1E/F/G: significant digits. Based on the scalebar of 500 nm and axes in Fig. 1H, it's unlikely analysis from this plot is accurate to 3 decimal places from um (single nm). Note that Fig. 1G extends to 0.008302 um; accuracy to single pm. The authors should correct their mean/error mathematics in the text such that decimal places reflect the limitations of the technique. Similar statistics are presented in the text for Figure 3, where decimal places extend to

© 2024. Published by The Company of Biologists under the terms of the Creative Commons Attribution License (https://creativecommons.org/licenses/by/4.0/).

single nm or pm, pending the panel.
Reply: We appreciate the reviewer's critical comments, and we agree with their point. To avoid any confusion, the original description of mitochondrial width in Fig. 1G and Fig. 3I has been revised (Fig. 1G, **Page 7, Line 150-151;** Fig. 3I, **Page 10, Line 242-243**) in the manuscript. The previous description of the alteration in mitochondrial width was based on statistical analysis (Average mean width ± Std. Error of Mean). The value of 0.008302 was not a measured value but a calculated standard deviation of the mean (SEM).

5) Fig 5A: Similar to S1D, the authors should explain why there are two Drp1 bands and why they are most interested in the change to the lower band, as the lower band was largely unaffected by Drp1 KD.
Reply: We appreciate the reviewer for bringing this to our attention. Our data indicate the presence of two Drp1 bands when using the commercial monoclonal anti-Drp1 antibody (#14647, Cell Signaling Technology), consistent with the manufacturer's documentation (https://www.cellsignal.com/products/primary-antibodies/drp1-4e11b11-mouse- mab/14647) and as observed in other published papers (Valera-Alberni et al., 2021; Xu et al., 2016a). To be honest, based on our examination of the available literature, we find that Drp1 immunoblots occasionally show two bands and occasionally just one band, even within the same article. However, there is no explanation for the difference between those two bands. We guess these two bands could be the different forms of Drp1 in cells, for example, post- translational modification of Drp1. To acquire the cells with the maximum Drp1-depleted level, we ordered new Drp1 siRNA reported in the article (Lee et al., 2004) and repeated the Drp1 knockdown experiments in U2OS cells. The immunoblots of Drp1 showed that both bands are affected by Drp1-silencing (**Fig. S5A**).

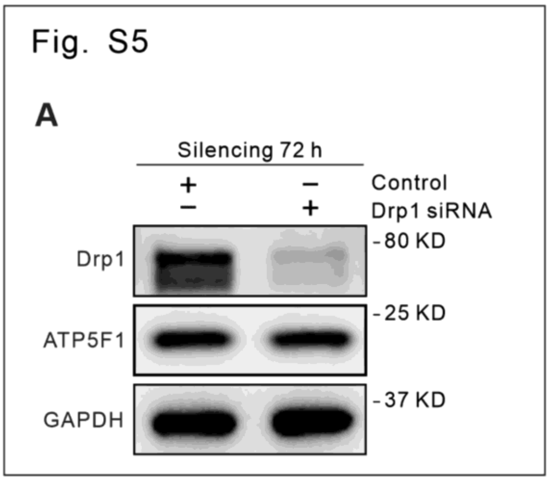

Reviewer 3 Advance Summary and Potential Significance to Field:
The current manuscript "GTPBP8 Regulates Mitochondrial Fission through Drp1" by He et al explores the role of the mitochondrial localized GTPase GTPBP8 on mitochondrial dynamics. This paper does a good job of showing that up and downregulation of GTPBP8 has complementary effects on mitochondrial dynamics, where GTPBP8 knockdown causes mitochondrial elongation and GTPBP8 overexpression causes mitochondrial fragmentation. They are further able to connect alterations in GTPBP8 expression with changes in Drp1 phosphorylation. They also showed that knockdown of Drp1 rescued the mitochondrial fragmentation phenotype of GTPBP8 overexpression. These data point to a role of GTPBP8 in mitochondrial function and the need to further investigate the mechanism by which it impacts Drp1 to influence mitochondrial dynamics.

Reviewer 3 Comments for the Author:

My enthusiasm is somewhat tempered because it is unclear how the expression of the inner mitochondrial membrane protein GTPBP8 protein is able to impact the phosphorylation of the cytosolic protein Drp1 and therefore mitochondrial fission. I believe the manuscript could be improved by changing how the manuscript is currently framed as at multiple points the paper is written implying that they hope to find mechanism of fission at the inner membrane. In the abstract

© 2024. Published by The Company of Biologists under the terms of the Creative Commons Attribution License (https://creativecommons.org/licenses/by/4.0/).

they state “our understanding of the fission mechanisms within the inner mitochondrial membrane and the associated factors/proteins is limited.” Similarly on line 308 the authors state “the precise coordination between outer and inner membrane fission events and the factors involved are yet to be defined.” But to me there is a distinction between “fission mechanisms” and “fission signaling”. Fission mechanisms implies factors that constrict the mitochondria to actually drive scission of the inner and outer mitochondrial membrane. While fission signaling does not need to be directly involved in the fission mechanism or scission of the mitochondria but instead starts a cascade to impact the actual fission machinery. Mitochondrial function is a general fission signaling mechanism as loss of membrane potential can drive mitochondrial fission. Could GTPBP8 just be generally impacting mitochondrial function and that secondarily impacts fission and fusion? While the authors cite another bioRxiv paper from their lab that GTPBP8 is an inner mitochondrial membrane protein they do not present any data that GTPBP8 is a direct fission or fusion factor.

Reply: We appreciate the reviewer's critical comments and suggestions. We cannot agree more with the reviewer’s comments about the fission signaling. Following the recommendation, the title of the manuscript has been changed to 'GTPBP8 Regulates Mitochondrial Fission through a Drp1-Dependent Process.' Moreover, we conducted additional experiments to address the reviewer’s major concerns regarding the altered mitochondrial morphology in cells with modified GTPBP8 levels. We believe that addressing these concerns has significantly improved the quality of our study, and we hope that our response sufficiently addresses the reviewer's concerns.

In brief, both mitochondrial stress and cell signaling involved in the phosphorylation of Drp1 in the GTPBP8 knockdown cells have been investigated in the revised manuscript. These data suggest the possibility that GTPBP8 depletion caused defects in the assembly of mtLSU, resulting in compromised mitochondrial translation and, consequently, impaired mitochondrial function. This mitochondrial dysfunction could further regulate cell signaling involved in the phosphorylation of Drp1, leading to increased phosphorylation of Drp1 at Ser637, reduced recruitment of Drp1 to mitochondria, and eventually decreased mitochondrial fission. The new results below support these notions:

1) Our work shows that GTPBP8 is a mitoribosomal assembly factor and resides in both the mitochondrial inner membrane and matrix. However, GTPBP8 interacts with MRPL16 and MRPL31, which do not localize closely to the inner mitochondrial membrane as MRPL45 does (Wang et al., 2023). The function of membrane-associated GTPBP8 remains to be elucidated. To investigate whether the role of GTPBP8 in mitoribosomal assembly is involved in mitochondrial fission signaling, we examined the changes in mitochondrial morphology after depleting three other mitoribosomal assembly factors, GTPBP5 (Maiti et al., 2020), GTPBP7 (Kotani et al., 2013), and GTPBP10 (Lavdovskaia et al., 2018). The results showed that only GTPBP5 knockdown resulted in net-like mitochondrial morphology, whereas GTPBP7 and GTPBP10 knockdown were unable to induce the hyperfusion of mitochondria (**Fig. S2A-D**). According to the reduced oxygen consumption reported in the GTPBP5-, GTPBP7-, and GTPBP10- deficient cells (Kim and Barrientos, 2018; Cipullo et al., 2021a; Maiti et al., 2018), these data suggest that not all cells in a low-energy state present elongated or interconnected mitochondrial morphology. Although loss of GTPBP5 led to hyperfused mitochondrial morphology, cells with exogenously expressed GTPBP5::FLAG still present tubular mitochondrial morphology as in the non-transfected cells (Cipullo et al., 2021a). Moreover, we observed that a small proportion of GTPBP7 knockdown cells even displayed fragmented mitochondrial morphology (**Fig. S2C and S2D**). By detecting the protein level of GTPBP8 in the GTPBP5-, GTPBP7-, and GTPBP10-knockdown cells, we found that GTPBP8 was up-regulated following the loss of GTPBP7 while with less alteration in the GTPBP5 and GTPBP10 knockdown cells (**Fig. S2B**). These data suggest the important role of GTPBP8 in regulating mitochondrial dynamics.

© 2024. Published by The Company of Biologists under the terms of the Creative Commons Attribution License (https://creativecommons.org/licenses/by/4.0/).

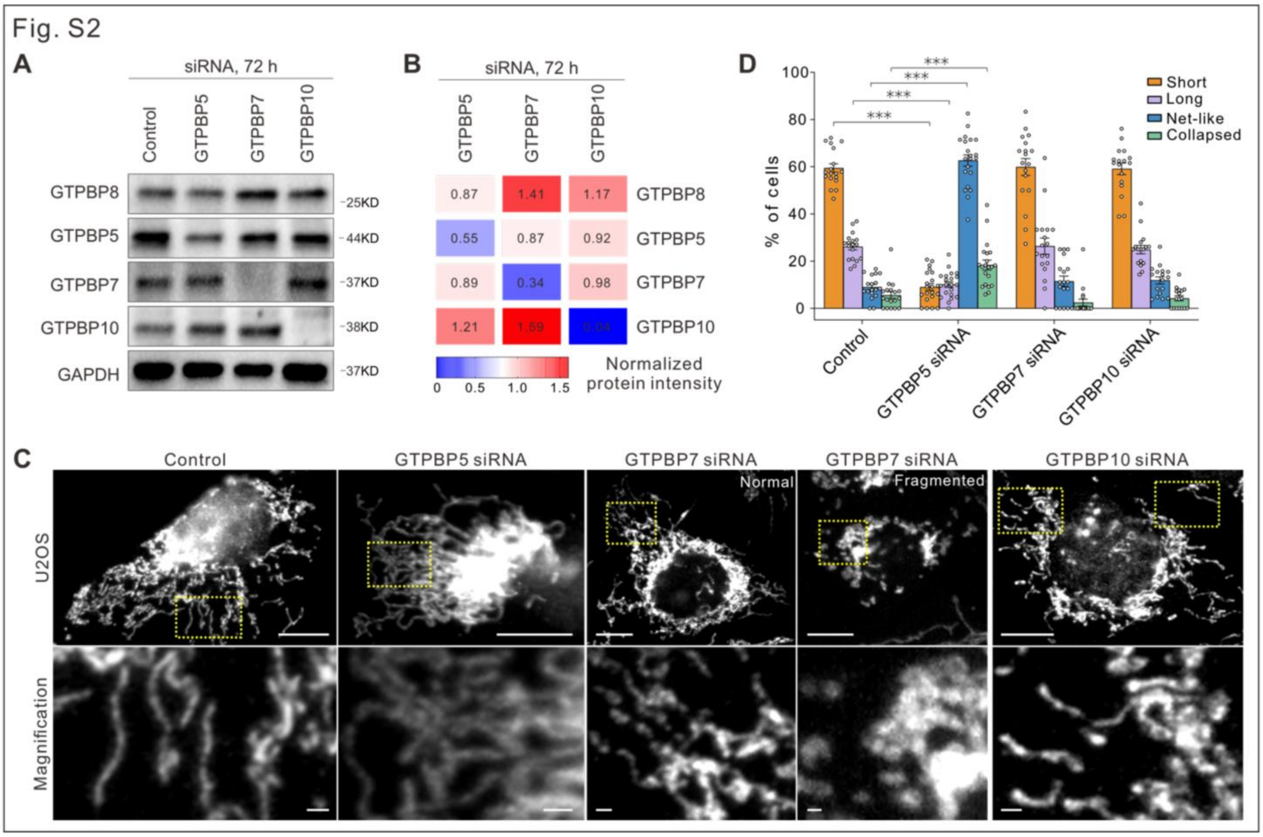

2) Furthermore, we examined the effects of GTPBP8 depletion on the localization and copy number of mtDNA, mitochondrial membrane potential, and the production of cellular reactive oxygen species. In contrast to control cells, GTPBP8 knockdown caused an increase in oxidative stress but had no effect on mitochondrial membrane potential, the copy number and localization of mtDNA (**Fig. 1I-1K**).

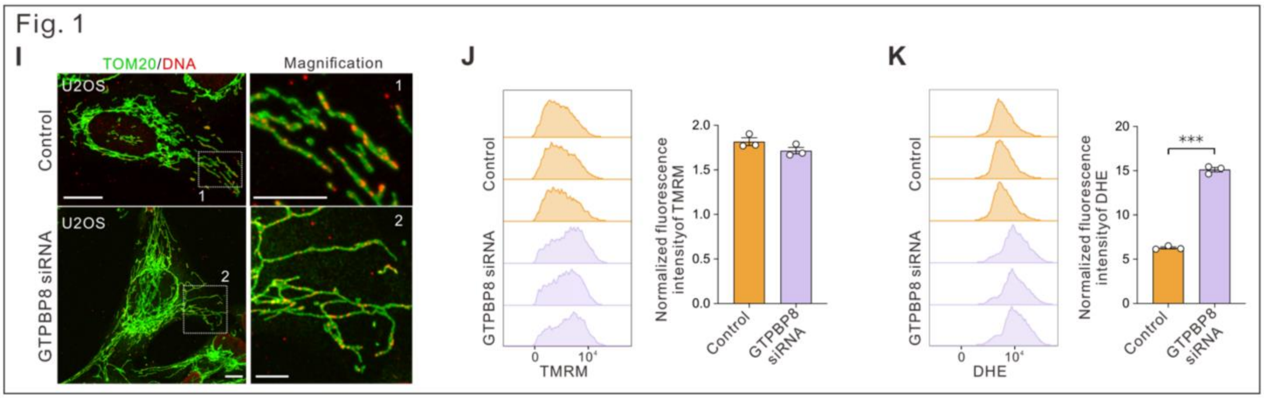

3) Drp1 activity is dependent on its activation by different post-translational modifications, such as phosphorylation and SUMOylation (Figueroa-Romero et al., 2009; Xu et al., 2016a). Phosphorylation of Drp1 at Ser616 can be modulated by different protein kinases involved in distinct signaling pathways, including protein kinase C (PKC), CDK1/Cyclin B, or ROCK, to promote fission (Lima et al., 2018; Han et al., 2008). Conversely, phosphorylation of Drp1 at Ser637 by protein kinase A (PKA) inhibits fission (Han et al., 2008). To determine whether the up-regulated Drp1 S637-PO4 level in GTPBP8-depleted cells was regulated by those kinases, we examined the expression level of CDK1, Cyclin B, ROCK1, ERK, and PKA. As shown in **Fig. 5A and Fig. S5C,** GTPBP8 knockdown resulted in a reduced CDK1 and Cyclin B level, but an increased PKA level in U2OS cells. The expression levels of ROCK1 and ERK were unaffected following the depletion of GTPBP8. Conversely, GTPBP8 overexpression resulted in decreased PKA and increased Cyclin B (**Fig. 5D**). Together, our new data show that the changed mitochondrial morphology and dynamics in response

© 2024. Published by The Company of Biologists under the terms of the Creative Commons Attribution License (https://creativecommons.org/licenses/by/4.0/).

to the altered GTPBP8 level is mainly attributed to the modified phosphorylation state of Drp1 by regulating PKA and CDK1/Cyclin B.

Collectively, our new data point to the possibility that GTPBP8 depletion caused defects in the assembly of mitoribosomal large subunit (mtLSU), resulting in compromised mitochondrial translation and, consequently, impaired mitochondrial function. This mitochondrial dysfunction might further regulate cell signaling involved in the phosphorylation of Drp1, leading to the increased phosphorylation of Drp1 at Ser637, the reduced recruiting of Drp1 to mitochondria, and eventually decreased mitochondrial fission.

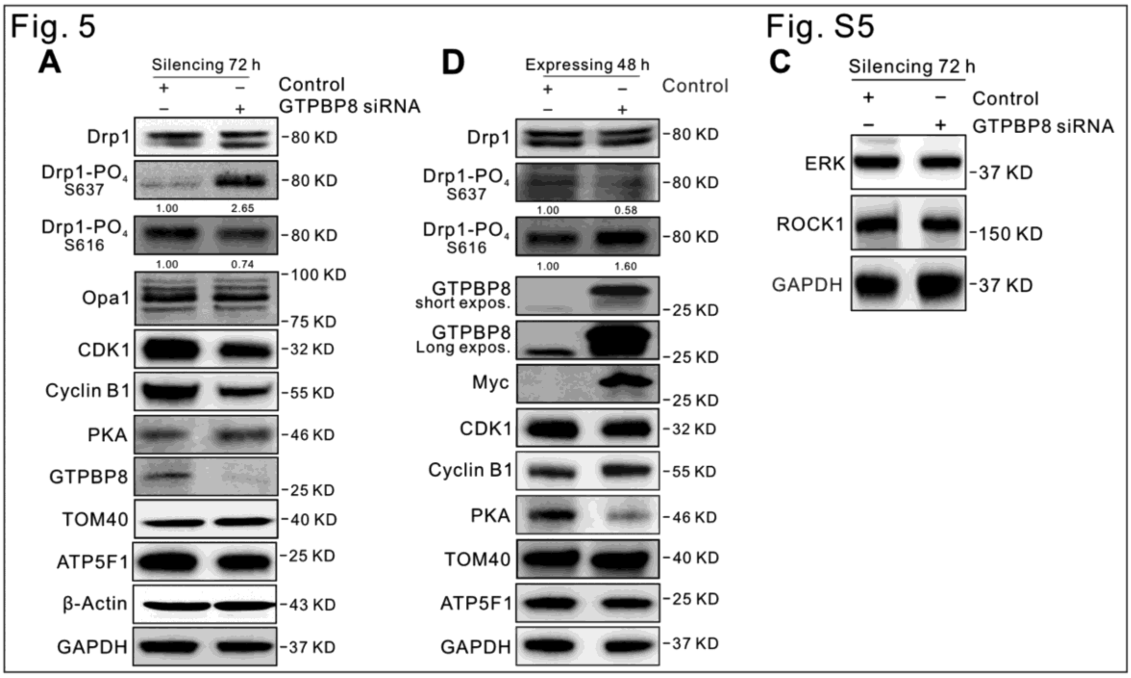

Furthermore, on Line 310 – "In this study, we unraveled the functional significance of a protein" which I believe is an overstatement. They do not know how GTPBP8 impacts Drp1 phosphorylation or even what part of its function is needed to impact fission, but as they point out further down in the discussion it is unlikely through direct interaction. They even postulate that it could be a completely indirect effect as they have also found that GTPBP8 has a role in mitochondrial ribosomal assembly and therefore mitochondrial translation.

Reply: We appreciate the reviewer's critical comments. To avoid the overstatement of the result, we revised the text in Line 310 as follows: In this study, we unraveled the function of a protein belonging to the P-loop_NTPase superfamily, previously uncharacterized, and termed it GTPBP8 (**Page 14, Line 364**).

To investigate how GTPBP8 depletion caused mitochondrial elongation, we further detected mitochondrial mtDNA copy number and localization, mitochondrial membrane potential, cellular oxidative stress, the expression level of CDK1, Cyclin B and PKA (**Fig. 1I-K, 5A, and 5D**). Collectively, these results point to the possibility that GTPBP8 depletion caused defects in the assembly of mtLSU, resulting in compromised mitochondrial translation and, consequently, impaired mitochondrial function. This mitochondrial dysfunction further regulate cell signaling involving in the phosphorylation of Drp1, leading to the increased phosphorylation of Drp1 at Ser637, the reduced recruiting of Drp1 to mitochondria, and eventually decreased mitochondrial fission.

Other comments:
Line 163 Is there any quantification to say that fission and fusion events are changed as stated on Line 163 (Fig 2A)? From the figure there is (3 fission, 1 fusion) events in the control, while there are (2 fission, 2 fusion) events in the GTPBP8 siRNA, but these small number of events are obviously not significantly different. Is there more data than this?

Reply: We appreciate the reviewer's valuable comments. The previous representative images were presented with a 20-second interval. Hence, some fission or fusion events might not be shown in the images. In response to the suggestions, we have displayed all images in each time interval (**Fig. 2A**). Moreover, we counted the fission and fusion events manually within 10 minutes of

© 2024. Published by The Company of Biologists under the terms of the Creative Commons Attribution License (https://creativecommons.org/licenses/by/4.0/).

mitochondrial tracking. The ratio of fission and fusion events was calculated as using the following formulas:

Ratio of fission = The total number of fission events / the total number of fission and fusion events *100

Ratio of fusion = The total number of fusion events / the total number of fission and fusion events *100

Compared to the balanced fission and fusion in the control cells, the frequency of fission events was reduced after silencing GTPBP8 in U2OS cells (**Fig. 2B**). These data suggest that the elongated mitochondrial morphology in the GTPBP8 depleted cells was mainly attributed to an imbalanced mitochondrial dynamic but not a loss of fission or fusion capabilities.

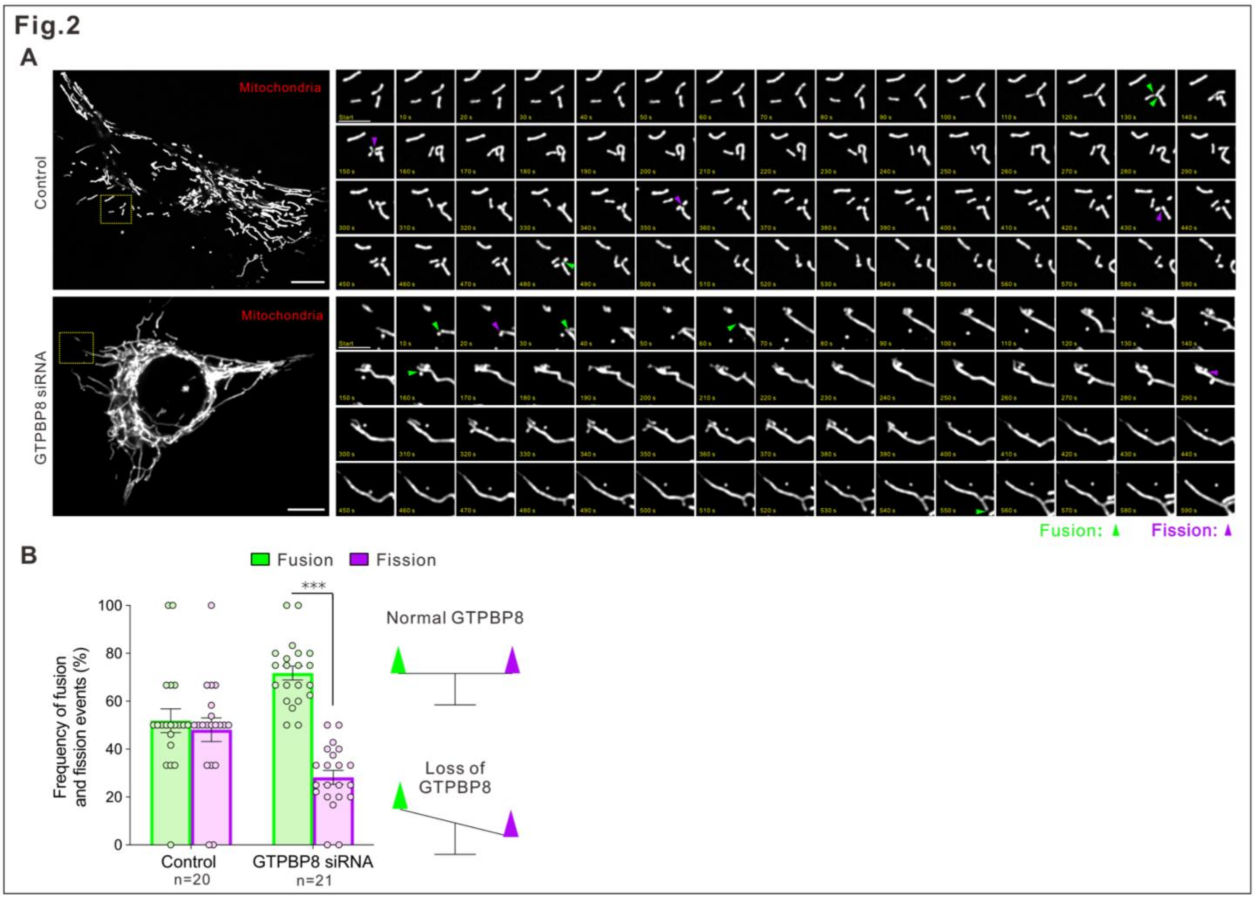

Line 217 – similarly it seems like it is hard to conclude a change in fission and fusion events from two events in the control (1 fission, 1 fusion) which was the same ratio previously said to be higher fusion in the GTPBP8 siRNA. The GTPBP8 OE seems to have (3 fission, 3 fusion) which doesn't change the ratio, though it is hard to see all the arrows indicating events in these images. It also seems hard for the authors to conclude even more overall events with such low numbers.

Reply: We appreciate the reviewer's valuable comments. The previous representative images were presented with a 20-second interval. Hence, some fission or fusion events might not be shown in the images. In response to the suggestions, we have displayed all images in each time interval (**Fig. 3K**). Moreover, we manually counted fission and fusion events within a 10-minute period of mitochondrial tracking. The ratio of fission to fusion events was calculated based on these counts. A balanced fission and fusion were observed in control cells. However, GTPBP8 overexpression led to an increased frequency of mitochondrial fission (**Fig. 3L**), suggesting that the fragmented mitochondrial morphology in cells overexpressing GTPBP8 is primarily attributed to an imbalanced mitochondrial fusion and fission, rather than a loss of fission or fusion capabilities.

© 2024. Published by The Company of Biologists under the terms of the Creative Commons Attribution License (https://creativecommons.org/licenses/by/4.0/).

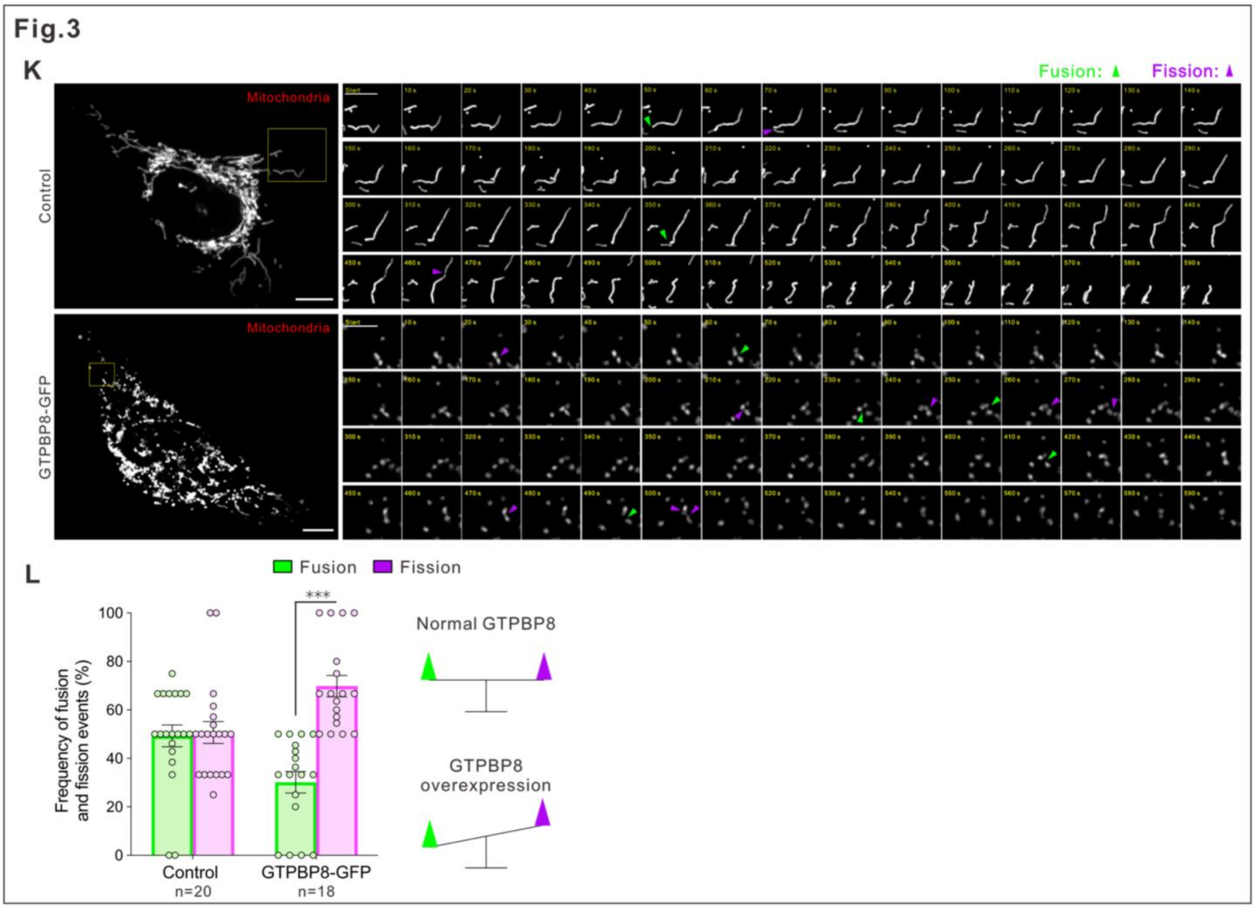

Minor typo comments:
Line 113 – Missing “subtilis”
Reply: *We appreciate the reviewer's careful review. In the revised manuscript, we have added 'subtilis' as suggested (**Page 6, Line 116**).*

Line 118 – proper citation of bioRxiv paper.
Reply: *We appreciate the reviewer's valuable comment. The preprint paper, entitled “GTPBP8 is Required for Mitoribosomal Biogenesis and Mitochondrial Translation” is now published on the Journal of* Cellular and Molecular Life Sciences. *We have cited this updated paper in the revised manuscript.*

Line 568, 574, 628 – Duplicated references
Reply: We appreciate the reviewer's meticulous attention to detail and careful checking. The duplicated references in the reference section have been thoroughly reviewed and corrected in the revised manuscript.

Second decision letter

MS ID#: JOCES/2023/261612

MS TITLE: GTPBP8 Regulates Mitochondrial Fission through a Drp1-Dependent Process

AUTHORS: Xiumei He, Liang Wang, Hoi Ying Tsang, xiaonan liu, Xiaofeng Yang, Ziqi Guo, Cheng Yang, Qiong Wu, Zuping Zhou, Xiaobo Cen, and Hongxia Zhao
ARTICLE TYPE: Research Article

We have now reached a decision on the above manuscript.

© 2024. Published by The Company of Biologists under the terms of the Creative Commons Attribution License (https://creativecommons.org/licenses/by/4.0/).

To see the reviewers' reports and a copy of this decision letter, please go to: https://submit-jcs.biologists.org and click on the 'Manuscripts with Decisions' queue in the Author Area. (Corresponding author only has access to reviews.)

As you will see, the reviewers gave favourable reports but raised some critical points that will require amendments to your manuscript before we can accept your paper for publication. Please address the minor comments of reviewers 1 and 2. This will require rewording parts of your paper according to the reviewers' recommendations.

Please ensure that you clearly highlight all changes made in the revised manuscript. Please avoid using 'Tracked changes' in Word files as these are lost in PDF conversion.

I should be grateful if you would also provide a point-by-point response detailing how you have dealt with the points raised by the reviewers in the 'Response to Reviewers' box. Please attend to all of the reviewers' comments. If you do not agree with any of their criticisms or suggestions please explain clearly why this is so.

Reviewer 1

Advance summary and potential significance to field

In the manuscript, He et al have provided additional quantifications and other experimental evidence that improves the rigor of the work and support their findings that GTPBP8 expression levels correlate with mitochondrial connectivity by influencing Drp1-mediated fission. The conclusions of the manuscript are also toned down compared to the original submission and more appropriately placed in the context of recently published work from their research group. I recommend publication of the manuscript, though feel that the word “regulate” in the title and abstract is not sufficiently supported by the data shown. It would be more accurate to state the GTPBP8 “influences” or “modulates” mitochondrial fission, as there is no evidence that the protein actively regulates the process. Instead, the data are more consistent with the model that mitochondrial morphology differences are indirectly influenced by too much or little activity of the protein.

Comments for the author

see above

Reviewer 2

Advance summary and potential significance to field

The authors have presented a significantly revised manuscript that addresses the shortcomings of the initial draft and bolsters some of the findings, such as the inclusion of data showing increased cellular ROS in GTPBP8 knockdown. The tempering of conclusions which directly connect GTPBP8 to DRP1 were appreciated, as while it is clear disruption of GTPBP8 leads to alterations in DRP1 phosphorylation, it was not clear these were direct but instead consequential. Overall, the new manuscript addresses all critiques and only minor concerns remain.

Comments for the author

1) The blot in Fig 5A for PKA is likely a technical error but requires re-evaluation. First, expression appears different between the two control populations. The densitometry is noticeably weaker in the siRNA plot compared to the overexpression plot. This limits the conclusions that can be derived from the siRNA treatments. While it is possible that PKA is increased in GTPBP8 depletion, it is not unambiguously clear from the blot. This is in contrast to the over expression dataset, where PKA is noticeably decreased.

© 2024. Published by The Company of Biologists under the terms of the Creative Commons Attribution License (https://creativecommons.org/licenses/by/4.0/).

2) Significant digits. The authors have already reduced their significant digits in the revised version, however more may be required. For example, in the statement "Furthermore, the introduction of GTPBP8-GFP expression resulted in an augmentation of mitochondrial width, transitioning from an average width of 0.4619 μm in the control group to 0.5223 μm", are all these significant digits accurate within the limitations of the technique? If so, this would imply the authors have statistical confidence in measurements to 100 pm.

Reviewer 3

Advance summary and potential significance to field

The current manuscript “GTPBP8 Regulates Mitochondrial Fission through a Drp1-dependent process” by He et al explores the role of the mitochondrial localized GTPase GTPBP8 on mitochondrial dynamics. This paper does a good job of showing that up and downregulation of GTPBP8 has complementary effects on mitochondrial dynamics, where GTPBP8 knockdown causes mitochondrial elongation and GTPBP8 overexpression causes mitochondrial fragmentation. They are further able to connect alterations in GTPBP8 expression with changes in Drp1 phosphorylation. They also showed that knockdown of Drp1 rescued the mitochondrial fragmentation phenotype of GTPBP8 overexpression. These data point to a role of GTPBP8 in mitochondrial function and the need to further investigate the mechanism by which it impacts Drp1 to influence mitochondrial dynamics.

Comments for the author

While the authors have somewhat toned down their writing, some of the manuscript still implies that they have found a new direct mitochondrial fission factor on the inner membrane, which they do not show. How they have responded to their reviews presents a distinct story from how the manuscript is still written. For example from the reviewer response - "Collectively, these data point to the possibility that GTPBP8 depletion caused defects in the assembly of mtLSU resulting in compromised mitochondrial translation and, consequently, impaired mitochondrial function. This mitochondrial dysfunction could further initiate cell signaling involving in the phosphorylation of Drp1 at Ser637, the reduced recruiting of Drp1 to mitochondria, and eventually decreased mitochondrial fission." Yet from the discussion in the manuscript "For instance, the role of inner membrane proteins, particularly those on the matrix side of the inner membrane, in the fission process is not known. Additionally, the precise coordination between outer and inner membrane fission events and the factors involved are yet to be defined. In this study, we unraveled the function of a protein belonging to the P-loop_NTPase superfamily, previously uncharacterized and termed it GTPBP8....The function of GTPBP8 appears to be multifaceted and includes involvement in mitochondrial dynamics and ribosomal assembly. Our study revealed that GTPBP8 plays a critical role in regulating mitochondrial fission as evidenced by its impact on mitochondrial morphology and dynamics." This seems to imply that they have found a function for GTPBP8 that is distinct from its role in ribosomal assembly, when in their response to reviewers they are implying that the role in ribosomal assembly drives mitochondria function/dysfunction and secondarily mitochondrial dynamics.
While I believe they have made a lot of interesting findings, I still believe how the current manuscript is framed is not representative of the data presented.

Second revision

Author response to reviewers' comments

Point-by-Point Response to Reviewers’ Comments

We deeply appreciate the reviewer's careful examination. In response to these comments, we have replaced the PKA plot in Figure 5A and revised the description of "regulating" in the text according to the reviewer's suggestion. We have further refined the manuscript's narrative to enhance the

© 2024. Published by The Company of Biologists under the terms of the Creative Commons Attribution License (https://creativecommons.org/licenses/by/4.0/).

clarity and accessibility of our data. We believe that these additional works significantly improve the quality and comprehensiveness of our manuscript. Below, we present a detailed, point-by-point response to the reviewers' comments, with reviewer remarks shown in black and our responses in blue. We have highlighted the corresponding changes within the manuscript in red. We look forward to your feedback and the opportunity to have our work published in the *Journal of Cell Science*.

Reviewer 1 Advance Summary and Potential Significance to Field:

In the manuscript, He et al have provided additional quantifications and other experimental evidence that improves the rigor of the work and support their findings that GTPBP8 expression levels correlate with mitochondrial connectivity by influencing Drp1-mediated fission. The conclusions of the manuscript are also toned down compared to the original submission and more appropriately placed in the context of recently published work from their research group. I recommend publication of the manuscript, though feel that the word "regulate" in the title and abstract is not sufficiently supported by the data shown. It would be more accurate to state the GTPBP8 "influences" or "modulates" mitochondrial fission, as there is no evidence that the protein actively regulates the process. Instead, the data are more consistent with the model that mitochondrial morphology differences are indirectly influenced by too much or little activity of the protein.
Response: We thank the reviewer for the thoughtful comments and insights into our study. Our data show that the role of GTPBP8 in the regulation of mitochondrial fission is likely indirect but involves the fission signaling pathway. Additionally, the phosphorylation state of Drp1 changes in response to the level of GTPBP8. In accordance with the reviewer's suggestion, the title of the manuscript has been changed to "GTPBP8 Modulates Mitochondrial Fission through a Drp1-Dependent Process.". The word "regulate" in the main text has also been changed to "modulate" or "influence".

Reviewer 2 Advance Summary and Potential Significance to Field:
The authors have presented a significantly revised manuscript that addresses the shortcomings of the initial draft and bolsters some of the findings, such as the inclusion of data showing increased cellular ROS in GTPBP8 knockdown. The tempering of conclusions which directly connect GTPBP8 to DRP1 were appreciated, as while it is clear disruption of GTPBP8 leads to alterations in DRP1 phosphorylation, it was not clear these were direct but instead consequential. Overall, the new manuscript addresses all critiques and only minor concerns remain.
Response: We thank the reviewer for the thorough check and thoughtful comments.

Reviewer 2 Comments for the Author:
5) The blot in Fig 5A for PKA is likely a technical error but requires re-evaluation. First, expression appears different between the two control populations. The densitometry is noticeably weaker in the siRNA plot compared to the overexpression plot. This limits the conclusions that can be derived from the siRNA treatments. While it is possible that PKA is increased in GTPBP8 depletion, it is not unambiguously clear from the blot. This is in contrast to the over expression dataset, where PKA is noticeably decreased.
Response: We appreciate the reviewer's careful evaluation and valuable comments. We have completely overlooked this problem. All of the WB images in the manuscript are the original images, without changes in contrast or brightness. To be honest, the PKA antibody we used is not very effective in detecting endogenous PKA. The effects of the GTPBP8 level on the abundance of PKA were assessed in triplicate. All biological PKA plots of the GTPBP8-silenced and overexpressed samples are shown in the following figure. Furthermore, changes in PKA have also been quantified with these plots. The bar graphs show that PKA was statistically up-regulated and down-regulated upon the deletion and overexpression of GTPBP8, respectively.

© 2024. Published by The Company of Biologists under the terms of the Creative Commons Attribution License (https://creativecommons.org/licenses/by/4.0/).

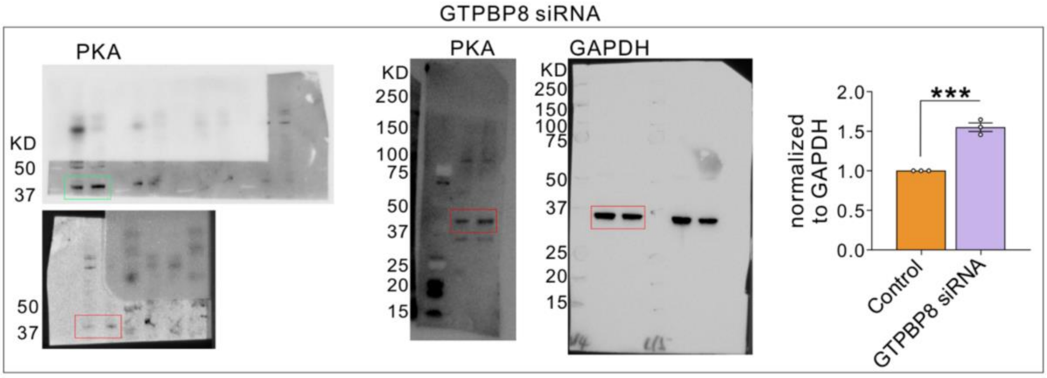

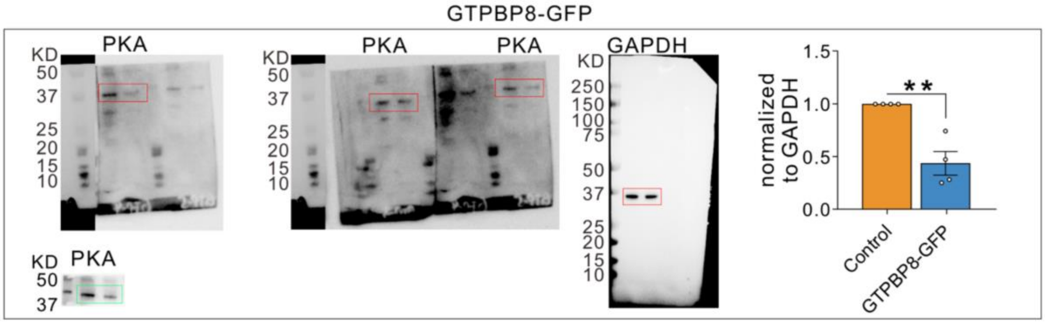

We greatly appreciate the reviewer's careful examination. The plots marked with green rectangles are now presented in the main figures. We believe that the new PKA plot in Figure 5A makes it more evident.

6) Significant digits. The authors have already reduced their significant digits in the revised version, however, more may be required. For example, in the statement "Furthermore, the introduction of GTPBP8-GFP expression resulted in an augmentation of mitochondrial width, transitioning from an average width of 0.4619 µm in the control group to 0.5223 µm", are all these significant digits accurate within the limitations of the technique? If so, this would imply the authors have statistical confidence in measurements to 100 pm.
Response: The measured value of mitochondrial width only accurate to 3 decimal places from µm (single nm). The values of 0.4619 and 0.5223 represent the calculated mean values of mitochondrial width obtained from the statistical description in Graphpad. To avoid any potential confusion, the values of mitochondrial width in Figs. 1G and 3I have been presented with three decimal places, maintaining consistency with the measured data (Fig. 1G, **Page 7, Line 147-149;** Fig. 3I, **Page 10, Line 240-242**). We thank the reviewer pointing this out. This adjustment maintains a professional tone and ensures that the reader understands the rationale for presenting numerical values.

Reviewer 3 Advance Summary and Potential Significance to Field:

The current manuscript “GTPBP8 Regulates Mitochondrial Fission through a Drp1-dependent process” by He et al explores the role of the mitochondrial localized GTPase GTPBP8 on mitochondrial dynamics. This paper does a good job of showing that up and downregulation of GTPBP8 has complementary effects on mitochondrial dynamics, where GTPBP8 knockdown causes mitochondrial elongation and GTPBP8 overexpression causes mitochondrial fragmentation. They are further able to connect alterations in GTPBP8 expression with changes in Drp1 phosphorylation. They also showed that knockdown of Drp1 rescued the mitochondrial fragmentation phenotype of GTPBP8 overexpression. These data point to a role of GTPBP8 in mitochondrial function and the need to further investigate the mechanism by which it impacts Drp1 to influence mitochondrial dynamics.
Response: We thank the reviewer for the thorough check and thoughtful comments.

Reviewer 3 Comments for the Author:
While the authors have somewhat toned down their writing, some of the manuscript still implies

© 2024. Published by The Company of Biologists under the terms of the Creative Commons Attribution License (https://creativecommons.org/licenses/by/4.0/).

that they have found a new direct mitochondrial fission factor on the inner membrane, which they do not show. How they have responded to their reviews presents a distinct story from how the manuscript is still written. For example from the reviewer response - "Collectively, these data point to the possibility that GTPBP8 depletion caused defects in the assembly of mtLSU, resulting in compromised mitochondrial translation and, consequently, impaired mitochondrial function. This mitochondrial dysfunction could further initiate cell signaling involving in the phosphorylation of Drp1 at Ser637, the reduced recruiting of Drp1 to mitochondria, and eventually decreased mitochondrial fission." Yet from the discussion in the manuscript "For instance, the role of inner membrane proteins, particularly those on the matrix side of the inner membrane, in the fission process is not known. Additionally, the precise coordination between outer and inner membrane fission events and the factors involved are yet to be defined. In this study, we unraveled the function of a protein belonging to the P-loop_NTPase superfamily, previously uncharacterized, and termed it GTPBP8....The function of GTPBP8 appears to be multifaceted and includes involvement in mitochondrial dynamics and ribosomal assembly. Our study revealed that GTPBP8 plays a critical role in regulating mitochondrial fission, as evidenced by its impact on mitochondrial morphology and dynamics." This seems to imply that they have found a function for GTPBP8 that is distinct from its role in ribosomal assembly, when in their response to reviewers they are implying that the role in ribosomal assembly drives mitochondria function/dysfunction and secondarily mitochondrial dynamics.

While I believe they have made a lot of interesting findings, I still believe how the current manuscript is framed is not representative of the data presented.

Response: In accordance with the reviewer’s comments, we have carefully revised the manuscript to provide a more comprehensive and elucidating portrayal of the role of GTPBP8. We believe these revisions contribute to a clearer understanding of our findings.

Third decision letter

MS ID#: JOCES/2023/261612

MS TITLE: GTPBP8 Modulates Mitochondrial Fission through a Drp1-Dependent Process

AUTHORS: Xiumei He, Liang Wang, Hoi Ying Tsang, xiaonan liu, Xiaofeng Yang, Ziqi Guo, Cheng Yang, Qiong Wu, Zuping Zhou, Xiaobo Cen, and Hongxia Zhao
ARTICLE TYPE: Research Article

I am happy to tell you that your manuscript has been accepted for publication in Journal of Cell Science, pending standard ethics checks.

© 2024. Published by The Company of Biologists under the terms of the Creative Commons Attribution License (https://creativecommons.org/licenses/by/4.0/).
